# Three-Component Reactions of 3-Arylidene-3*H*-Indolium Salts, Isocyanides and Amines

**DOI:** 10.3390/molecules26092402

**Published:** 2021-04-21

**Authors:** Hung M. Nguyen, Nikita E. Golantsov, Alexandra S. Golubenkova, Victor B. Rybakov, Leonid G. Voskressensky

**Affiliations:** 1 Department of Organic Chemistry, Faculty of Science, Peoples’ Friendship University of Russia (RUDN University), 6 Miklukho-Maklaya St., 117198 Moscow, Russia; hunghoadhhv2008@gmail.com (H.M.N.); golubenkova_as@pfur.ru (A.S.G.); 2 Faculty of Natural Sciences, Hung Vuong University, Nguyen Tat Thanh Street, Viet Tri 35120, Vietnam; 3 Faculty of Chemistry, Lomonosov Moscow State University, 1-3 Leninskiye Gory, 119991 Moscow, Russia; rybakov20021@yandex.ru

**Keywords:** multicomponent reactions, isocyanides, carbenium ions, indoles, imidamides, imidazolones

## Abstract

A multicomponent reaction of isocyanides with aryl(indol-3-yl)methylium salts and amines has been found. A series of aryl(indol-3-yl)acetimidamides was obtained in up to 96% yields. In the case of ethyl isocyanoacetate, the reaction is followed by cyclization to form 3,5-dihydro-4*H*-imidazol-4-one derivatives.

## 1. Introduction

Isocyanide-based multicomponent reactions play an outstanding role in the syntheses of heterocycles [1,2], biologically relevant compounds [3,4,5,6,7] and for diversity-oriented synthesis [6,7,8,9]. In the case of the famous Ugi reaction, isocyanide interacts with iminium salt generated in situ from carbonyl compound and amine [10]. A number of transformations, in which isocyanide interacts with a previously prepared iminium salt or cyclic imines in the presence of protic acid, have been described [11,12,13,14,15]. The potency of methods, based on the Ugi reaction, increases with the possibility of subsequent modification or cyclization of obtained products of multicomponent reaction [16,17,18,19,20,21].

**Scheme 1:**
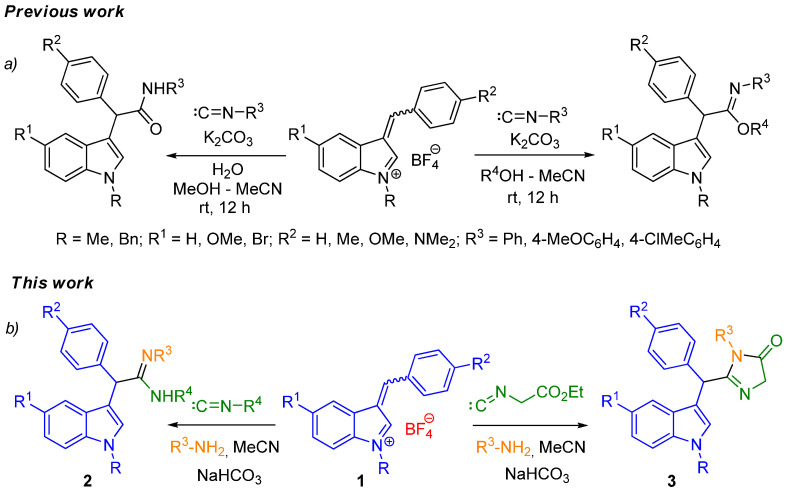
Reactions of 3-arylidene-3*H*-indolium salts with isocyanides: (**a**) our previous work; (**b**) this work.

Recently we have developed a method for the synthesis of alkyl aryl(indol-3-yl)acetimidates and aryl(indol-3-yl)acetamides based on a three-component reaction of isocyanides with such a specific class of electrophilic reagents as 3-arylidene-3*H*-indolium salts and oxygen-containing nucleophiles, water and alcohols (Scheme 1a) [22]. 3-Arylidene-3*H*-indolium salts can be considered as stabilized diarylmethylium ions or as simple vinylogous iminium ions, the latter being in better agreement with the data of X-ray structural analyses of these compounds [23,24]. The use of 3-arylidene-3*H*-indolium salts as partners in the reaction with isocyanides provides access to compounds possessing indole scaffold, privileged from the medicinal chemistry point of view [25].

Herein, we report a three-component reaction of 3-aryliden-3*H*-indolium salts **1** with isocyanides and amines to form aryl(indol-3-yl)acetimidamides **2** (Scheme 1b). In the case of isocyanoacetic ester the reaction can be accompanied by subsequent cyclization with the formation of imidazolone derivatives **3**. 

## 2. Results and Discussion

**Scheme 2:**
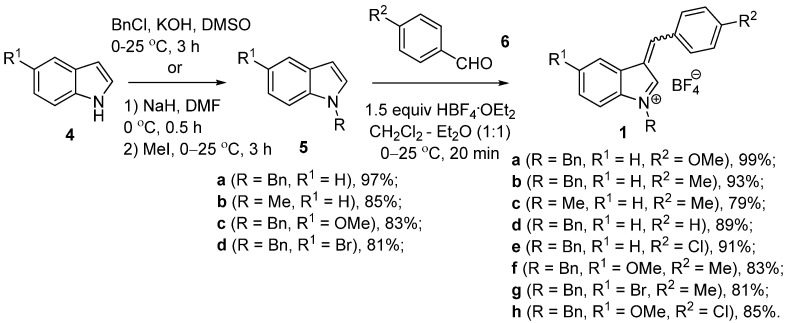
Synthesis of 3-arylidene-3*H*-indolium salts **1a**–**h**.

Starting salts **1a**–**h** were obtained by alkylation of the corresponding indoles **4** followed by reaction of *N*-alkylindoles **5a**–**d** with aromatic aldehydes **6** under conditions similar to previously published procedures (Scheme 2) [22,23,24].

**Scheme 3:**
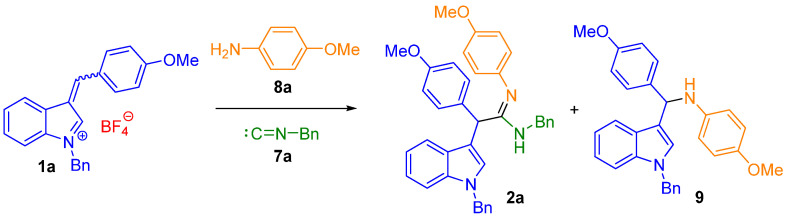
Reaction of salt **1a** with isonitrile **7a** and amine **8a**.

Next, the reaction of salt **1a** with benzyl isocyanide **7a** and *p*-anisidine (**8a**) was studied (Scheme 3; Table 1). In various solvents, including protic, aprotic non-polar and polar, the target product of three-component reaction, amidine **2a**, was formed with admixture of compound **9**, product of two-component reaction of the 3-arylidene-3*H*-indolium salt with the amine. The resulting compounds were isolated by chromatography as free bases after treatment of the reaction mixture with saturated NaHCO_3_ solution. Carrying out the reaction in acetonitrile medium made it possible to minimize the amount of the by-product **9**. In this case, target amidine **2a** was formed in good yield after 3 h at room temperature, and 12 h was required to complete the reaction. An attempt to accelerate the reaction by increasing the temperature was unsuccessful due to a tar formation and an increase in the amount of the by-product **9**.

**Scheme 4:**
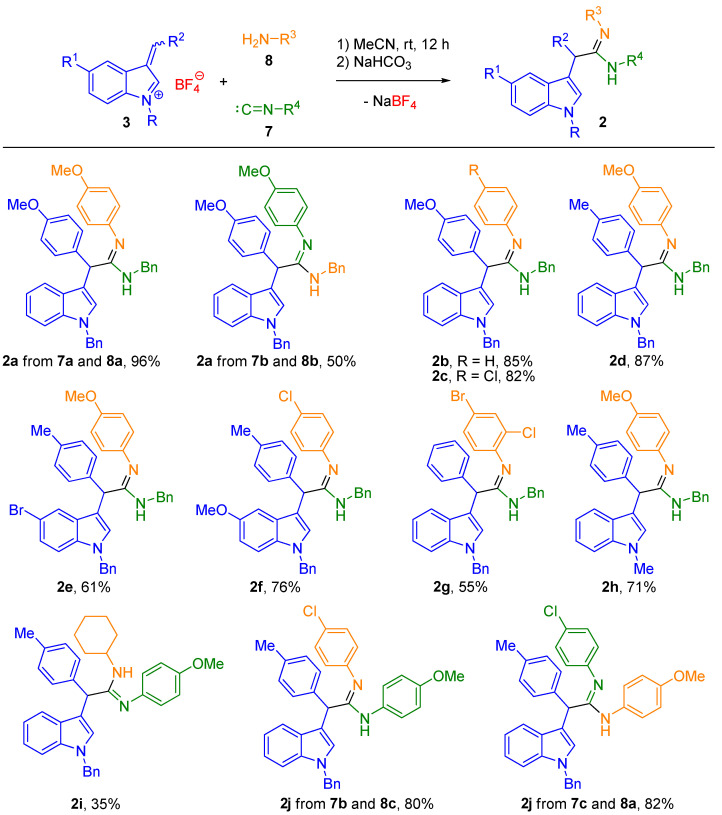
Synthesis of imidamides **2**.

According to the optimized procedure, a series of imidamides **2** was synthesized (Scheme 4). Compounds **2** containing an alkyl group together with an aryl group at the amidine nitrogen atoms are presented as the most stable tautomer according to NMR data [26], and compound **2j**—as a tautomeric mixture (for copies of NMR spectra, see Supplementary Materials). Interestingly, amidine **2a** was synthesized using two different combinations of isonitrile and amine, and the best yield was obtained by using benzyl isocyanide (**7a**) and *p*-anisidine (**8a**). Compound **2j** was also obtained by two methods, by using either *p*-methoxyphenylisocyanide (**7b**) and *p*-chloroaniline (**8c**) or *p*-chlorophenylisocyanide (**7c**) and amine **8a**. In this case both reagent combinations gave comparable results. The reaction with aliphatic cyclohexylamine, as well as with benzylamine (**8b**) proceeded with lower yield of target amidine **2i** due to side processes.

**Scheme 5:**
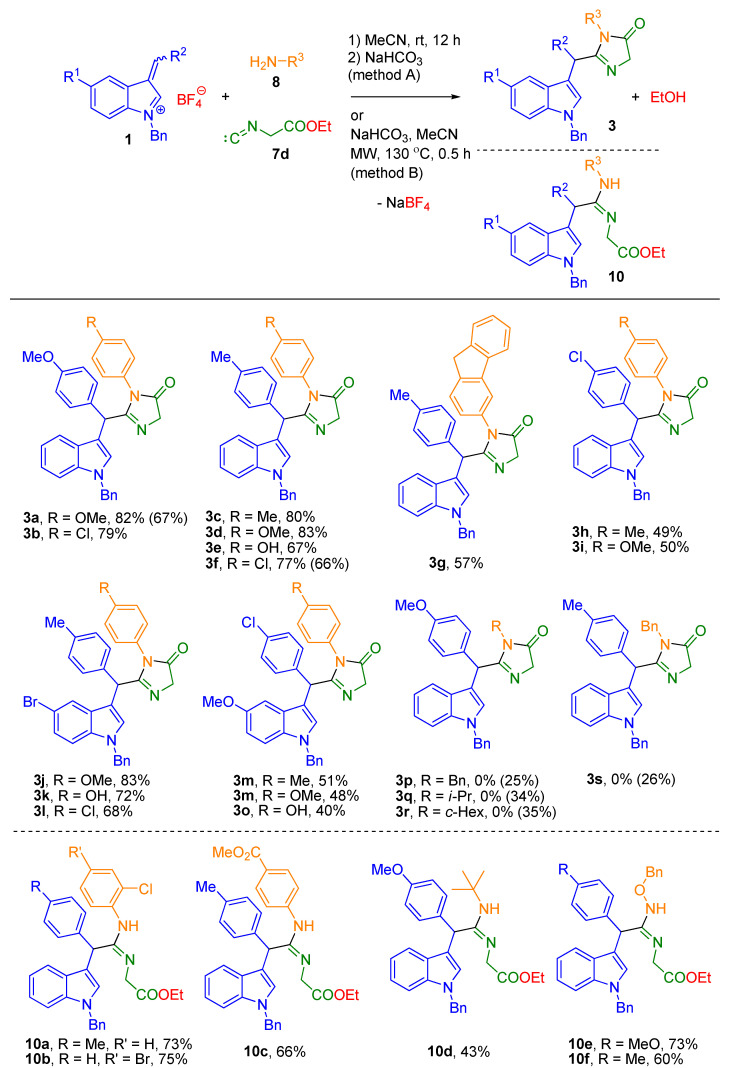
Reactions of 3-arylidene-3*H*-indolium salts **1** with isonitrile **7d** and amines **8**. The yields of imidazolones **3** obtained by method B are indicated in parentheses.

When ethyl isocyanoacetate **7d** was employed in this reaction with *p*-anisidine (**8a)**, a new product, imidazolone **3a**, was obtained in good yield (82%) instead of the corresponding imidamide (Scheme 5). Therefore, we continued to investigate the reaction of 3-arylidene-3*H*-indolium salts **1a**–**g** with isocyanoacetic ester **7d** and amines **9** in MeCN at room temperature (method A). Thus, we have obtained a series of imidazolones **3a**–**o** using various aromatic amines (Scheme 5). The exceptions were sterically hindered ortho-substituted anilines and weakly nucleophilic ester of para-aminobenzoic acid, in these cases, cyclization did not occur, and amidines **10a**–**c** containing ester group were isolated. We also failed to isolate the desired cyclization products with aliphatic amines. In the case of benzylamine, isopropylamine and cyclohexylamine a complex mixture of products that did not contain imidazolones **3p**–**s** was formed, while sterically hindered tert-butylamine gave acyclic imidamide **10d**. To our surprise, the reaction with *O*-benzylhydroxylamine led to the formation of only acyclic imidamides **10e**,**f**, represented by a mixture of tautomers in a 1:1 ratio. It was possible to obtain imidazolones **3p**–**o** using aliphatic amines (with the exception of tert-butylamine) with a low yield, when the reaction was carried out in a closed vessel under microwave irradiation at 130 °C (method B). The latter method, however, showed lower efficiency in comparison with the initial, method A, for the synthesis of imidazolones **3a**,**f** from aromatic amines, and at the same time, it did not allow to obtain the corresponding cyclization products involving sterically hindered amines and *O*-benzylhydroxylamine.

**Figure 1:**
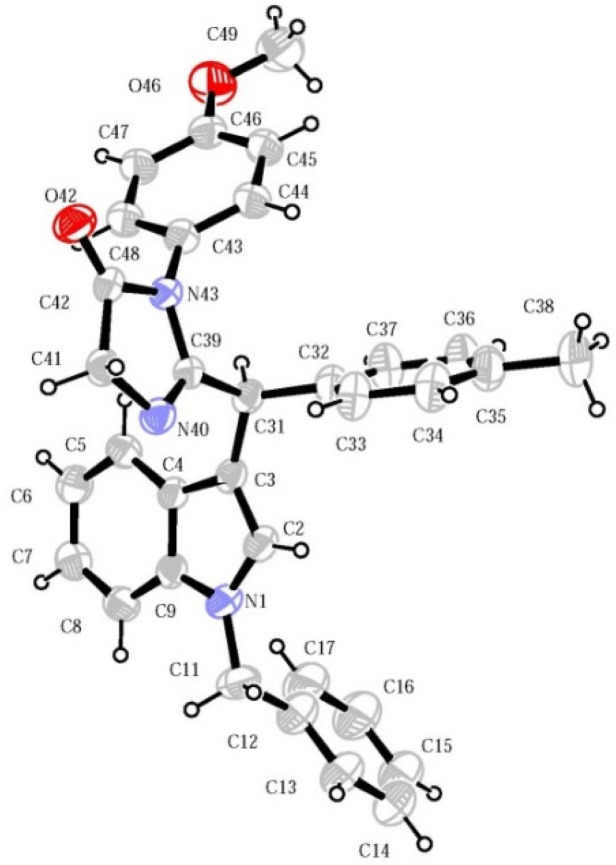
Crystal structure of imidazolone **3d**.

The structure of compound **3d** was unambiguously confirmed by X-ray structural analysis (Figure 1) [27].

**Scheme 6:**
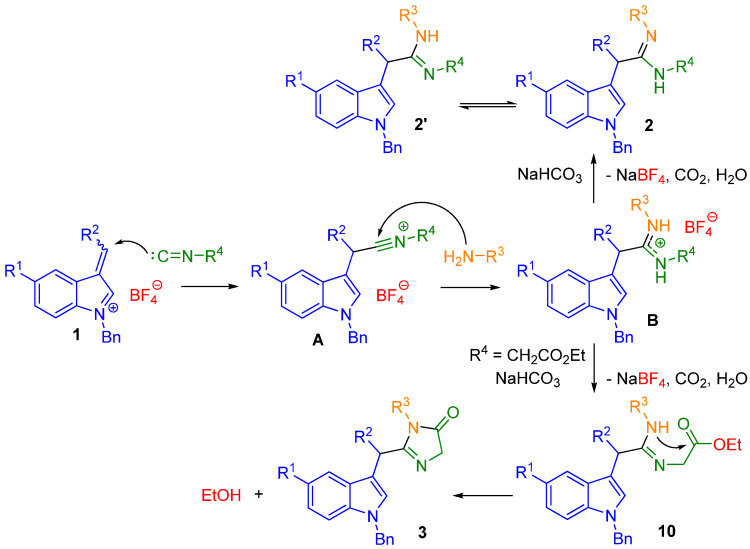
Proposed mechanism for the reactions of 3-arylidene-*3H*-indolium salts **1** with isocyanides **7** and amines **8**.

Next, it is necessary to discuss the proposed mechanism of discovered transformations based on our observations and previous studies (Scheme 6) [22,23]. Isonitrile initially attacks 3-arylidene-3*H*-indolium salt, which acts as an electrophilic reagent, vinylogous iminium 3*H*-indolium ion. The formed nitrilium salt **A** then reacts with amine leading to amidinium tetrafluoroborate **B**, which upon treatment with NaHCO_3_ gives a mixture of tautomeric imidamides **2** and **2’**. In the absence of steric or electronic restrictions imidamides **10** containing ester group undergo spontaneous cyclization leading to imidazolones **3**.

## 3. Materials and Methods

### 3.1. General Information

Starting reagents were purchased from commercial sources and were used without any additional purification or were prepared according to literature procedures. ^1^H and ^13^C NMR spectra were acquired on a Jeol JNM-ECA 600 spectrometer (Jeol Ltd, Tokyo, Japan) (with operating frequencies of 600 and 150 MHz, respectively) at room temperature and referenced to the residual signals of the solvent. The solvent used for NMR was CDCl_3_. Chemical shifts are reported in parts per million (δ/ppm). Coupling constants are reported in Hertz (*J*/Hz). The peak patterns are indicated as follows: s, singlet; d, doublet; t, triplet; q, quadruplet; m, multiplet; dd, doublet of doublets and br s, broad singlet. Infrared spectra were measured on an Infralum FT-801 FT/IR instrument. The wavelengths are reported in reciprocal centimeters (ν_max_/cm^−1^). Mass spectra were recorded with LCMS-8040 Triple quadrupole liquid chromatograph mass-spectrometer from Shimadzu (ESI) (Shimadzu, Tokyo, Japan). HRMS spectra were recorded on a Bruker MicrOTOF-Q II. Elemental analysis was performed on Euro Vector EA-3000 elemental analyzer. The reaction progress was monitored by TLC and the spots were visualized under UV light (254 or 365 nm). Column chromatography was performed using silica gel (230–400 mesh). Melting points were determined on SMP-10 apparatus and were uncorrected. Solvents were distilled and dried according to standard procedures.

### 3.2. Synthesis of Compounds ***1a**–**h***

Known tetrafluoroborates **1a**–**f** along with two new salts **1g,h** were prepared according to literature procedures [22,23,24].

*1-Benzyl-3-(4-chlorobenzylidene)-3H-indolium Tetrafluoroborate* (**1g**). Bright orange solid; yield 1.83 g (91% from 1.00 g 1-benzyl-1*H*-indole); mp 200–202 °C (dec.). IR (KBr): 3118, 3071, 3025, 1813, 1607, 1581, 1530, 1495, 1448, 1371, 1253, 1192, 1088, 1058, 948, 831, 859, 713, 638, 578 cm^–1^. ^1^H NMR (600 MHz, CDCl_3_ + TFA; ~ 1.5:1 *Z*/*E* diastereomeric mixture): δ = 9.13 (s, 1.5H), 8.93 (s, 1H), 8.82 (s, 1.5H), 8.68 (s, 1H), 8.24 (d, *J* = 7.7 Hz, 1H), 8.03 (d, *J* = 7.7 Hz, 1.5H), 7.97 (d, *J* = 8.6 Hz, 2H), 7.87 (d, *J* = 8.6 Hz, 3H), 7.69–7.57 (m, 12.5H), 7.48–7.37 (m, 12.5H), 5.71 (s, 3H), 5.62 (s, 2H). ^13^C NMR (150 MHz, CDCl_3_ + TFA): δ = 163.1, 161.4, 157.6, 154.2, 144.4, 143.4, 143.0, 140.5, 135.1, 134.4, 132.0, 131.8, 131.3, 131.2, 130.9, 130.6, 130.5, 130.4, 130.3, 130.2, 130.1, 130.0, 128.9, 128.7, 128.4, 128.2, 125.0, 124.3, 121.6, 117.3, 115.5, 115.4, 115.37, 113.5, 111.7, 55.2, 54.6. MS (ESI): *m*/z = 330 [M − BF_4_^–^]^+^; HRMS (TOF ES^+^): *m*/*z* [M − BF_4_^–^]^+^ calcd for C_22_H_17_ClN^+^: 330.1049; found: 330.1051.

*1-Benzyl-5-methoxy-3-(4-chlorobenzylidene)-3H-indolium Tetrafluoroborate* (**1h**). Brownish solid; yield 1.60 g (85% from 1.00 g 1-benzyl-5-methoxy-1*H*-indole); mp 196–198 °C (dec.). IR (KBr): 3122, 2982, 2843, 1620, 1582, 1526, 1484, 1440, 1376, 1298, 1264, 1234, 1192, 1091, 1053, 1032, 965, 856, 825, 756, 705, 646 cm^–1^. ^1^H NMR (600 MHz, CDCl_3_+TFA; ~ 2.55:1 *Z*/*E* diastereomeric mixture): δ = 8.98 (s, 2.55H), 8.81 (s, 1H), 8.73 (s, 2.55H), 8.61 (s, 1H), 7.93 (d, *J* = 8.5 Hz, 2H), 7.84 (d, *J* = 8.5 Hz, 5.10H), 7.71 (d, *J* = 2.2 Hz, 1H), 7.62 (d, *J* = 8.5 Hz, 2H), 7.56 (d, *J* = 8.5 Hz, 5.10H), 7.50 (d, *J* = 2.3 Hz, 2.55H), 7.46–7.35 (m, 21.30H), 7.11 (dd, *J* = 9.0, 2.2 Hz, 1H), 7.07 (dd, *J* = 9.0, 2.3 Hz, 2.55H), 5.63 (s, 5.10H), 5.55 (s, 2H), 3.93 (s, 7.65H), 3.85 (s, 3H). ^13^C NMR (150 MHz, CDCl_3_+TFA): δ = 161.9, 161.8, 161.6, 159.8, 156.5, 152.1, 143.8, 142.7, 134.9, 134.1, 133.9, 131.9, 131.6, 131.1, 130.7, 130.6, 130.4, 130.3, 130.1, 130.0, 129.9, 128.7, 128.6, 128.3, 117.3, 117.0, 116.5, 116.4, 116.0, 115.4, 113.5, 111.6, 110.4, 106.4, 56.4, 56.3, 55.2, 54.7. MS (ESI): *m*/z = 360 [M − BF_4_^–^]^+^; HRMS (TOF ES^+^): *m/z* [M − BF_4_^–^]^+^ calcd for C_23_H_19_ClNO^+^: 360.1155; found: 360.1160.

### 3.3. Synthesis of Imidamide **2**

Isocyanide **7** (0.65 mmol) and amine **8** (0.6 mmol) were dissolved in abs. MeCN (5 mL). A salt **1** (0.5 mmol) was then added. The reaction mixture was stirred at r.t. for 12 h and concentrated in vacuo. The residue was dissolved in EtOAc (50 mL), washed with NaHCO_3_ (2 × 25 mL), brine (20 mL), and dried over anhydrous Na_2_SO_4_. The EtOAc was evaporated in vacuo. The residue was chromatographed on a column with silica gel with EtOAc-hexane.

The following compounds were prepared:

*N-Benzyl-2-(1-benzyl-1H-indol-3-yl)-N’,2-bis(4-methoxyphenyl)acetimidamide* (**2a**). Brownish oil; yield 271 mg (96%) from **7a**, **8a** and 170 mg (50%) from **7b**, **8b**; *R_f_* = 0.69 (EtOAc-hexane, 1:3). IR (KBr): 3413, 3030, 2932, 2832, 1671, 1629, 1497, 1466, 1356, 1334, 1238, 1177, 1101, 1030, 836, 741, 698 cm^–1^. ^1^H-NMR (600 MHz, CDCl_3_), δ = 7.39 (d, *J* = 7.9, 1H), 7.24–7.17 (m, 8H), 7.13 (d, *J* = 8.6, 2H), 7.11–7.07 (m, 3H), 6.97–6.93 (m, 2H), 6.82 (d, *J* = 8.6 Hz, 2H), 6.73 (d, *J* = 8.8 Hz, 2H), 6.66 (s, 1H), 6.65 (d, *J* = 8.8 Hz, 2H), 5.44 (s, 1H), 5.22 (d, *J* = 16.2 Hz, 1H), 5.18 (d, *J* = 16.2 Hz, 1H), 4.75 (br s, 1H), 4.58–4.51 (m, 2H), 3.81 (s, 3H), 3.76 (s, 3H). ^13^C-NMR (150 MHz, CDCl_3_), δ = 158.6, 158.4, 155.0, 139.4, 137.6, 137.2, 132.3, 129.7 (2C), 128.9, 128.6 (2C), 128.5, 128.1, 127.8 (2C), 127.7, 127.3, 127.1, 126.5 (2C), 123.2 (2C), 122.6, 119.8, 119.6, 115.6, 115.0, 114.1 (2C), 114.0 (2C), 110.0, 55.6, 55.4, 50.1, 45.5, 43.5. MS (ESI): *m/z* = 566 [M + H]^+^; HRMS (TOF ES^+^): *m/z* [M + H]^+^ calcd for C_38_H_36_N_3_O_2_^+^: 566.2802; found: 566.2805.

*N-Benzyl-2-(1-benzyl-1H-indol-3-yl)-2-(4-methoxyphenyl)-N’-phenylacetimidamide* (**2b**). Brownish oil; yield 227 mg (85%); *R_f_* = 0.53 (EtOAc-hexane, 1:5). IR (KBr): 3417, 3055, 3028, 2930, 2834, 1947, 1885, 1631, 1591, 1509, 1483, 1356, 1334, 1301, 1249, 1176, 1070, 1029, 906, 801, 738, 697 cm^−1^. ^1^H-NMR (600 MHz, CDCl_3_), δ = 7.42 (d, *J* = 7.9, 1H), 7.25–7.10 (m, 15H), 6.99–6.93 (m, 3H), 6.84 (d, *J* = 8.7 Hz, 2H), 6.78–6.73 (m, 2H), 6.67 (s, 1H), 5.44 (s, 1H), 5.24 (d, *J* = 16.2 Hz, 1H), 5.20 (d, *J* = 16.2 Hz, 1H), 4.82 (br s, 1H), 4.62-4.55 (m, 2H), 3.82 (s, 3H). ^13^C-NMR (150 MHz, CDCl_3_), δ = 158.6, 157.8, 151.0, 139.4, 137.5, 137.2, 132.3, 129.7 (2C), 128.9 (2C), 128.7, 128.6 (2C), 128.5, 127.8 (2C), 127.7 (2C), 127.3, 127.1, 126.5 (2C), 122.6, 122.5 (2C), 122.0, 119.8, 119.6, 115.4, 114.1 (2C), 110.0, 55.4, 50.1, 45.5, 43.6. MS (ESI): *m/z* = 536 [M + H]^+^; HRMS (TOF ES^+^): *m/z* [M + H]^+^ calcd for C_37_H_34_N_3_O^+^: 536.2696; found: 536.2695.

*N-Benzyl-2-(1-benzyl-1H-indol-3-yl)-N’-(4-chlorophenyl)-2-(4-methoxyphenyl)acetimidamide* (**2c**). Orange oil; yield 234 mg (82%); *R_f_* = 0.44 (EtOAc-hexane, 1:5). IR (KBr): 3418, 3060, 3028, 2929, 2835, 1951, 1885, 1807, 1629, 1587, 1509, 1482, 1356, 1334, 1301, 1249, 1177, 1089, 1030, 838, 740, 697 cm^−1^. ^1^H-NMR (600 MHz, CDCl_3_), δ = 7.39 (d, *J* = 7.9, 1H), 7.25 – 7.19 (m, 8H), 7.14 – 7.08 (m, 7H), 6.98–6.93 (m, 2H), 6.84 (d, *J* = 8.7 Hz, 2H), 6.66–6.62 (m, 3H), 5.37 (s, 1H), 5.23 (d, *J* = 16.2 Hz, 1H), 5.19 (d, *J* = 16.2 Hz, 1H), 4.87 (br s, 1H), 4.58-4.52 (m, 2H), 3.82 (s, 3H). ^13^C-NMR (150 MHz, CDCl_3_), δ = 158.7, 158.2, 147.7, 139.1, 137.5, 137.2, 132.0, 129.7 (2C), 128.9 (2C), 128.7 (2C), 128.6 (2C), 128.5, 127.8 (2C), 127.7, 127.2, 127.1, 127.0, 126.5 (2C), 123.8 (2C), 122.7, 119.9, 119.4, 115.2, 114.2 (2C), 110.1, 55.4, 50.1, 45.5, 43.8. MS (ESI): *m/z* = 570 [M + H]^+^; HRMS (TOF ES^+^): *m/z* [M + H]^+^ calcd for C_37_H_33_ClN_3_O^+^: 570.2306; found: 570.2304.

*N-Benzyl-2-(1-benzyl-1H-indol-3-yl)-N’-(4-methoxyphenyl)-2-(p-tolyl)acetimidamide* (**2d**). Brownish oil; yield 261 mg (87%); *R_f_* = 0.50 (EtOAc-hexane, 1:4). IR (KBr): 3415, 3058, 3030, 2921, 2832, 1672, 1628, 1497, 1466, 1453, 1356, 1334, 1237, 1201, 1177, 1101, 1029, 969, 832, 740, 697 cm^−1^. ^1^H-NMR (600 MHz, CDCl_3_), δ = 7.38 (d*, J* = 7.9, 1H), 7.24–7.16 (m, 8H), 7.12–7.06 (m, 7H), 6.97–6.94 (m, 2H), 6.73 (d, *J* = 8.8 Hz, 2H), 6.68 (s, 1H), 6.66 (d, *J* = 8.8 Hz, 2H), 5.47 (s, 1H), 5.23 (d, *J* = 16.2 Hz, 1H), 5.19 (d, *J* = 16.2 Hz, 1H), 4.75 (br s*,* 1H), 4.59-4.52 (m, 2H), 3.76 (s, 3H), 2.35 (s, 3H). ^13^C-NMR (150 MHz, CDCl_3_), δ = 158.3, 155.0, 139.5, 137.6, 137.2, 137.1, 137.0, 136.6, 129.4 (2C), 128.9 (2C), 128.6 (2C), 128.53 (2C), 128.50, 127.8 (2C), 127.7, 127.3, 127.1, 126.5 (2C), 123.2 (2C), 122.5, 119.8, 119.7, 115.4, 114.1 (2C), 110.0, 55.6, 50.1, 45.5, 43.9, 21.2. MS (ESI): *m/z* = 550 [M + H]^+^; HRMS (TOF ES^+^): *m/z* [M + H]^+^ calcd for C_38_H_36_N_3_O^+^: 550.2853; found: 550.2793.

*N-Benzyl-2-(1-benzyl-5-bromo-1H-indol-3-yl)-N’-(4-methoxyphenyl)-2-(p-tolyl)acetimidamide* (**2e**). Brownish oil; yield 192 mg (61%); *R_f_* = 0.46 (EtOAc-hexane, 1:4). IR (KBr): 3424, 3253, 3030, 2928, 1870, 1735, 1630, 1497, 1356, 1236, 1030, 830, 732, 697 cm^−1^. ^1^H-NMR (600 MHz, CDCl_3_), δ = 7.44 (d, *J* = 1.8 Hz, 1H), 7.25–7.20 (m, 7H), 7.16–7.12 (m, 2H), 7.12–7.05 (m, 5H), 6.96–6.90 (m, 2H), 6.73 (d, *J* = 8.8 Hz, 2H), 6.72 (s, 1H), 6.63 (d, *J* = 8.8 Hz, 2H), 5.40 (s, 1H), 5.20 (d, *J* = 16.2 Hz, 1H), 5.16 (d, *J* = 16.2 Hz, 1H), 4.65 (br s, 1H), 4.63-4.48 (m, 2H), 3.76 (s, 3H), 2.35 (s, 3H). ^13^C-NMR (150 MHz, CDCl_3_), δ = 157.9, 155.1, 144.1, 139.4, 137.1, 136.9, 136.8, 135.8, 129.6 (2C), 129.5, 129.0 (2C), 128.6 (2C), 128.5 (2C), 127.9, 127.8 (2C), 127.2, 126.7, 126.4 (2C), 125.5, 123.1 (2C), 123.3, 114.9, 114.2 (2C), 113.2, 111.6, 55.6, 50.4, 45.5, 43.6, 21.2. MS (ESI): *m/z* = 628 [M + H]^+^; HRMS (TOF ES^+^): *m/z* [M + H]^+^ calcd for C_38_H_35_BrN_3_O^+^: 628.1958; found: 628.1960.

*N-Benzyl-2-(1-benzyl-5-methoxy-1H-indol-3-yl)-N’-(4-chlorophenyl)-2-(p-tolyl)acetimidamide* (**2f**). Brownish oil; yield 222 mg (76%); *R_f_* = 0.49 (EtOAc-hexane, 1:5). IR (KBr): 3385, 3032, 2915, 1744, 1631, 1587, 1480, 1453, 1357, 1266, 1202, 1166, 1097, 1039, 901, 843, 823, 786, 737, 703, 644, 588 cm^−1^. ^1^H-NMR (600 MHz, CDCl_3_), δ = 7.25–7.19 (m, 6H), 7.14–7.08 (m, 9H), 6.96–6.91 (m, 2H), 6.84 (dd, *J* = 8.9, 2.4 Hz, 1H), 6.77 (d, *J* = 2.4 Hz, 1H), 6.64 (d, *J* = 8.5 Hz, 2H), 6.61 (s, 1H), 5.33 (s, 1H), 5.18 (d, *J* = 16.2 Hz, 1H), 5.14 (d, *J* = 16.2 Hz, 1H), 4.89 (br s, 1H), 4.60-4.51 (m, 2H), 3.78 (s, 3H), 2.36 (s, 3H). ^13^C-NMR (150 MHz, CDCl_3_), δ = 158.1, 154.4, 149.7, 139.2, 137.6, 136.9, 136.8, 132.4, 129.5 (2C), 129.1, 128.9 (2C), 128.8 (2C), 128.6 (2C), 128.5 (2C), 127.8 (2C), 127.7 (2C), 127.6, 127.2, 127.1, 126.5, 126.4 (2C), 114.7, 112.9, 111.0, 101.0, 55.9, 50.4, 45.6, 44.2, 21.2. MS (ESI): *m/z* = 584 [M + H]^+^; HRMS (TOF ES^+^): *m/z* [M + H]^+^ calcd for C_38_H_35_ClN_3_O^+^: 584.2463; found: 584.2470.

*N-Benzyl-2-(1-benzyl-1H-indol-3-yl)-N’-(4-bromo-2-chlorophenyl)-2-phenylacetimidamide* (**2g**). Brownish oil; yield 170 mg (55%); *R_f_* = 0.58 (EtOAc-hexane, 1:5). IR (KBr): 3418, 3063, 3031, 2918, 1947, 1888, 1810, 1706, 1634, 1601, 1529, 1494, 1452, 1359, 1256, 1199, 1175, 1080, 1028, 918, 846, 734, 689 cm^−1^. ^1^H-NMR (600 MHz, CDCl_3_), δ = 7.51–7.47 (m, 2H), 7.30–7.26 (m, 3H), 7.25–7.21 (m, 7H), 7.21–7.14 (m, 5H), 7.10 (ddd*, J* = 8.0, 6.9, 1.1, 1H), 7.00–6.93 (m, 3H), 6.63 (d*, J* = 1.9 Hz, 1H), 6.28 (d, *J* = 8.5 Hz, 1H), 5.26–5.16 (m, 3H), 5.10 (br s, 1H), 4.64–4.57 (m, 2H). ^13^C-NMR (150 MHz, CDCl_3_), δ = 158.5, 147.1, 139.6, 138.9, 137.4, 137.2, 131.9, 130.7, 129.8, 128.9 (2C), 128.8 (2C), 128.7 (2C), 128.6 (2C), 127.8 (2C), 127.7 (2C), 127.4, 127.3, 127.2, 126.5 (2C), 125.5, 122.7, 120.0, 119.9, 117.0, 114.2, 110.1, 50.2, 45.8, 45.6. MS (ESI): *m/z* = 618 [M + H]^+^; HRMS (TOF ES^+^): *m/z* [M + H]^+^ calcd for C_36_H_30_BrClN_3_^+^: 618.1306; found: 618.1314.

*N-Benzyl-N’-(4-methoxyphenyl)-2-(1-methyl-1H-indol-3-yl)-2-(p-tolyl)acetimidamide* (**2h**). Brownish oil; yield 168 mg (71%); *R_f_* = 0.56 (EtOAc-hexane, 1:3). IR (KBr): 3418, 3060, 3028, 2929, 2835, 1951, 1885, 1807, 1629, 1587, 1509, 1482, 1356, 1334, 1301, 1249, 1177, 1089, 1030, 838, 740, 697 cm^−1^. ^1^H-NMR (600 MHz, CDCl_3_), δ = 7.40 (d, *J* = 7.9 Hz, 1H), 7.30–7.27 (m, 1H), 7.26–7.19 (m, 4H), 7.16–7.12 (m, 2H), 7.11–7.07 (m, 5H), 6.73 (d, *J* = 8.9 Hz, 2H), 6.65 (d, *J* = 8.9 Hz, 2H), 6.51 (d, *J* = 0.9 Hz, 1H), 5.42 (s, 1H), 4.81 (br s, 1H), 4.63-4.51 (m, 2H), 3.75 (s, 3H), 3.66 (s, 3H), 2.36 (s, 3H). ^13^C-NMR (150 MHz, CDCl_3_), δ = 158.4, 155.0, 144.3, 139.6, 137.5, 137.4, 136.5, 129.4 (2C), 129.0, 128.5 (2C), 128.4 (2C), 127.9 (2C), 127.1, 127.0, 123.3 (2C), 122.3, 119.5, 119.4, 114.7, 114.1 (2C), 109.4, 55.6, 45.5, 43.9, 32.9, 21.3. MS (ESI): *m/z* = 474 [M + H]^+^; HRMS (TOF ES^+^): *m/z* [M + H]^+^ calcd for C_32_H_32_N_3_O^+^: 474.2539; found: 474.2536.

*2-(1-Benzyl-1H-indol-3-yl)-N’-cyclohexyl-N-(4-methoxyphenyl)-2-(p-tolyl)acetimidamide* (**2i**) Orange oil; yield 68 mg (35%); *R_f_* = 0.40 (EtOAc-hexane, 1:5). IR (KBr): 3414, 3030, 2926, 2851, 1628, 1498, 1465, 1452, 1350, 1335, 1237, 1204, 1178, 1100, 1035, 835, 740, 695 cm^−1^. ^1^H-NMR (600 MHz, CDCl_3_), δ = 7.36 (d, *J* = 7.9 Hz, 1H), 7.30–7.26 (m, 3H), 7.26–7.22 (m, 2H), 7.20–7.14 (m, 1H), 7.11–7.04 (m, 5H), 7.02 (d, *J* = 7.5 Hz, 2H), 6.74–6.67 (m, 3H), 6.64 (s, 1H), 5.37 (s, 1H), 5.28 (d, *J* = 16.2 Hz, 1H), 5.23 (d, *J* = 16.2 Hz, 1H), 3.74 (s, 3H), 2.35 (s, 3H), 2.04–1.85 (m, 2H), 1.59–1.25 (m, 6H), 1.13–0.83 (m, 4H). ^13^C-NMR (150 MHz, CDCl_3_), δ = 155.7, 154.0, 138.6, 137.6, 137.4, 137.3, 137.1, 129.3 (2C), 128.9 (2C), 128.5, 128.4 (2C), 127.7, 127.2, 126.7, 126.5 (2C), 123.4, 122.5, 121.7, 119.7 (2C), 114.1 (2C), 109.9, 55.5, 50.1, 48.6, 43.9, 32.8, 32.6, 25.9, 24.7, 24.5, 21.2. MS (ESI): *m/z* = 542 [M + H]^+^; HRMS (TOF ES^+^): *m/z* [M + H]^+^ calcd for C_37_H_40_N_3_O^+^: 542.3166; found: 542.3166.

*2-(1-Benzyl-1H-indol-3-yl)-N-(4-chlorophenyl)-N’,2-bis(4-methoxyphenyl)acetimidamide* (**2j**). Brownish oil; yield 231 mg (80%) from **7b**, **8c** and 240 mg (82%) from **7c**, **8a**; *R_f_* = 0.51 (EtOAc-hexane, 1:5). IR (KBr): 3391, 3127, 3063, 3027, 2948, 2909, 2832, 2052, 1894, 1818, 1637, 1586, 1507, 1409, 1362, 1335, 1303, 1244, 1181, 1086, 1031, 969, 881, 839, 742, 695, 627, 562 cm^−1^. ^1^H-NMR (600 MHz, CDCl_3_), δ = 7.47–7.34 (m, 3H), 7.32–7.27 (m, 4H), 7.23–7.13 (m, 4H), 7.12–7.07 (m, 2H), 7.05 (d, *J* = 7.0 Hz, 2H), 6.85 (d, *J* = 8.7 Hz, 2H), 6.82–6.73 (m, 3H), 6.70–6.61 (m, 2H), 6.41 (s, 1H), 5.43 (s, 1H), 5.30 (d, *J* = 16.2 Hz, 1H), 5.26 (d, *J* = 16.2 Hz, 1H), 3.82 (s, 3H), 3.76 (s, 3H). ^13^C-NMR (150 MHz, CDCl_3_), δ = 158.8, 155.7, 155.5, 137.5, 137.3, 133.1, 131.9, 129.6 (2C), 129.0 (2C), 128.7 (2C), 128.6, 127.9 (2C), 127.1, 126.6 (2C), 123.3, 122.8, 122.4, 121.6 (2C), 120.6, 120.2, 119.5, 115.1, 114.3 (2C), 114.2 (2C), 110.2, 55.7, 55.4, 50.3, 44.4. MS (ESI): *m/z* = 586 [M + H]^+^; HRMS (TOF ES^+^): *m/z* [M + H]^+^ calcd for C_37_H_33_ClN_3_O_2_^+^: 586.2256; found: 586.2261.

*N-((1-Benzyl-1H-indol-3-yl)(4-methoxyphenyl)methyl)-4-methoxyaniline* (**9**). According to synthesis of imidamide (**2a**) without the isocyanide addition, compound **9** (90 mg, 40%) was obtained as brownish oil; *R_f_* = 0.63 (EtOAc– hexane, 1:7). IR (KBr): 3400, 3030, 3003, 2931, 2833, 1609, 1585, 1510, 1464, 1356, 1335, 1300, 1242, 1172, 1033, 819, 741, 702 cm^−1^. ^1^H-NMR (600 MHz, CDCl_3_), δ = 7.60 (d, *J* = 8.0 Hz, 1H), 7.44 (d, *J* = 8.7 Hz, 2H), 7.31–7.26 (m, 3H), 7.26–7.23 (m, 1H), 7.18 (ddd, *J* = 8.0, 7.0, 1.0 Hz, 1H), 7.11–7.06 (m, 3H), 6.89 (d, *J* = 8.7 Hz, 2H), 6.81 (s, 1H), 6.75 (d, *J* = 9.0 Hz, 2H), 6.58 (d, *J* = 9.0 Hz, 2H), 5.73 (s, 1H), 5.24 (s, 2H), 3.81 (s, 3H), 3.74 (s, 3H). ^13^C-NMR (150 MHz, CDCl_3_), δ = 158.8, 152.0, 142.3, 137.6, 137.2, 135.2, 128.9 (2C), 128.5 (2C), 127.7, 127.5, 126.9, 126.7 (2C), 122.2, 119.8, 119.6, 118.7, 114.9 (2C), 114.6 (2C), 114.0 (2C), 110.1, 56.2, 55.9, 55.4, 50.2. MS (ESI): *m/z* = 447 [M − H]^+^.

### 3.4. Synthesis of Imidazolone **3** and Synthesis Imidamides ***10a**–**f***

*Representative Procedure under Thermal Conditions (Method A).* Ethyl isocyanoacetate **7d** (0.65 mmol) and aromatic amine **8** (0.75 mmol) were dissolved in abs. MeCN (5 mL), and salt **1** (0.5 mmol) was then added. The reaction mixture was stirred at r.t. for 12 h and concentrated in vacuo. The residue was dissolved in EtOAc (50 mL), washed with NaHCO_3_ (2 × 25 mL), brine (20 mL), and dried over anhydrous Na_2_SO_4_. The EtOAc was evaporated in vacuo. The residue was chromatographed on a column with silica gel with EtOAc-hexane.

*Representative Procedure under Microwave Conditions* (Method B). Ethyl isocyanoacetate **7d** (0.65 mmol) and aromatic amine 8 (0.60 mmol) were dissolved in abs. MeCN (5 mL), salt **1** (0.5 mmol) and NaHCO_3_ (1.5 equiv.) were then added. The reaction mixture in closed vial was placed into microwave reactor and irradiated at 130 °C for 30 min. Upon reaction completion, the reaction mixture was concentrated in vacuo. The residue was dissolved in EtOAc (50 mL), washed with H_2_O (2 × 25 mL), brine (20 mL) and dried over anhydrous Na_2_SO_4_. The EtOAc was evaporated in vacuo. The residue was chromatographed on a column with silica gel with EtOAc-hexane.

The following compounds were prepared:

*2-((1-Benzyl-1H-indol-3-yl)(4-methoxyphenyl)methyl)-1-(4-methoxyphenyl)-1H-imidazol-5(4H)-one* (**3a**). Brownish oil; yield 211 mg (82%, method A) and 173 mg (67%, method B); *R_f_* = 0.25 (EtOAc– hexane, 1:1). IR (KBr): 3063, 2932, 2836, 1736, 1687, 1608, 1511, 1466, 1300, 1250, 1175, 1110, 1030, 830, 742, 698 cm^−1^. ^1^H-NMR (600 MHz, CDCl_3_), δ = 7.32–7.27 (m, 3H), 7.25–7.21 (m, 2H), 7.16–7.13 (m, 3H), 7.08–7.02 (m, 3H), 6.89 (s, 1H), 6.86 (d, *J* = 9.0 Hz, 2H), 6.83 (d, *J* = 9.0 Hz, 2H), 6.81 (d, *J* = 8.7 Hz, 2H), 5.28 (d, *J* = 16.2 Hz, 1H), 5.23 (d, *J* = 16.2 Hz, 1H), 5.15 (s, 1H), 4.39–4.29 (m, 2H), 3.80 (s, 3H), 3.78 (s, 3H). ^13^C-NMR (150 MHz, CDCl_3_), δ = 181.4, 167.1, 160.0, 158.9, 137.5, 137.0, 130.6, 129.9 (2C), 129.4 (2C), 128.9 (2C), 128.1, 127.8, 127.2, 126.8 (2C), 125.9, 122.3, 119.7, 119.1, 114.9 (2C), 114.1 (2C), 113.5, 110.2, 59.1, 55.7, 55.4, 50.3, 42.1. MS (ESI): *m/z* = 516 [M + H]^+^; HRMS (TOF ES^+^): *m/z* [M + H]^+^ calcd for C_33_H_30_N_3_O_3_^+^: 516.2281; found: 516.2283.

*2-((1-Benzyl-1H-indol-3-yl)(4-methoxyphenyl)methyl)-1-(4-chlorophenyl)-1H-imidazol-5(4H)-one* (**3b**). Brownish oil; yield 205 mg (79%, method A); *R_f_* = 0.25 (EtOAc– hexane, 1:1). IR (KBr): 3061, 2932, 2836, 1890, 1737, 1631, 1610, 1546, 1510, 1492, 1466, 1249, 1174, 1091, 1051, 1030, 992, 796, 739, 696, 635 cm^−1^. ^1^H-NMR (600 MHz, CDCl_3_), δ = 7.33–7.26 (m, 6H), 7.25 (d, *J* = 8.3 Hz, 1H), 7.18–7.16 (m, 1H), 7.15 (d, *J* = 8.7 Hz, 2H), 7.08–7.04 (m, 3H), 6.87 (d, *J* = 8.7 Hz, 2H), 6.85 (s, 1H), 6.82 (d, *J* = 8.8 Hz, 2H), 5.26 (d, *J* = 16.2 Hz, 1H), 5.22 (d, *J* = 16.2 Hz, 1H), 5.13 (s, 1H), 4.42–4.31 (m, 2H), 3.78 (s, 3H). ^13^C-NMR (150 MHz, CDCl_3_), δ = 180.8, 166.2, 159.0, 137.4, 137.0, 135.1, 131.9, 130.2, 129.8 (2C), 129.8 (2C), 129.4 (2C), 128.9 (2C), 128.0, 127.8, 127.0, 126.8 (2C), 122.4, 119.8, 119.0, 114.2 (2C), 113.0, 110.2, 59.1, 55.4, 50.3, 42.3. MS (ESI): *m/z* = 520 [M + H]^+^; HRMS (TOF ES^+^): *m/z* [M + H]^+^ calcd for C_32_H_27_ClN_3_O_2_^+^: 520.1786; found: 520.1791.

*2-((1-Benzyl-1H-indol-3-yl)(p-tolyl)methyl)-1-(p-tolyl)-1H-imidazol-5(4H)-one* (**3c**). Light pink solid; mp 186–188 °C; yield 192 mg (80%, method A); *R_f_* = 0.44 (EtOAc– hexane, 1:1). IR (KBr): 3025, 2914, 2866, 1901, 1730, 1632, 1542, 1513, 1466, 1454, 1370, 1337, 1315, 1179, 1159, 1052, 992, 816, 800, 752, 729, 722, 699, 631, 614 cm^−1^. ^1^H-NMR (600 MHz, CDCl_3_), δ = 7.31–7.27 (m, 3H), 7.26–7.20 (m, 2H), 7.15–7.11 (m, 5H), 7.10–7.05 (m, 4H), 7.04–7.01 (m, 1H), 6.91 (s, 1H), 6.84 (d, *J* = 8.4 Hz, 2H), 5.27 (d, *J* = 16.2 Hz, 1H), 5.23 (d, *J* = 16.2 Hz, 1H), 5.18 (s, 1H), 4.39–4.30 (m, 2H), 2.36 (s, 3H), 2.31 (s, 3H). ^13^C-NMR (150 MHz, CDCl_3_), δ = 181.2, 166.9, 139.2, 137.6, 137.1, 137.0, 135.6, 130.7, 130.3 (2C), 129.4 (2C), 128.9 (2C), 128.7 (2C), 128.1, 127.9 (2C), 127.8, 127.2, 126.8 (2C), 122.3, 119.7, 119.1, 113.3, 110.1, 59.1, 50.3, 42.5, 21.3, 21.2. MS (ESI): *m/z* = 484 [M + H]^+^; HRMS (TOF ES^+^): *m/z* [M + H]^+^ calcd for C_33_H_30_N_3_O^+^: 484.2383; found: 484.2385.

*2-((1-Benzyl-1H-indol-3-yl)(p-tolyl)methyl)-1-(4-methoxyphenyl)-1H-imidazol-5(4H)-one* (**3d**). Red solid; mp 200–202 °C; yield 207 mg (83%, method A); *R_f_* = 0.31 (EtOAc– hexane, 1:1). IR (KBr): 3022, 2902, 2838, 1730, 1628, 1513, 1465, 1373, 1338, 1302, 1255, 1174, 1161, 1051, 991, 831, 799, 750, 733, 700, 615, 583, 556 cm^−1^. ^1^H-NMR (600 MHz, CDCl_3_), δ = 7.32–7.27 (m, 3H), 7.26–7.21 (m, 2H), 7.16–7.11 (m, 3H), 7.10–7.05 (m, 4H), 7.04–7.01 (m, 1H), 6.91 (s, 1H), 6.86 (d, *J* = 9.0 Hz, 2H), 6.83 (d, *J* = 9.0 Hz, 2H), 5.28 (d, *J* = 16.2 Hz, 1H), 5.24 (d, *J* = 16.2 Hz, 1H), 5.16 (s, 1H), 4.39–4.30 (m, 2H), 3.80 (3H), 2.31 (s, 3H). ^13^C-NMR (150 MHz, CDCl_3_), δ = 181.4, 167.1, 160.0, 137.5, 137.1, 137.0, 135.5, 129.41 (2C), 129.40 (2C), 128.9 (2C), 128.7 (2C), 128.1, 127.8, 127.2, 126.8 (2C), 125.9, 122.3, 119.7, 119.1, 114.9 (2C), 113.3, 110.1, 59.1, 55.7, 50.3, 42.5, 21.2. MS (ESI): *m/z* = 500 [M + H]^+^; HRMS (TOF ES^+^): *m/z* [M + H]^+^ calcd for C_33_H_30_N_3_O_2_^+^: 500.2332; found: 500.2330.

*2-((1-Benzyl-1H-indol-3-yl)(p-tolyl)methyl)-1-(4-hydroxyphenyl)-1H-imidazol-5(4H)-one* (**3e**). Brownish solid; mp 164–166 °C; yield 163 mg (67%, method A); *R_f_* = 0.28 (EtOAc-hexane, 3:2). IR (KBr): 3280, 3030, 2926, 1736, 1629, 1611, 1514, 1467, 1453, 1377, 1336, 1273, 1170, 1054, 909, 834, 733, 698 cm^−1^. ^1^H-NMR (600 MHz, CDCl_3_), δ = 7.32–7.29 (m, 1H), 7.26–7.17 (m, 4H), 7.14–7.00 (m, 9H), 6.82 (s, 1H), 6.65 (d, *J* = 8.7 Hz, 2H), 6.50 (d, *J* = 8.7 Hz, 2H), 5.22–5.13 (m, 3H), 4.40–4.31 (m, 2H), 2.30 (s, 3H). ^13^C-NMR (150 MHz, CDCl_3_), δ = 182.1, 167.6, 157.2, 137.5, 137.2, 137.0, 135.2, 129.5 (2C), 129.3 (2C), 128.8 (2C), 128.7 (2C), 128.3, 127.7, 127.2, 126.8 (2C), 124.7, 122.3, 119.7, 119.0, 116.6 (2C), 113.1, 110.3, 59.0, 50.2, 42.6, 21.2. MS (ESI): *m/z* = 486 [M + H]^+^; HRMS (TOF ES^+^): *m/z* [M + H]^+^ calcd for C_32_H_28_N_3_O_2_^+^: 486.2176; found: 486.2178.

*2-((1-Benzyl-1H-indol-3-yl)(p-tolyl)methyl)-1-(4-chlorophenyl)-1H-imidazol-5(4H)-one* (**3f**). Light pink solid; mp 206–208 °C; yield 194 mg (77%, method A) and 167 mg (66%, method B); *R_f_* = 0.27 (EtOAc– hexane, 1:1). IR (KBr): 3024, 2909, 1898, 1737, 1631, 1553, 1512, 1493, 1466, 1454, 1378, 1336, 1313, 1173, 1156, 1093, 1051, 1018, 991, 962, 831, 794, 752, 723, 699, 624, 576, 564 cm^−1^. ^1^H-NMR (600 MHz, CDCl_3_), δ = 7.33–7.26 (m, 6H), 7.24 (d, *J* = 8.4 Hz, 1H), 7.18–7.14 (m, 1H), 7.12 (d, *J* = 8.2 Hz, 2H), 7.11–7.03 (m, 5H), 6.88–6.84 (m, 3H), 5.26 (d, *J* = 16.2 Hz, 1H), 5.22 (d, *J* = 16.2 Hz, 1H), 5.15 (s, 1H), 4.41–4.31 (m, 2H), 2.32 (s, 3H). ^13^C-NMR (150 MHz, CDCl_3_), δ = 180.8, 166.2, 137.4, 137.3, 137.0, 135.2, 135.1, 132.0, 129.8 (2C), 129.5 (2C), 129.4 (2C), 128.9 (2C), 128.7 (2C), 128.1, 127.8, 127.1, 126.8 (2C), 122.4, 119.9, 119.0, 112.8, 110.2, 59.1, 50.3, 42.8, 21.2. MS (ESI): *m/z* = 504 [M + H]^+^; HRMS (TOF ES^+^): *m/z* [M + H]^+^ calcd for C_32_H_27_ClN_3_O^+^: 504.1837; found: 504.1839.

*2-((1-Benzyl-1H-indol-3-yl)(p-tolyl)methyl)-1-(9H-fluoren-2-yl)-1H-imidazol-5(4H)-one* (**3g**). Brownish oil; yield 159 mg (57%, method A); *R_f_* = 0.41 (EtOAc– hexane, 1:1). IR (KBr): 3054, 2923, 2871, 2246, 1897, 1737, 1630, 1456, 1374, 1180, 1053, 1019, 909, 769, 734, 697, 643 cm^−1^. ^1^H-NMR (600 MHz, CDCl_3_), δ = 7.81 (d, *J* = 7.5 Hz, 1H), 7.75 (d, *J* = 7.9 Hz, 1H), 7.56 (d, *J* = 7.4 Hz, 1H), 7.44–7.41 (m, 1H), 7.38–7.35 (m, 1H), 7.31 (d, *J* = 7.9 Hz, 1H), 7.29–7.22 (m, 4H), 7.16–7.13 (m, 3H), 7.09 (d, *J* = 7.9 Hz, 2H), 7.07–7.05 (m, 2H), 7.04–7.00 (m, 3H), 6.96 (s, 1H), 5.28–5.20 (m, 3H), 4.47–4.35 (m, 2H), 3.83–3.68 (m, 2H), 2.33 (s, 3H). ^13^C-NMR (150 MHz, CDCl_3_), δ = 181.3, 167.0, 144.5, 143.7, 142.7, 140.7, 137.4, 137.1, 137.0, 135.5, 131.6, 129.4 (2C), 128.9 (2C), 128.8 (2C), 128.2, 127.8, 127.6, 127.3, 127.1, 126.83, 126.8 (2C), 125.3, 125.0, 122.3, 120.6, 120.4, 119.7, 119.1, 113.2, 110.1, 59.2, 50.3, 42.6, 36.9, 21.2. MS (ESI): *m/z* = 558 [M + H]^+^; HRMS (TOF ES^+^): *m/z* [M + H]^+^ calcd for C_39_H_32_N_3_O^+^: 558.2540; found: 558.2544.

*2-((1-Benzyl-1H-indol-3-yl)(4-chlorophenyl)methyl)-1-(p-tolyl)-1H-imidazol-5(4H)-one* (**3h**). Yellowish solid; mp 190–192 °C; yield 124 mg (49%, method A); *R_f_* = 0.50 (EtOAc– hexane, 1:1). IR (KBr): 3062, 3029, 2914, 1890, 1732, 1630, 1615, 1513, 1489, 1474, 1455, 1370, 1328, 1192, 1178, 1158, 1089, 1049, 1015, 986, 909, 752, 738, 724, 699 cm^−1^. ^1^H-NMR (600 MHz, CDCl_3_), δ = 7.32–7.26 (m, 4H), 7.25–7.23 (m, 3H), 7.18 (d, *J* = 8.4 Hz, 2H), 7.16–7.11 (m, 3H), 7.09–7.05 (m, 2H), 7.03 (ddd, *J* = 7.9, 7.1, 0.9 Hz, 1H), 6.91 (s, 1H), 6.84 (d, *J* = 8.3 Hz, 2H), 5.28 (d, *J* = 16.2 Hz, 1H), 5.24 (d, *J* = 16.2 Hz, 1H), 5.20 (s, 1H), 4.40–4.30 (m, 2H), 2.36 (s, 3H). ^13^C-NMR (150 MHz, CDCl_3_), δ = 180.9, 166.5, 139.4, 137.4, 137.2, 137.0, 133.4, 130.5, 130.4 (2C), 130.2 (2C), 128.9 (2C), 128.8 (2C), 128.0, 127.9, 127.8 (2C), 127.0, 126.8 (2C), 122.5, 119.9, 119.0, 112.6, 110.2, 59.1, 50.3, 42.3, 21.3. MS (ESI): *m/z* = 504 [M + H]^+^; HRMS (TOF ES^+^): *m/z* [M + H]^+^ calcd for C_32_H_27_ClN_3_O^+^: 504.1837; found: 504.1840.

*2-((1-Benzyl-1H-indol-3-yl)(4-chlorophenyl)methyl)-1-(4-methoxyphenyl)-1H-imidazol-5(4H)-one* (**3i**). Brownish oil; yield 130 mg (50%, method A); *R_f_* = 0.35 (EtOAc– hexane, 1:1). IR (KBr): 3061, 2933, 2837, 2048, 1889, 1737, 1630, 1512, 1490, 1466, 1334, 1299, 1249, 1174, 1089, 1029, 831, 803, 787, 735, 698 cm^−1^. ^1^H-NMR (600 MHz, CDCl_3_), δ = 7.32–7.27 (m, 4H), 7.26–7.23 (m, 3H), 7.18 (d, *J* = 8.5 Hz, 2H), 7.17–7.14 (m, 1H), 7.09–7.05 (m, 2H), 7.03 (ddd, *J =* 7.9, 7.1, 0.8 Hz 1H), 6.91 (s, 1H), 6.85 (d, *J* = 9.1 Hz, 2H), 6.82 (d, *J* = 9.1 Hz, 2H), 5.28 (d, *J* = 16.2 Hz, 1H), 5.24 (d, *J* = 16.2 Hz, 1H), 5.18 (s, 1H), 4.40–4.29 (m, 2H), 3.80 (s, 3H). ^13^C-NMR (150 MHz, CDCl_3_), δ = 181.1, 166.6, 160.1, 137.4, 137.2, 137.0, 133.4, 130.2 (2C), 129.3 (2C), 128.9 (2C), 128.8 (2C), 128.0, 127.9, 127.0, 126.8 (2C), 125.7, 122.5, 119.9, 119.0, 115.0 (2C), 112.5, 110.2, 59.0, 55.7, 50.3, 42.3. MS (ESI): *m/z* = 520 [M + H]^+^; HRMS (TOF ES^+^): *m/z* [M + H]^+^ calcd for C_32_H_27_ClN_3_O_2_^+^: 520.1786; found: 520.1790.

*2-((1-Benzyl-5-bromo-1H-indol-3-yl)(p-tolyl)methyl)-1-(4-methoxyphenyl)-1H-imidazol-5(4H)-one* (**3j**). Orange oil; yield 240 mg (83%, method A); *R_f_* = 0.29 (EtOAc– hexane, 1:1). IR (KBr): 3031, 2938, 2917, 2837, 1869, 1735, 1686, 1630, 1542, 1511, 1467, 1374, 1333, 1299, 1249, 1173, 1107, 1051, 1030, 993, 830, 791, 729, 698, 619 cm^−1^. ^1^H-NMR (600 MHz, CDCl_3_), δ = 7.38 (d, *J* = 1.8 Hz, 1H), 7.31–7.26 (m, 3H), 7.20 (dd, *J* = 9.0, 1.8 Hz, 1H), 7.12–7.08 (m, 4H), 7.07 (d, *J* = 8.4 Hz, 1H), 7.05–7.01 (m, 2H), 6.96 (s, 1H), 6.86–6.83 (m, 4H), 5.24 (d, *J* = 16.2 Hz, 1H), 5.21 (d, *J* = 16.2 Hz, 1H), 5.09 (s, 1H), 4.39–4.29 (m, 2H), 3.82 (s, 3H), 2.32 (s, 3H). ^13^C-NMR (150 MHz, CDCl_3_), δ = 181.2, 166.9, 160.1, 137.3, 137.0, 135.5, 135.1, 129.5 (2C), 129.4 (2C), 129.3, 129.0 (2C), 128.9, 128.6 (2C), 128.0, 126.7 (2C), 125.7, 125.2, 121.6, 114.9 (2C), 113.1, 112.9, 111.7, 59.0, 55.7, 50.5, 42.3, 21.2. MS (ESI): *m/z* = 578 [M + H]^+^; HRMS (TOF ES^+^): *m/z* [M + H]^+^ calcd for C_33_H_29_BrN_3_O_2_^+^: 578.1438; found: 578.1450.

*2-((1-Benzyl-5-bromo-1H-indol-3-yl)(p-tolyl)methyl)-1-(4-hydroxyphenyl)-1H-imidazol-5(4H)-one* (**3k**). Brownish solid; mp 126–128 °C; yield 203 mg (72%, method A); *R_f_* = 0.22 (EtOAc– hexane, 1:1). IR (KBr): 3270, 3062, 3033, 2921, 1735, 1628, 1542, 1514, 1468, 1454, 1376, 1338, 1274, 1169, 1053, 908, 834, 791, 730, 689 cm^−1^. ^1^H-NMR (600 MHz, CDCl_3_), δ = 7.41 (d, *J* = 1.8 Hz, 1H), 7.26–7.22 (m, 4H), 7.19 (dd, *J* = 8.7, 1.8 Hz, 1H), 7.09–7.02 (m, 5H), 7.01–6.97 (m, 2H), 6.84 (s, 1H), 6.65 (d, *J* = 8.7 Hz, 2H), 6.52 (d, *J* = 8.7 Hz, 2H), 5.17 (d, *J* = 16.2 Hz, 1H), 5.12 (d, *J* = 16.2 Hz, 1H), 5.06 (s, 1H), 4.41–4.32 (m, 2H), 2.31 (s, 3H). ^13^C-NMR (150 MHz, CDCl_3_), δ = 181.9, 167.3, 157.1, 137.5, 137.0, 135.6, 134.7, 129.6 (2C), 129.5, 129.3 (2C), 128.9 (2C), 128.8, 128.6 (2C), 127.9, 126.7 (2C), 125.2, 124.7, 121.5, 116.6 (2C), 113.2, 112.7, 111.8, 59.0, 50.4, 42.4, 21.2. MS (ESI): *m/z* = 564 [M + H]^+^; HRMS (TOF ES^+^): *m/z* [M + H]^+^ calcd for C_32_H_27_N_3_O_2_^+^: 564.1281; found: 564.1284.

*2-((1-Benzyl-5-bromo-1H-indol-3-yl)(p-tolyl)methyl)-1-(4-chlorophenyl)-1H-imidazol-5(4H)-one* (**3l**). Brownish oil; yield 198 mg (68%, method A); *R_f_* = 0.38 (EtOAc– hexane, 1:1). IR (KBr): 3063, 3031, 2919, 2867, 2246, 1896, 1736, 1631, 1509, 1492, 1468, 1370, 1332, 1172, 1091, 1052, 1017, 993, 908, 827, 791, 730, 698, 648 cm^−1^. ^1^H-NMR (600 MHz, CDCl_3_), δ = 7.42 (d, *J* = 1.9 Hz, 1H), 7.32–7.26 (m, 5H), 7.22 (dd, *J* = 8.7, 1.9 Hz, 1H), 7.12–7.06 (m, 5H), 7.04–7.01 (m, 2H), 6.90 (s, 1H), 6.86 (d, *J* = 8.7 Hz, 2H), 5.23 (d, *J* = 16.2 Hz, 1H), 5.19 (d, *J* = 16.2 Hz, 1H), 5.07 (s, 1H), 4.41–4.31 (m, 2H), 2.32 (s, 3H). ^13^C-NMR (150 MHz, CDCl_3_), δ = 180.6, 165.9, 137.5, 136.9, 135.6, 135.3, 134.7, 131.8, 129.9 (2C), 129.6 (2C), 129.5 (2C), 129.3, 129.0 (2C), 128.7, 128.6 (2C), 128.0, 126.7 (2C), 125.3, 121.6, 113.3, 112.6, 111.8, 59.0, 50.5, 42.6, 21.2. MS (ESI): *m/z* = 582 [M + H]^+^; HRMS (TOF ES^+^): *m/z* [M + H]^+^ calcd for C_32_H_26_BrClN_3_O^+^: 582.0942; found: 582.0951.

*2-((1-Benzyl-5-methoxy-1H-indol-3-yl)(4-chlorophenyl)methyl)-1-(4-methoxyphenyl)-1H-imidazol-5(4H)-one* (**3m**). Brownish oil; yield 140 mg (51%, method A); *R_f_* = 0.31 (EtOAc– hexane, 1:1). IR (KBr): 3064, 3001, 2933, 2835, 1736, 1685, 1628, 1511, 1489, 1453, 1375, 1333, 1299, 1249, 1174, 1090, 1031, 913, 830, 793, 733, 704 cm^−1^. ^1^H-NMR (600 MHz, CDCl_3_), δ = 7.31–7.27 (m, 3H), 7.25–7.23 (m, 2H), 7.20 (d, *J* = 8.5 Hz, 2H), 7.12 (d, *J* = 8.9 Hz, 1H), 7.08–7.05 (m, 2H), 6.90 (s, 1H), 6.85 (d, *J* = 8.9 Hz, 2H), 6.83–6.79 (m, 3H), 6.70 (d, *J* = 2.4 Hz, 1H), 5.24 (d, *J* = 16.2 Hz, 1H), 5.21 (d, *J* = 16.2 Hz, 1H), 5.15 (s, 1H), 4.38–4.30 (m, 2H), 3.80 (s, 3H), 3.74 (s, 3H). ^13^C-NMR (150 MHz, CDCl_3_), δ = 181.1, 166.7, 160.1, 154.3, 137.4, 137.2, 133.4, 132.3, 130.2 (2C), 129.3 (2C), 128.9 (2C), 128.8 (2C), 128.6, 127.9, 127.5, 126.8 (2C), 125.6, 114.9 (2C), 112.3, 111.6, 111.0, 101.1, 59.0, 55.9, 55.7, 50.5, 42.3. MS (ESI): *m/z* = 550 [M + H]^+^; HRMS (TOF ES^+^): *m/z* [M + H]^+^ calcd for C_33_H_29_ClN_3_O_3_^+^: 550.1892; found: 550.1895.

*2-((1-Benzyl-5-methoxy-1H-indol-3-yl)(4-chlorophenyl)methyl)-1-(p-tolyl)-1H-imidazol-5(4H)-one* (**3n**). Brownish oil; yield 128 mg (48%, method A); *R_f_* = 0.44 (EtOAc– hexane, 1:1). IR (KBr): 3064, 3032, 2920, 2834, 1896, 1737, 1683, 1629, 1577, 1514, 1489, 1454, 1373, 1332, 1214, 1175, 1090, 1039, 1015, 910, 816, 797, 730, 704, 613 cm^−1^. ^1^H-NMR (600 MHz, CDCl_3_), δ = 7.32–7.27 (m, 3H), 7.26–7.23 (m, 2H), 7.19 (d, *J* = 8.5 Hz, 2H), 7.14–7.10 (m, 3H), 7.07–7.04 (m, 2H), 6.87 (s, 1H), 6.85–6.79 (m, 3H), 6.68 (d, *J* = 2.4 Hz, 1H), 5.24 (d, *J* = 16.2 Hz, 1H), 5.20 (d, *J* = 16.2 Hz, 1H), 5.14 (s, 1H), 4.40–4.28 (m, 2H), 3.73 (s, 3H), 2.36 (s, 3H). ^13^C-NMR (150 MHz, CDCl_3_), δ = 180.9, 166.6, 154.3, 139.4, 137.4, 137.2, 133.4, 132.3, 130.6, 130.3 (2C), 130.2 (2C), 128.9 (2C), 128.8 (2C), 128.7, 127.90, 127.8 (2C), 127.5, 126.8 (2C), 112.3, 111.7, 111.0, 101.2, 59.1, 56.0, 50.5, 42.3, 21.3. MS (ESI): *m/z* = 534 [M + H]^+^; HRMS (TOF ES^+^): *m/z* [M + H]^+^ calcd for C_33_H_29_ClN_3_O_2_^+^: 534.1942; found: 534.1947.

*2-((1-Benzyl-5-methoxy-1H-indol-3-yl)(4-chlorophenyl)methyl)-1-(4-hydroxyphenyl)-1H-imidazol-5(4H)-one* (**3o**). Brownish solid; mp 102–104 °C; yield 129 mg (40%, method A); *R_f_* = 0.32 (EtOAc– hexane, 1:1). IR (KBr): 3250, 3064, 3033, 2963, 2951, 1734, 1717, 1684, 1624, 1514, 1489, 1456, 1339, 1271, 1218, 1173, 1090, 1014, 834, 735 cm^−1^. ^1^H-NMR (600 MHz, CDCl_3_), δ = 7.29–7.27 (m, 2H), 7.26–7.22 (m, 4H), 7.16 (d, *J* = 8.4 Hz, 2H), 7.10 (d, *J* = 9.1 Hz, 1H), 7.04–7.01 (m, 2H), 6.82–6.78 (m, 2H), 6.70 (d, *J* = 2.4 Hz, 1H), 6.64 (d, *J* = 8.8 Hz, 2H), 6.52 (d, *J* = 8.8 Hz, 2H), 5.18 (d, *J* = 16.2 Hz, 1H), 5.14 (d, *J* = 16.2 Hz, 1H), 5.09 (s, 1H), 4.39–4.34 (m, 2H), 3.74 (s, 3H). ^13^C-NMR (150 MHz, CDCl_3_), δ = 181.9, 167.1, 157.1, 154.2, 137.4, 136.8, 133.5, 132.3, 130.2 (2C), 129.3 (2C), 128.9 (2C), 128.8, 127.8 (2C), 127.4, 126.8 (2C), 124.6, 116.6 (2C), 115.9, 112.2, 111.4, 111.1, 101.2, 59.0, 56.0, 50.4, 42.3. MS (ESI): *m/z* = 536 [M + H]^+^; HRMS (TOF ES^+^): *m/z* [M + H]^+^ calcd for C_32_H_27_ClN_3_O_3_^+^: 536.1735; found: 536.1736.

*1-Benzyl-2-((1-benzyl-1H-indol-3-yl)(4-methoxyphenyl)methyl)-1H-imidazol-5(4H)-one* (**3p**) Brownish oil; yield 63 mg (25%, method B); *R_f_* = 0.17 (EtOAc– hexane, 1:1). IR (KBr): 3060, 3030, 2926, 2836, 1885, 1727, 1678, 1627, 1510, 1466, 1384, 1334, 1249, 1173, 1029, 805, 741, 698 cm^−1^. ^1^H-NMR (600 MHz, CDCl_3_), δ = 7.41–7.33 (m, 3H), 7.30–7.26 (m, 2H), 7.26–7.21 (m, 2H), 7.21–7.18 (m, 2H), 7.15 (ddd, *J* = 8.2, 7.0, 1.1 Hz, 1H), 7.13–7.09 (m, 3H), 7.07–7.04 (m, 2H), 7.02 (ddd, *J* = 8.0, 7.0, 1.0 Hz, 1H), 6.84 (d, *J* = 8.8 Hz, 2H), 6.83 (s, 1H), 5.29 (d, *J* = 16.2 Hz, 1H), 5.21 (d, *J* = 16.2 Hz, 1H), 5.18 (s, 1H), 4.58 (s, 2H), 4.32–4.29 (m, 2H), 3.77 (s, 3H). ^13^C-NMR (150 MHz, CDCl_3_), δ = 181.3, 167.5, 159.1, 137.4, 137.1, 136.5, 134.6, 129.8 (2C), 129.2 (2C), 128.9 (2C), 128.2, 127.9, 127.8, 127.1, 127.0 (2C), 126.8 (2C), 122.5, 119.9, 118.9, 114.3 (2C), 112.8, 110.3, 58.6, 55.4, 50.3, 43.7, 42.4. MS (ESI): *m/z* = 500 [M + H]^+^; HRMS (TOF ES^+^): *m/z* [M + H]^+^ calcd for C_33_H_30_N_3_O_2_^+^: 500.2333; found: 500.2334.

*2-((1-Benzyl-1H-indol-3-yl)(4-methoxyphenyl)methyl)-1-isopropyl-1H-imidazol-5(4H)-one* (**3q**) Brownish oil; yield 77 mg (34%, method B); *R_f_* = 0.43 (EtOAc– hexane, 1:1). IR (KBr): 3056, 3029, 2925, 2854, 1885, 1722, 1613, 1511, 1466, 1364, 1302, 1249, 1176, 1121, 1033, 966, 797, 740, 698, 621 cm^−1^. ^1^H-NMR (600 MHz, CDCl_3_), δ = 7.43 (d, *J* = 8.0 Hz, 1H), 7.30–7.26 (m, 4H), 7.26–7.23 (m, 2H), 7.19 (ddd, *J* = 8.2, 7.1, 1.1 Hz, 1H), 7.10–7.07 (m, 3H), 6.91 (s, 1H), 6.88 (d, *J* = 8.8 Hz, 2H), 5.42 (s, 1H), 5.32–5.26 (m, 2H), 4.11–4.09 (m, 2H), 4.02–3.96 (m, 1H), 3.79 (s, 3H), 1.25 (d, *J* = 6.9 Hz, 3H), 1.22 (d, *J* = 6.8 Hz, 3H). ^13^C-NMR (150 MHz, CDCl_3_), δ = 181.9, 167.7, 159.0, 137.4, 137.2, 130.2, 129.9 (2C), 128.9 (2C), 127.8, 127.6, 127.1, 126.8 (2C), 122.6, 119.9, 119.1, 114.3 (2C), 113.0, 110.3, 58.8, 55.4, 50.3, 46.8, 42.8, 19.7, 19.6. MS (ESI): *m/z* = 452 [M + H]^+^; HRMS (TOF ES^+^): *m/z* [M + H]^+^ calcd for C_29_H_30_N_3_O_2_^+^: 452.2332; found: 492.2327.

*2-((1-Benzyl-1H-indol-3-yl)(4-methoxyphenyl)methyl)-1-cyclohexyl-1H-imidazol-5(4H)-one* (**3r**) Brownish oil; yield 85 mg (35%, method B); *R_f_* = 0.31 (EtOAc– hexane, 1:1). IR (KBr): 3114, 3068, 3032, 2954, 2932, 2854, 1875, 1724, 1623, 1548, 1513, 1468, 1451, 1304, 1243, 1198, 1176, 1051, 1015, 974, 893, 854, 816, 795, 738, 699, 569 cm^−1^. ^1^H-NMR (600 MHz, CDCl_3_), δ = 7.43 (d, *J* = 8.0 Hz, 1H), 7.30–7.26 (m, 5H), 7.26–7.22 (m, 1H), 7.21–7.17 (m, 1H), 7.11–7.07 (m, 3H), 6.82 (s, 1H), 6.88 (d, *J* = 8.7 Hz, 2H), 5.45 (s, 1H), 5.32–5.26 (m, 2H), 4.13–4.10 (m, 2H), 3.79 (s, 3H), 3.57–3.50 (m, 1H), 2.16–2.06 (m, 2H), 1.72–1.66 (m, 1H), 1.65–1.58 (m, 1H), 1.54–1.47 (m, 1H), 1.36–1.30 (m, 1H), 1.24–1.17 (m, 1H), 1.14–1.05 (m, 1H), 1.04–0.94 (m, 1H), 0.86–0.76 (m, 1H). ^13^C-NMR (150 MHz, CDCl_3_), δ = 181.6, 167.6, 159.0, 137.4, 137.1, 130.1, 129.9 (2C), 128.9 (2C), 127.8, 127.6, 127.1, 126.8 (2C), 122.6, 120.0, 119.1, 114.3 (2C), 113.2, 110.3, 58.6, 55.4, 55.1, 50.3, 42.8, 29.3, 29.1, 26.2, 26.1, 24.9. MS (ESI): *m/z* = 492 [M + H]^+^; HRMS (TOF ES^+^): *m/z* [M + H]^+^ calcd for C_32_H_34_N_3_O_2_^+^: 492.2646; found: 492.2649.

*1-Benzyl-2-((1-benzyl-1H-indol-3-yl)(p-tolyl)methyl)-1H-imidazol-5(4H)-one* (**3s**) Brownish oil; yield 64 mg (26%, method B); *R_f_* = 0.44 (EtOAc– hexane, 1:1). IR (KBr): 3058, 3029, 2920, 2850, 1727, 1690, 1680, 1629, 1549, 1467, 1453, 1383, 1334, 1170, 1014, 910, 796, 730, 698 cm^−1^. ^1^H-NMR (600 MHz, CDCl_3_), δ = 7.40–7.35 (m, 3H), 7.29–7.26 (m, 2H), 7.25–7.18 (m, 4H), 7.14 (ddd, *J* = 8.2, 7.0, 1.1 Hz, 1H), 7.13–7.10 (m, 3H), 7.08 (d, *J* = 8.2 Hz, 2H), 7.06–7.03 (m, 2H), 7.01 (ddd, *J* = 8.0, 7.0, 1.0 Hz, 1H), 6.82 (s, 1H), 5.28 (d, *J* = 16.2 Hz, 1H), 5.20 (d, *J* = 16.2 Hz, 1H), 5.19 (s, 1H), 4.58 (d, *J* = 16.2 Hz, 1H), 4.54 (d, *J* = 16.2 Hz, 1H), 4.29–4.27 (m, 2H), 2.31 (s, 3H). ^13^C-NMR (150 MHz, CDCl_3_), δ = 180.5, 167.3, 137.4, 137.3, 137.1, 136.5, 134.8, 129.7 (2C), 129.2 (2C), 128.9 (2C), 128.6 (2C), 128.2, 127.9, 127.8, 127.1, 127.0 (2C), 126.7 (2C), 122.5, 119.9, 119.0, 113.3, 110.3, 58.7, 50.3, 43.7, 42.9, 21.2. MS (ESI): *m/z* = 484 [M + H]^+^; HRMS (TOF ES^+^): *m/z* [M + H]^+^ calcd for C_33_H_30_BrClN_3_O^+^: 484.2383; found: 484.2384.

*Ethyl-2-(2-(1-benzyl-1H-indol-3-yl)-N’-(2-chlorophenyl)-2-(p-tolyl)acetimidamido)acetate* (**10a**). Light orange oil; yield 201 mg (73%, method A); *R_f_* = 0.53 (EtOAc-hexane, 1:4). IR (KBr): 3428, 2978, 2924, 1741, 1634, 1509, 1468, 1194, 1030, 799, 739 cm^−1^. ^1^H-NMR (600 MHz, CDCl_3_), δ = 7.39 (d, *J* = 8.0 Hz, 1H), 7.33–7.28 (m, 3H), 7.27–7.22 (m, 2H), 7.16–7.12 (m, 3H), 7.11–7.06 (m, 4H), 7.03 (ddd, *J* = 8.0, 5.4, 0.9 Hz, 1H), 6.99 (s, 1H), 6.87 (td, *J* = 7.6, 1.5 Hz, 1H), 6.83 (td, *J* = 7.6, 1.7 Hz, 1H), 6.43 (dd, *J* = 7.7, 1.5 Hz, 1H), 5.33–5.25 (m, 3H), 5.21 (s, 1H), 4.22–4.11 (m, 2H), 4.18 (q, *J* = 7.2 Hz, 2H), 2.34 (s, 3H), 1.25 (t, *J* = 7.2 Hz, 3H). ^13^C-NMR (150 MHz, CDCl_3_), δ = 170.8, 158.2, 147.4, 137.6, 137.1, 136.8, 136.7, 129.5, 129.4 (2C), 129.1, 128.9 (2C), 128.7 (2C), 127.7, 127.3, 126.8, 126.6 (2C), 126.5, 124.1, 123.0, 122.3, 119.9, 119.7, 114.2, 110.0, 61.2, 50.3, 44.8, 43.5, 21.3, 14.3. MS (ESI): *m/z* = 550 [M + H]^+^; HRMS (TOF ES^+^): *m/z* [M + H]^+^ calcd for C_34_H_33_ClN_3_O_2_^+^: 550.2256; found: 550.2258.

*Ethyl-2-(2-(1-benzyl-1H-indol-3-yl)-N’-(4-bromo-2-chlorophenyl)-2-phenylacetimidamido)acetate* (**10b**). Orange oil; yield 230 mg (75%, method A); *R_f_* = 0.47 (EtOAc-hexane, 1:3). IR (KBr): 3429, 3063, 3030, 2979, 2928, 2160, 1951, 1885, 1742, 1631, 1511, 1466, 1375, 1196, 1029, 864, 818, 740 cm^−1^. ^1^H-NMR (600 MHz, CDCl_3_), δ = 7.46 (d, *J* = 2.2 Hz, 1H), 7.42 (d, *J* = 8.0 Hz, 1H), 7.32–7.27 (m, 5H), 7.26–7.22 (m, 4H), 7.17 (ddd, *J* = 8.2, 7.1, 1.1 Hz, 1H), 7.10–7.04 (m, 3H), 6.95 (dd, *J* = 8.5, 2.2 Hz, 1H), 6.90 (s, 1H), 6.24 (d, *J* = 8.5 Hz, 1H), 5.38 (br s, 1H), 5.30 (d, *J* = 16.2 Hz, 1H), 5.26 (d, *J* = 16.2 Hz, 1H), 5.19 (s, 1H), 4.22-4.07 (m, 2H), 4.18 (q, *J* = 7.2 Hz, 2H), 1.24 (t, *J* = 7.2 Hz, 3H). ^13^C-NMR (150 MHz, CDCl_3_), δ = 170.5, 158.4, 146.7, 139.5, 137.5, 137.1, 131.9, 129.8, 129.2, 129.0 (2C), 128.9 (2C), 128.8 (2C), 127.8, 127.5, 127.4, 127.2, 126.7 (2C), 125.3, 122.6, 119.9, 119.8, 114.4, 113.8, 110.2, 61.3, 50.3, 45.4, 43.5, 14.3. MS (ESI): *m/z* = 614 [M + H]^+^; HRMS (TOF ES^+^): *m/z* [M + H]^+^ calcd for C_33_H_30_BrClN_3_O^+^: 614.1204; found: 614.1208.

*Methyl-4-((2-(1-benzyl-1H-indol-3-yl)-1-((2-ethoxy-2-oxoethyl)amino)-2-(p-tolyl) ethylidene) amino)benzoate* (**10c**). Brownish oil; yield 189 mg (66%, method A); *R_f_* = 0.47 (EtOAc-hexane, 1:3). IR (KBr): 3419, 3056, 3027, 2971, 2930, 1752, 1705, 1634, 1594, 1519, 1497, 1467, 1435, 1281, 1255, 1190, 1167, 1112, 1101, 1015, 964, 916, 875, 804, 777, 730, 711, 696, 600 cm^−1^. ^1^H-NMR (600 MHz, CDCl_3_), δ = 7.83 (d, *J* = 8.5 Hz, 2H), 7.32–7.28 (m, 3H), 7.27–7.21 (m, 2H), 7.17 (ddd, *J* = 8.0, 7.1, 1.0 Hz, 1H), 7.14–7.09 (m, 4H), 7.08–7.04 (m, 3H), 6.93 (s, 1H), 6.71 (d, *J* = 5.5 Hz, 2H), 5.33 (s, 1H), 5.30 (d, *J* = 16.2 Hz, 1H), 5.26 (d, *J* = 16.2 Hz, 1H), 4.17 (q, *J* = 7.2 Hz, 2H), 4.14–4.00 (m, 2H), 3.86 (s, 3H), 2.34 (s, 3H), 1.24 (t, *J* = 7.2 Hz, 3H). ^13^C-NMR (150 MHz, CDCl_3_), δ = 170.8, 167.4, 153.1, 152.0, 137.5, 137.2, 137.0, 131.7, 130.6 (2C), 129.6 (2C), 128.9 (2C), 128.5 (2C), 127.8 (2C), 127.2, 126.6(2C), 124.0, 122.6, 122.3, 122.2, 119.9, 119.4, 113.9, 110.2, 61.3, 51.9, 50.3, 44.2, 43.5, 21.3, 14.3. MS (ESI): *m/z* = 574 [M + H]^+^; HRMS (TOF ES^+^): *m/z* [M + H]^+^ calcd for C_36_H_36_N_3_O_4_^+^: 574.2700; found: 574.2703.

*Ethyl-2-(2-(1-benzyl-1H-indol-3-yl)-N’-(tert-butyl)-2-(4-methoxyphenyl)acetimidamido)acetate* (**10d**) Brownish oil; yield 100 mg (43%, method A); *Rf* = 0.40 (EtOAc– hexane, 1:1). IR (KBr): 3412, 3059, 3030, 2957, 2932, 2836, 1738, 1638, 1611, 1583, 1510, 1466, 1453, 1357, 1249, 1177, 1110, 1034, 820, 808, 742, 696 cm^−1^. ^1^H-NMR (600 MHz, CDCl_3_), δ = 7.51 (d, *J* = 8.0 Hz, 1H), 7.29–7.21 (m, 6H), 7.20–7.06 (m, 2H), 7.02–6.96 (m, 2H), 6.87 (d, *J* = 8.8 Hz, 2H), 6.64 (s, 1H), 5.35 (s, 1H), 5.29 (d, *J* = 16.2 Hz, 1H), 5.23 (d, *J* = 16.2 Hz, 1H), 4.24–4.00 (m, 5H), 3.81 (s, 3H), 1.26 (s, 12H). ^13^C-NMR (150 MHz, CDCl3), δ = 173.2, 158.9, 158.6, 137.7, 137.1, 131.8, 129.8 (2C), 128.9 (2C), 128.5, 127.7, 127.5, 126.4 (2C), 122.5, 119.9, 119.8, 115.4, 114.3 (2C), 110.0, 60.4, 57.8, 55.4, 51.1, 50.1, 43.4, 28.6 (3C), 14.4. MS (ESI): *m/z* = 512 [M + H]^+^; HRMS (TOF ES^+^): *m/z* [M + H]^+^ calcd for C_32_H_38_N_3_O_3_^+^: 512.2908; found: 512.2911.

*Ethyl-2-((2-(1-benzyl-1H-indol-3-yl)-1-((benzyloxy)amino)-2-(4-methoxyphenyl)ethylidene)amino)acetate* (**10e**). Orange oil; yield 205 mg (73%, method A); *R_f_* = 0.67 (EtOAc-hexane, 1:3). IR (KBr): 3403, 3059, 3030, 2979, 2931, 2836, 1743, 1632, 1611, 1510, 1496, 1466, 1453, 1370, 1247, 1201, 1177, 1028, 820, 741, 697cm^−1^. ^1^H-NMR (600 MHz, CDCl_3_, mixture of tautomers 1:1), δ = 7.42 (d, *J* = 8.0 Hz, 1H), 7.39–7.36 (m, 2H), 7.33–7.26 (m, 12H), 7.26–7.19 (m, 9H), 7.17–7.12 (m, 2H), 7.09–7.05 (m, 4H), 7.04–7.00 (m, 2H), 6.87 (dd*, J* = 8.6 Hz, 2H), 6.84–6.80 (m, 4H), 6.02 (s, 1H), 5.72 (br s, 1H), 5.29–5.22 (m, 4H), 5.17 (s, 1H), 5.02–4.97 (m, 2H), 4.92–4.83 (m, 2H), 4.22 (br s, 1H), 4.16–4.02 (m, 4H), 3.90–3.75 (m, 4H), 3.81 (s, 3H), 3.79 (s, 3H), 1.20 (t, *J* = 7.2 Hz, 3H), 1.18 (t, *J* = 7.2 Hz, 3H). ^13^C-NMR (150 MHz, CDCl_3_, mixture of tautomers 1:1), δ = 170.8, 170.5, 158.5, 158.4, 158.1, 154.5, 138.9, 138.8, 137.8, 137.6, 137.0, 136.9, 131.9, 131.4, 129.9 (2C), 129.8 (2C), 128.9 (2C), 128.8 (2C), 128.7, 128.5 (2C), 128.4 (2C), 128.3 (2C), 128.1 (2C), 128.0, 127.7, 127.6, 127.59, 127.57, 127.4, 126.6 (2C), 122.3, 122.1, 120.0, 119.7, 119.6, 119.5, 114.3, 114.03 (2C), 113.98, 113.8 (2C), 110.0, 109.9, 75.6, 75.3, 61.4, 61.0, 55.4, 55.3, 50.2, 50.1, 44.7, 43.9, 42.8, 40.2, 14.24, 14.18. MS (ESI): *m/z* = 562 [M − H]^+^; HRMS (TOF ES^+^): *m/z* [M − H]^+^ calcd for C_35_H_36_N_3_O_4_^+^: 562.2711; found: 562.2706.

*Ethyl-2-(2-(1-benzyl-1H-indol-3-yl)-N’-(benzyloxy)-2-(p-tolyl)acetimidamido)acetate* (**10f**). Orange oil; yield 109 mg (60%, method A); *R_f_* = 0.44 (EtOAc-hexane, 1:5). IR (KBr): 3405, 3028, 2979, 2919, 2861, 1742, 1631, 1511, 1496, 1467, 1453, 1371, 1334, 1200, 1024, 910, 807, 777, 740, 697 cm^−1^. ^1^H-NMR (600 MHz, CDCl_3_, mixture of tautomers 1:1), δ = 7.42 (d, *J* = 8.0 Hz, 1H), 7.40–7.37 (m, 2H), 7.33–7.26 (m, 11H), 7.26–7.18 (m, 10H), 7.17–7.12 (m, 4H), 7.09 (d, *J* = 8.0 Hz, 2H), 7.08–7.05 (m, 4H), 7.02 (ddd, *J* = 8.0, 7.0, 1.0 Hz, 2H), 6.83 (dd*, J* = 2.9, 0.7 Hz, 2H), 6.04 (s, 1H), 5.73 (br s, 1H), 5.29–5.23 (m, 4H), 5.20 (s, 1H), 5.00 (m, 2H), 4.91–482 (m, 2H), 4.23 (br s, 1H), 4.11–4.00 (m, 4H), 3.91–3.75 (m, 4H), 2.36 (s, 3H), 2.33 (s, 3H), 1.20 (t, *J* = 7.2 Hz, 3H), 1.18 (t, *J* = 7.2 Hz, 3H). ^13^C-NMR (150 MHz, CDCl_3_, mixture of tautomers 1:1), δ = 170.8, 170.5, 158.1, 154.4, 138.9, 138.8, 137.8, 137.6, 137.0, 136.9, 136.7, 136.4, 136.3, 136.2, 129.4 (2C), 129.2 (2C), 128.9 (2C), 128.8 (2C), 128.7 (2C), 128.6, 128.5 (2C), 128.3 (2C), 128.1 (2C), 128.0, 127.8, 127.7. 127.6, 127.5 (2C), 127.4, 127.1, 126.8, 126.6 (2C), 126.5 (2C), 122.3, 122.1, 120.0, 119.7, 119.6, 119.5, 114.1, 113.8, 110.0, 109.8, 75.6, 75.3, 61.3, 61.0, 50.2, 50.1, 44.7, 43.9, 43.2, 40.6, 21.3, 21.2, 14.3, 14.2. MS (ESI): *m/z* = 546 [M − H]^+^; HRMS (TOF ES^+^): *m/z* [M − H]^+^ calcd for C_35_H_36_N_3_O_3_^+^: 546.2762; found: 546.2756.

## 4. Conclusions

We have developed a new three-component reaction of 3-arylidene-3*H*-indolium salt, isocyanide and amine leading to *N*,*N*-disubstituted aryl(indol-3-yl)acetimidamides with yields up to 96%. We have also shown that in the case of ethyl isocyanoacetate the cyclization to form 3,5-dihydro-4*H*-imidazol-4-one (1*H*-imidazol-5(4*H*)-one) fragment could take place. These reactions furnish a new practical synthetic approach to a series of compounds with a privileged indole scaffold, which are prospective choices for seeking new physiologically active compounds.

## Figures and Tables

**Table 1:** Reaction optimization for imidamide 2a synthesis.

Entry	Solvent	Temperature (°C)	Time (h)	Yield (%) ^a^
1 ^b^	MeOH	20 °C	12 h	53 (10)
2 ^b^	EtOH	20 °C	12 h	64 (5)
3 ^b^	Toluene	20 °C	12 h	85 (3)
4 ^b^	1,4-Dioxane	20 °C	12 h	71 (8)
5 ^b^	DMF	20 °C	12 h	67 (10)
6 ^b^	MeCN	20 °C	3 h	75 (traces)
7 ^b^	MeCN	20 °C	6 h	92 (traces)
8 ^b^	MeCN	20 °C	12 h	96 (traces)
9 ^b^	MeCN	82 °C	1 h	34 (21)
10 ^c^	MeCN	20 °C	12 h	(40)

^a^ The yield of **9** is indicated in parentheses; ^b^ Conditions: (1) 1.3 equiv. **7a**, 1.2 equiv. **8a**; (2) NaHCO_3_; ^c^ isocyanide **7a** was not added.

## Data Availability

Dataset presented in this study is available in this article.

## Supplementary Materials

The following are available online.

## Sample Availability

Samples of the compounds are available on request from the authors.
