# Supplementary material for: Three-Component Reactions of 3-Arylidene-3H-Indolium Salts, Isocyanides and Amines
Source: Molecules. 2021 Apr 21;26(9):2402. doi: 10.3390/molecules26092402 (PMC8122609; doi:10.3390/molecules26092402)

Supplementary Material

Three-component reactions of 3-arylidene-3*H*-indolium salts, isocyanides and amines

Hung M. Nguyen [1,2], Nikita E. Golantsov [1,*] Alexandra S. Golubenkova [1], Victor B. Rybakov[3] and Leonid G. Voskressensky [1,*]

[1] Peoples' Friendship University of Russia (RUDN University), 6 Miklukho-Maklaya St., Moscow 117198, Russian Federation; golantsov_ne@pfur.ru (N.E.G.); aleksandra.golubenkova@mail.ru (A.S.G.); lvoskressensky@sci.pfu.edu.ru (L.G.V.)
[2] Hung Vuong University, Faculty of Natural Sciences, Nguyen Tat Thanh Street, Viet Tri 35120, Phu Tho, Vietnam; hunghoadhhv2008@gmail.com (H.M.N.)
[3] Lomonosov Moscow State University, Faculty of Chemistry, 1-3 Leninskiye Gory, Moscow 119991, Russian Federation; rybakov20021@yandex.ru (V.B.R.)

* Correspondence: golantsov_ne@pfur.ru (N.E.G.); lvoskressensky@sci.pfu.edu.ru (L.G.V.); Tel.: +7-916-324-0671 (N.E.G.); +7-916-913-6242 (L.G.V.)

Contents

1. ^{1}H and ^{13}C NMR spectra of imidamides **1g, h** 2-3
2. ^{1}H and ^{13}C NMR spectra of imidamides **2a-j** 3-13
3. ^{1}H and ^{13}C NMR spectra of amine **9** 14
4. ^{1}H and ^{13}C NMR spectra of imidazolones **3a-s** 15-33
5. ^{1}H and ^{13}C NMR spectra of imidamides **10a-f** 34-39

1. ^{1}H and ^{13}C NMR spectra of imidamides **1g, h**

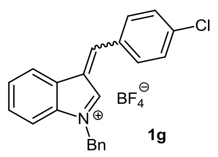

1g

^{1}H NMR (600 MHz, TFA+$CDCl_3$)

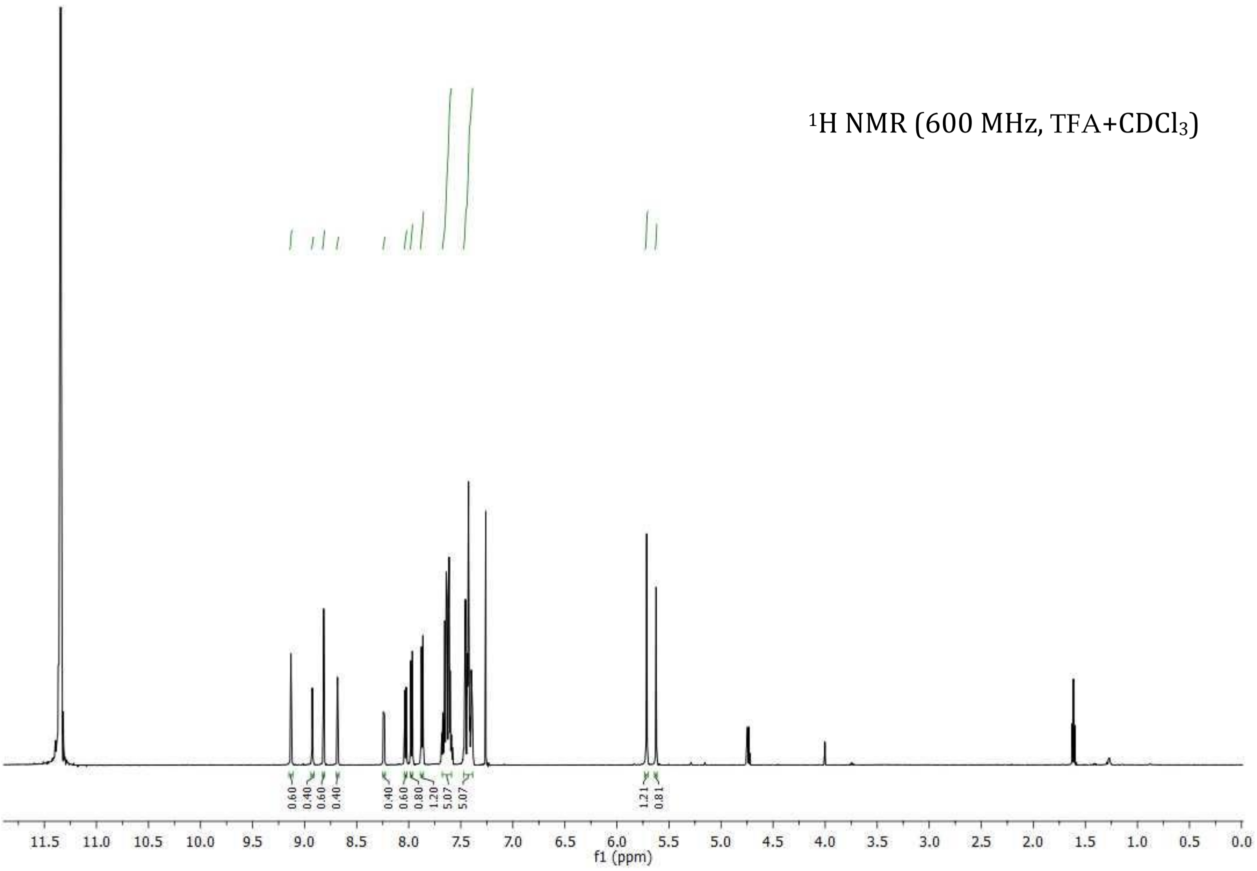

^{13}C NMR (150 MHz, TFA+$CDCl_3$)

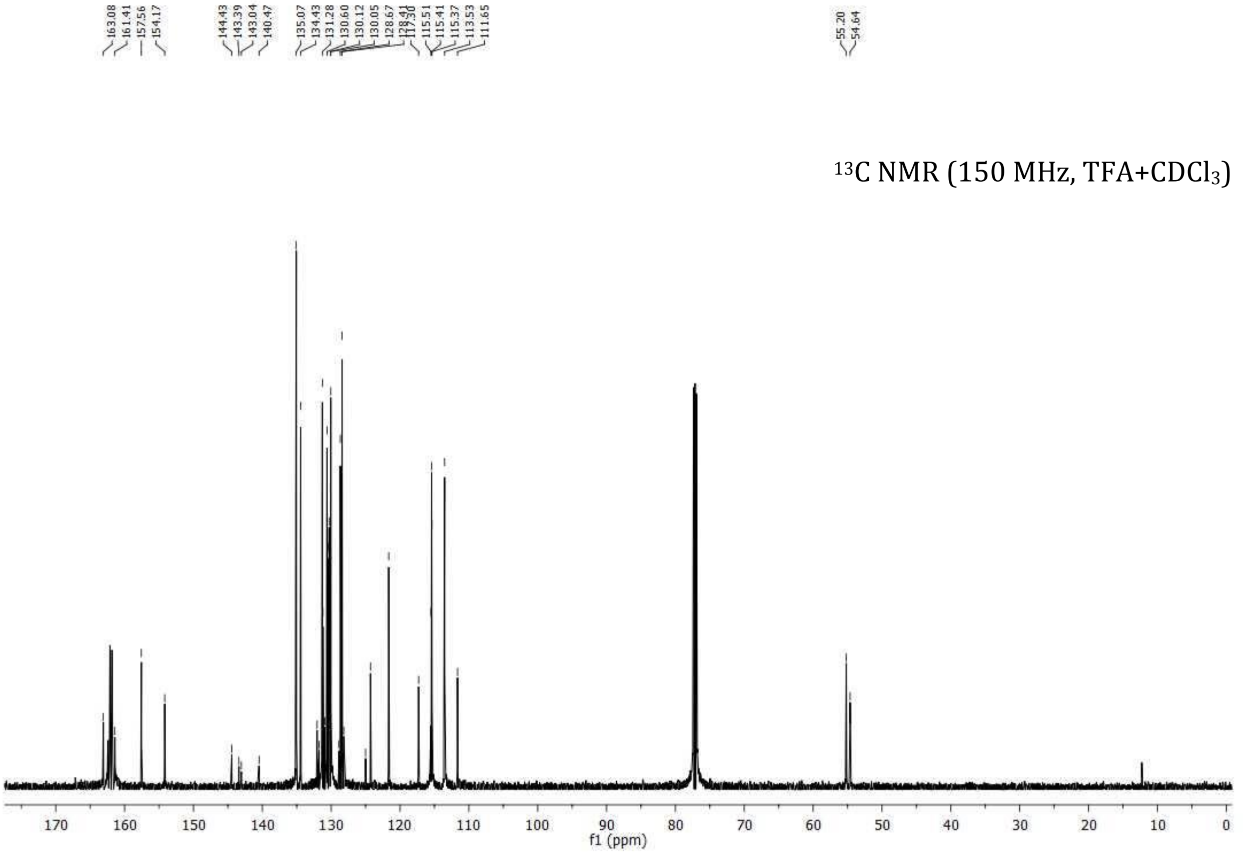

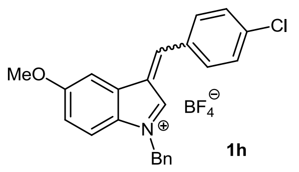

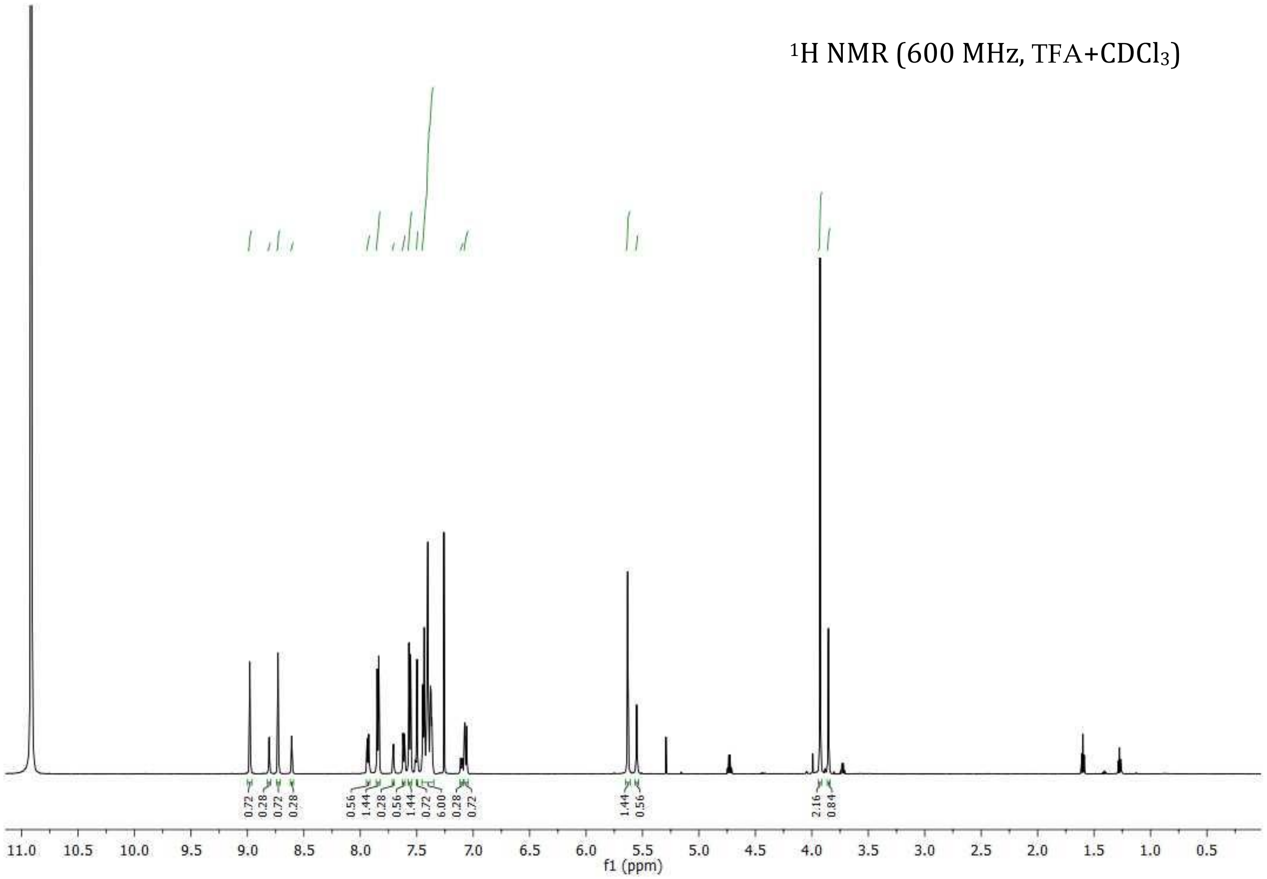

^{1}H NMR (600 MHz, TFA+$CDCl_3$)

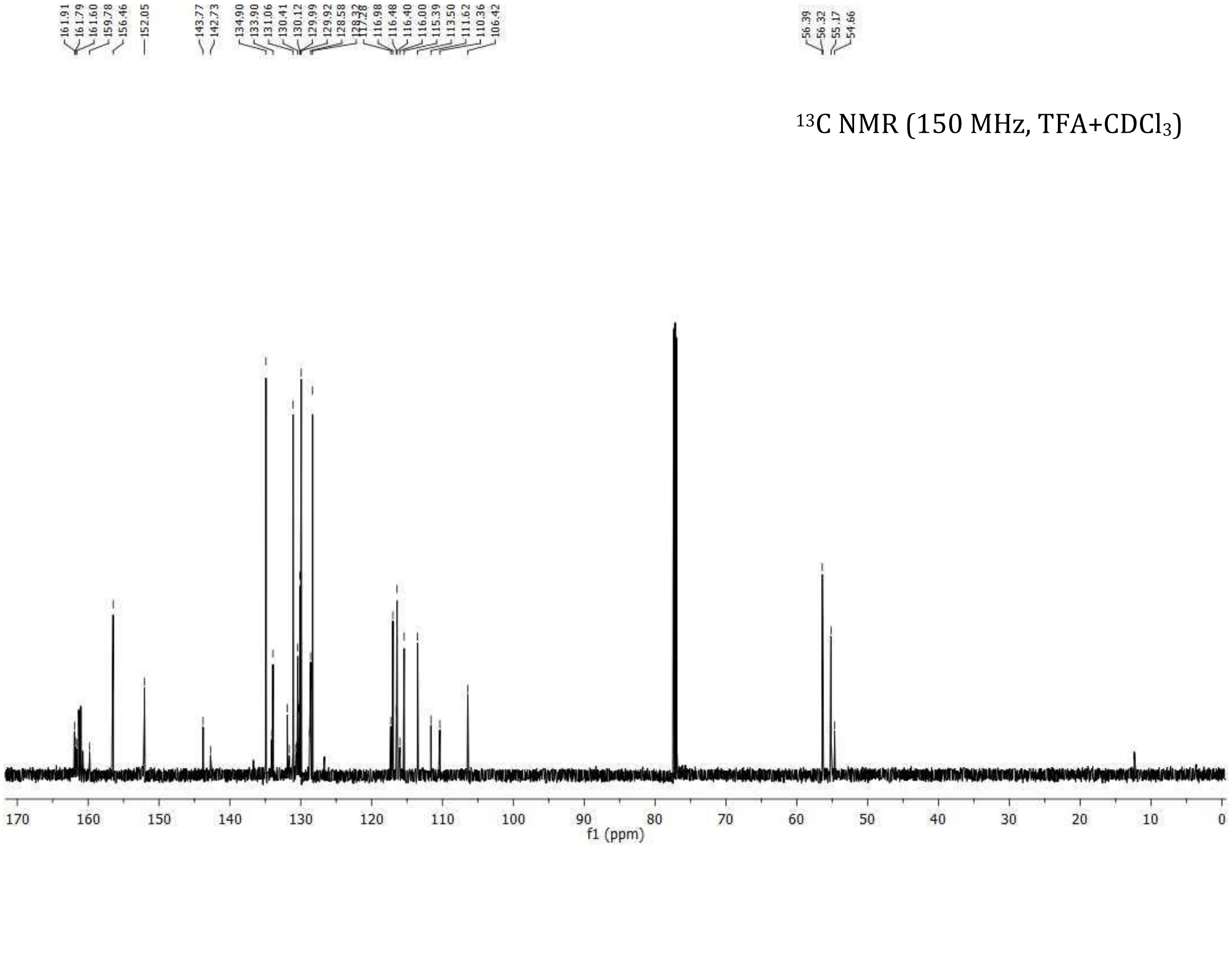

^{13}C NMR (150 MHz, TFA+$CDCl_3$)

2. ^{1}H and ^{13}C NMR spectra of imidamides **2a-j**

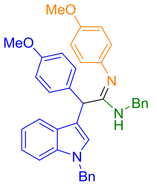

2a

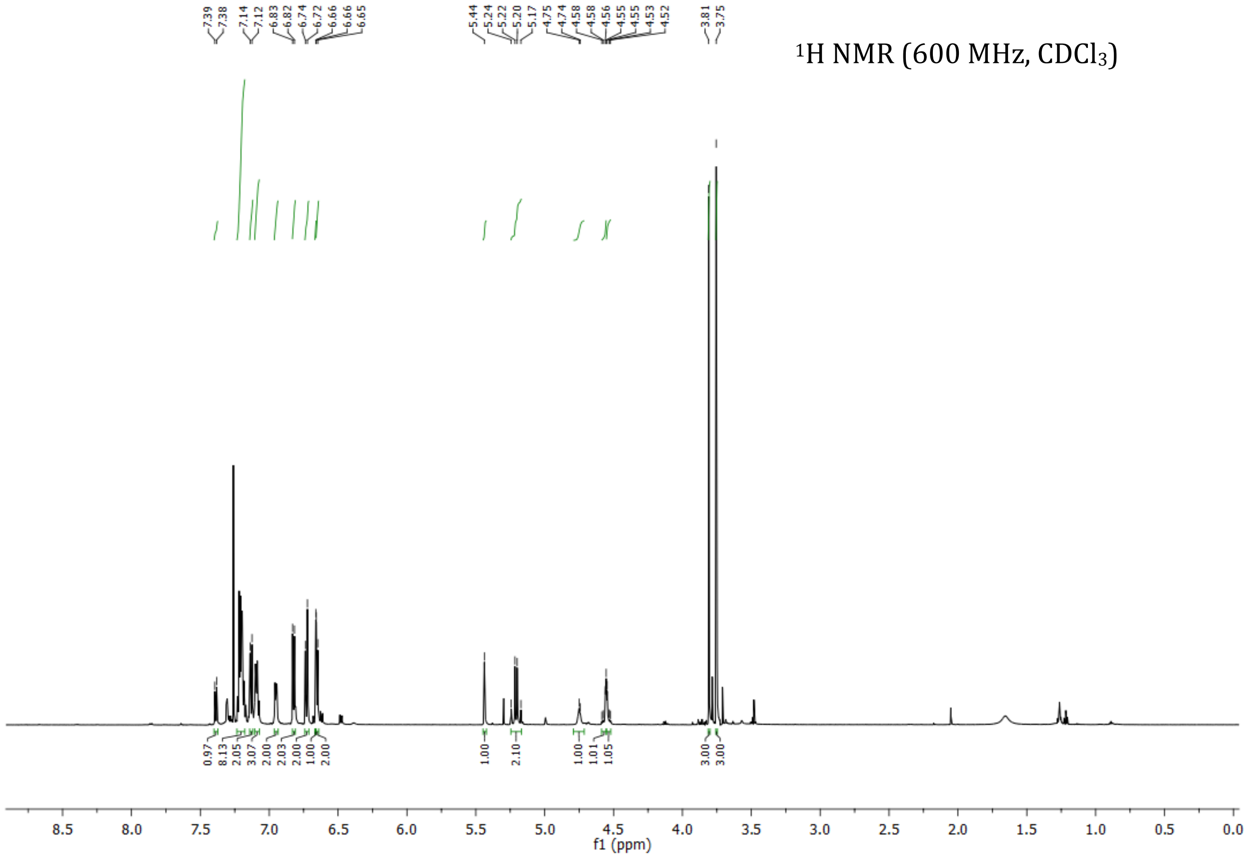

^{1}H NMR (600 MHz, $CDCl_3$)

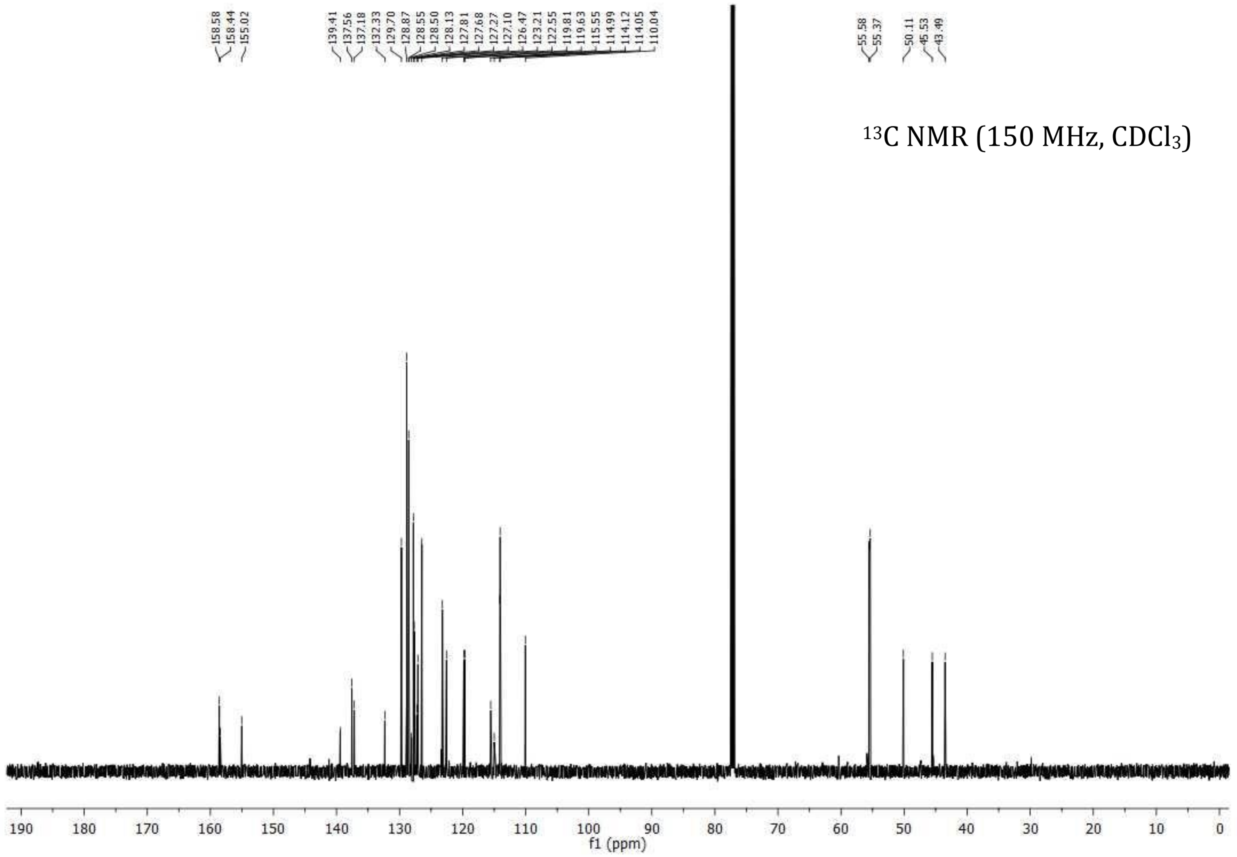

^{13}C NMR (150 MHz, $CDCl_3$)

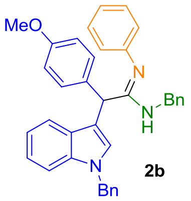
MeO
N
N
H
Bn
N
Bn

2b

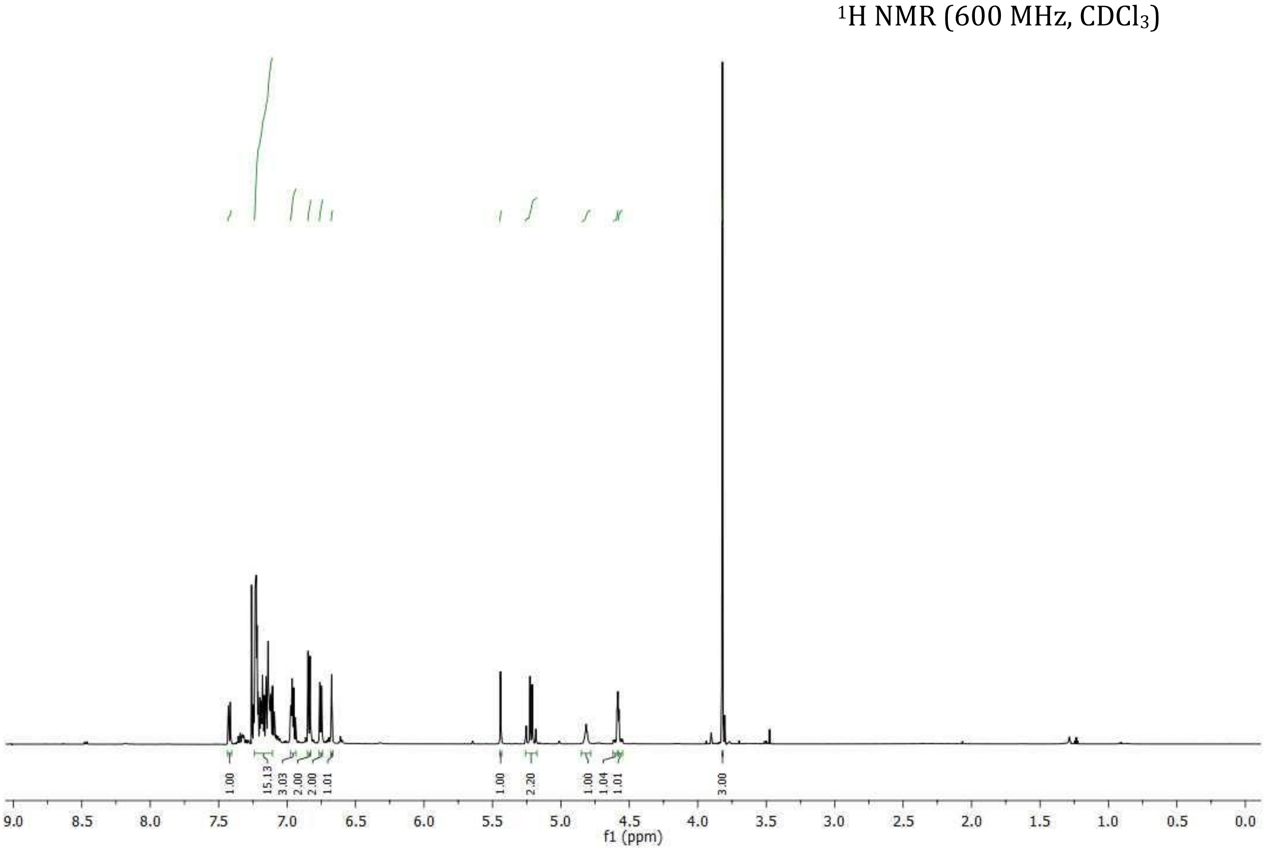
1H NMR (600 MHz, CDCl3)
1.00
15.13
3.03
2.00
2.00
1.01
1.00
2.20
1.00
1.04
1.01
3.00
9.0
8.5
8.0
7.5
7.0
6.5
6.0
5.5
5.0
4.5
4.0
3.5
3.0
2.5
2.0
1.5
1.0
0.5
0.0
f1 (ppm)

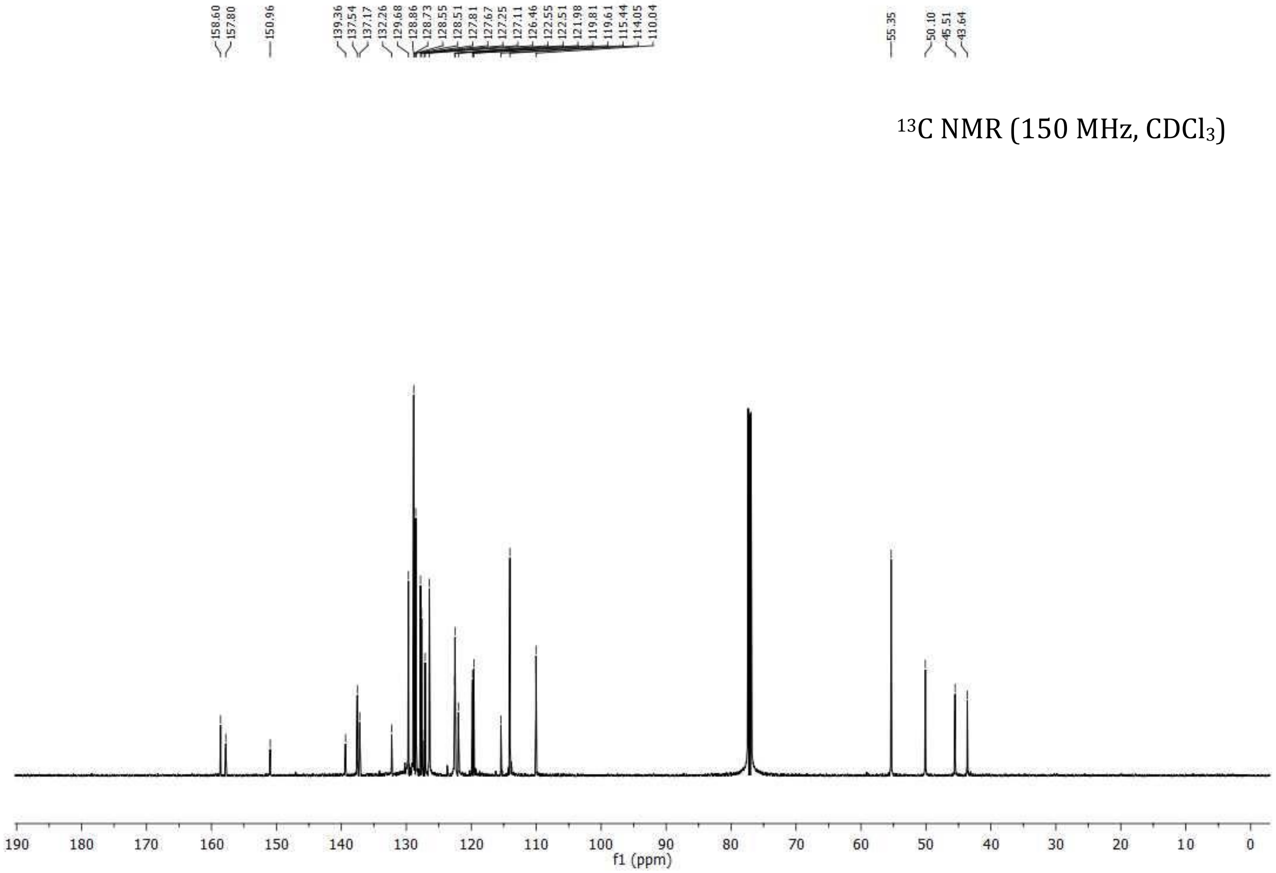
158.60
157.80
150.96
139.36
137.54
137.17
132.26
129.68
128.86
128.73
128.55
128.51
127.81
127.67
127.25
127.11
126.46
122.55
122.51
121.98
119.81
119.61
115.44
114.05
110.04
55.35
50.10
45.51
43.64
13C NMR (150 MHz, CDCl3)
190
180
170
160
150
140
130
120
110
100
90
80
70
60
50
40
30
20
10
0
f1 (ppm)

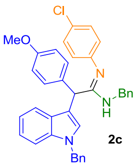

2c

^{1}H NMR (600 MHz, $CDCl_3$)

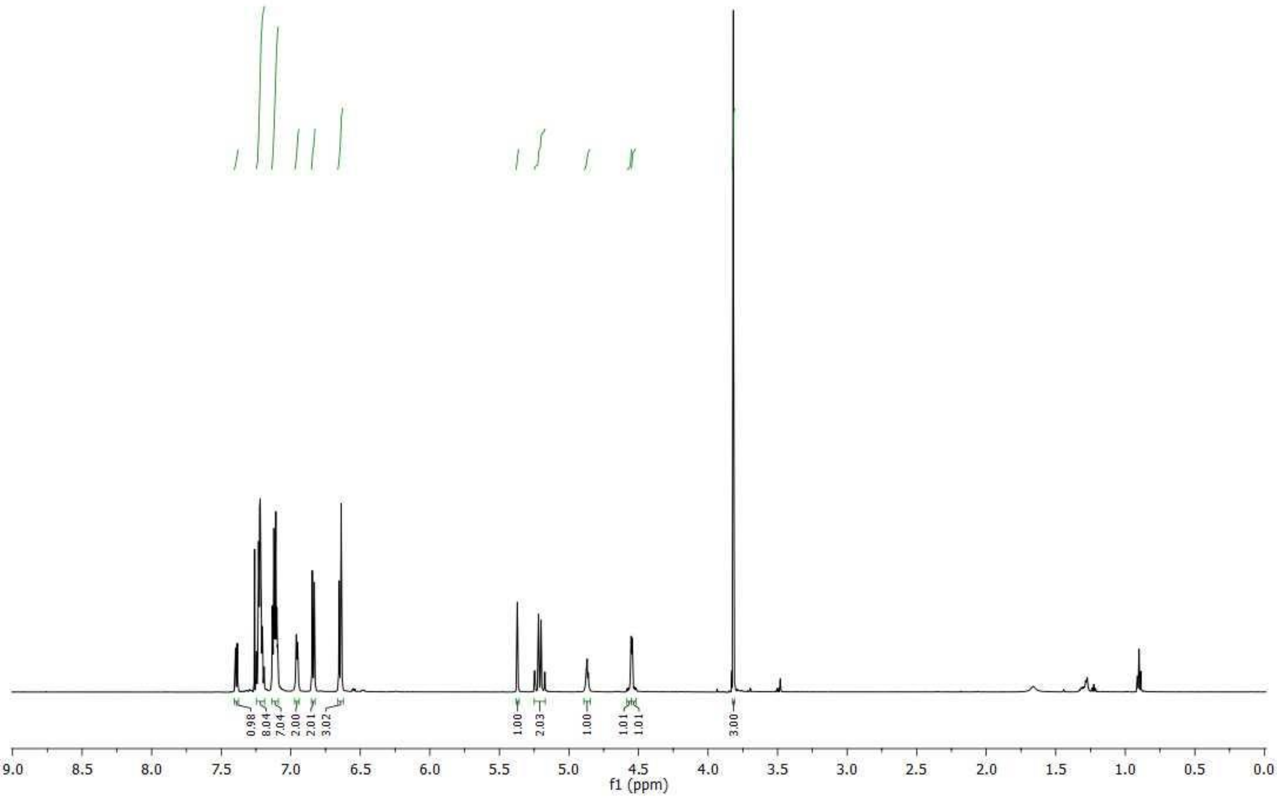

^{13}C NMR (150 MHz, $CDCl_3$)

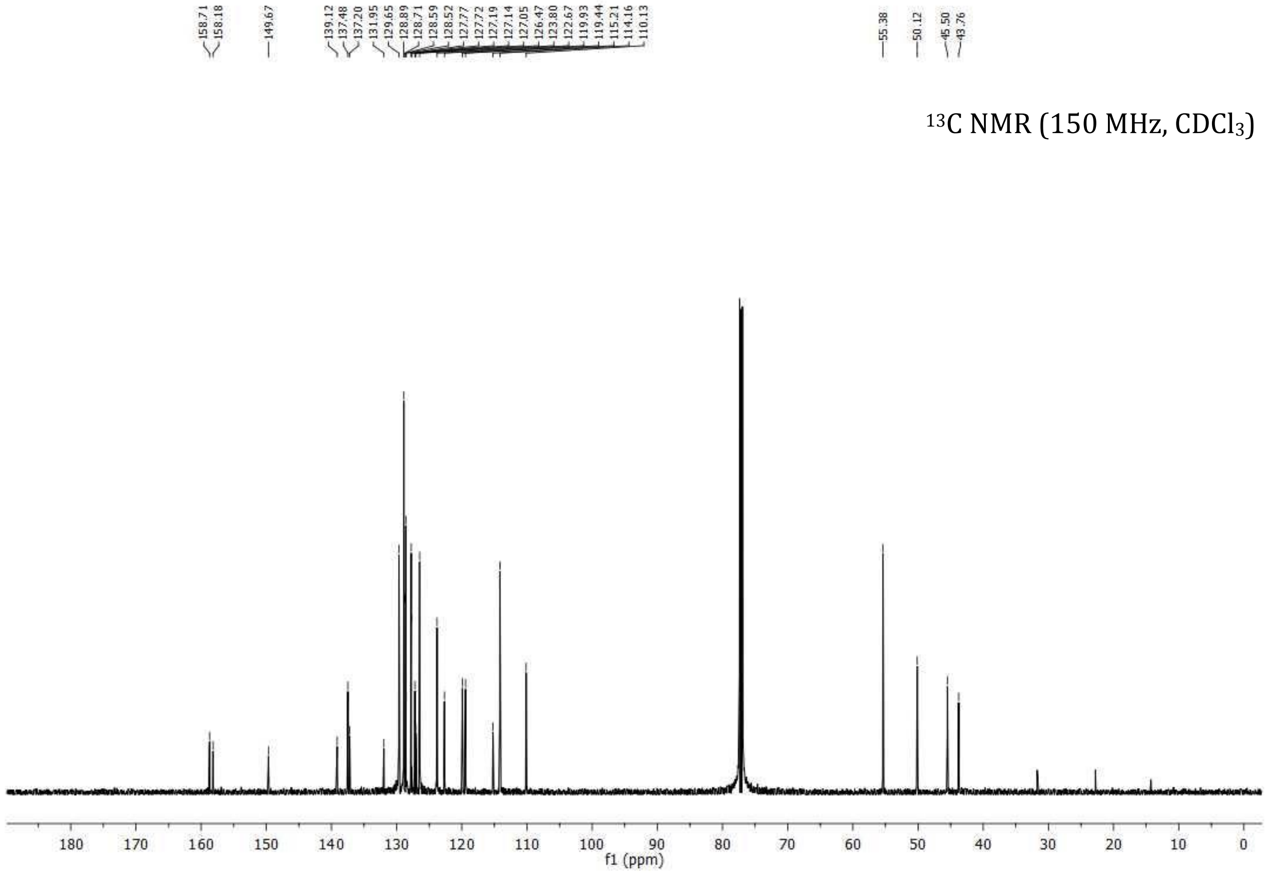

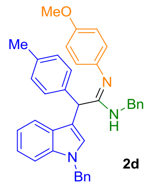
MeO
Me
N
N
H
Bn
N
Bn

2d

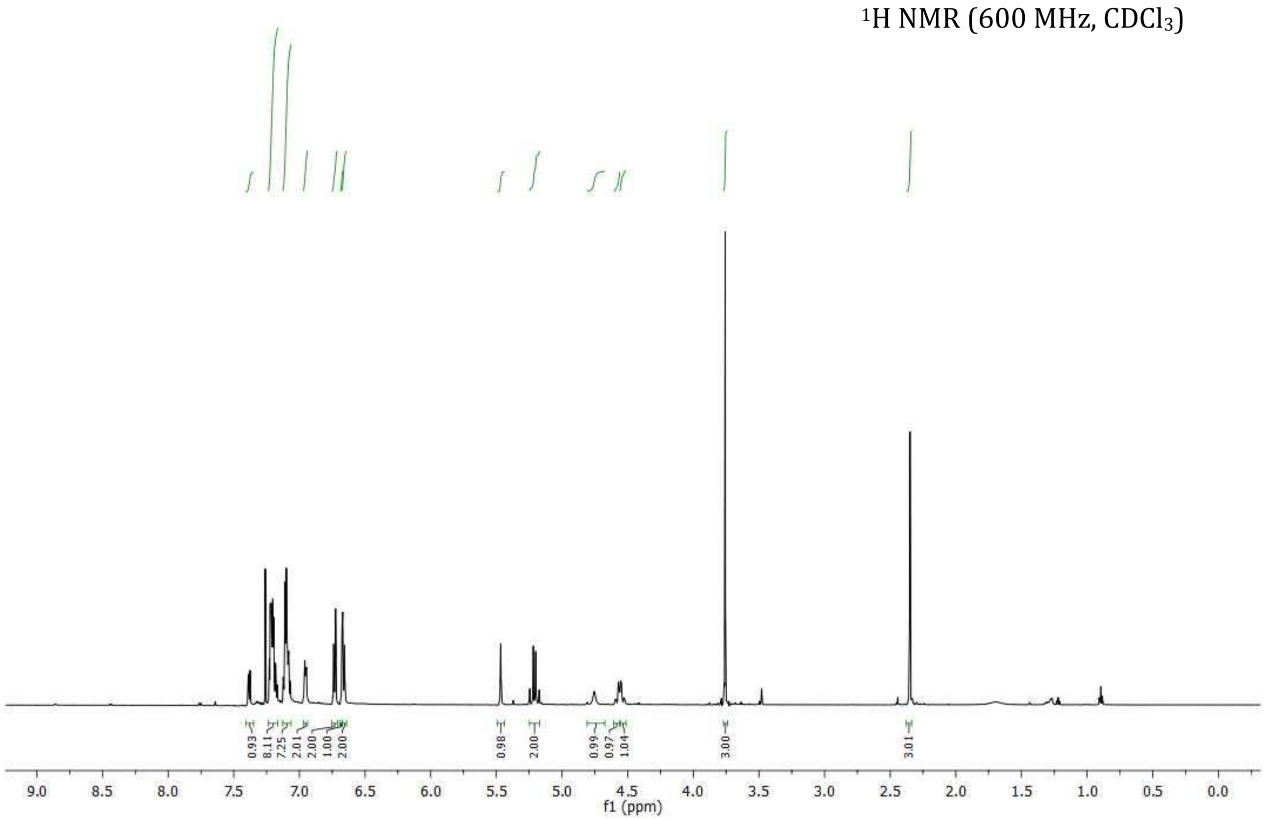
1H NMR (600 MHz, CDCl3)
f1 (ppm)

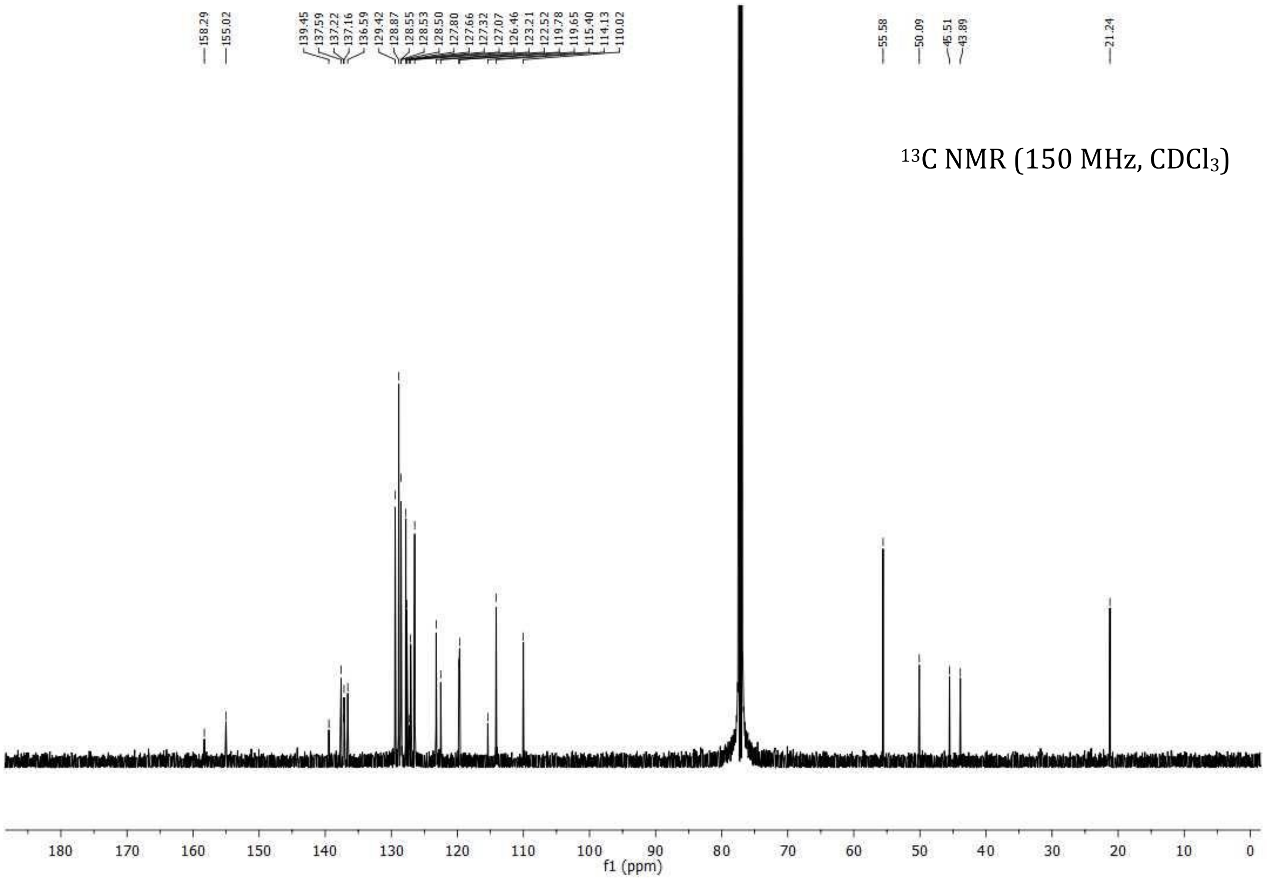
13C NMR (150 MHz, CDCl3)
f1 (ppm)

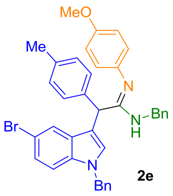

2e

^{1}H NMR (600 MHz, $CDCl_3$)

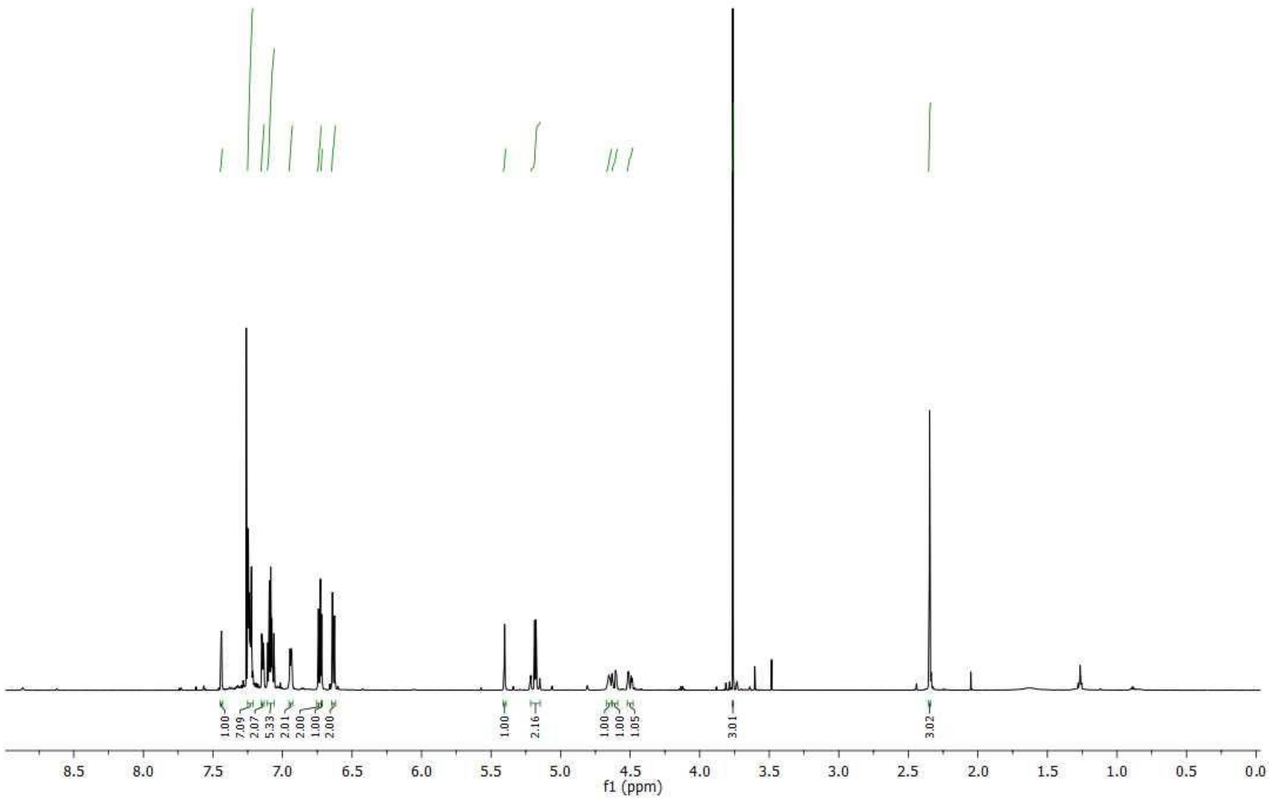

^{13}C NMR (150 MHz, $CDCl_3$)

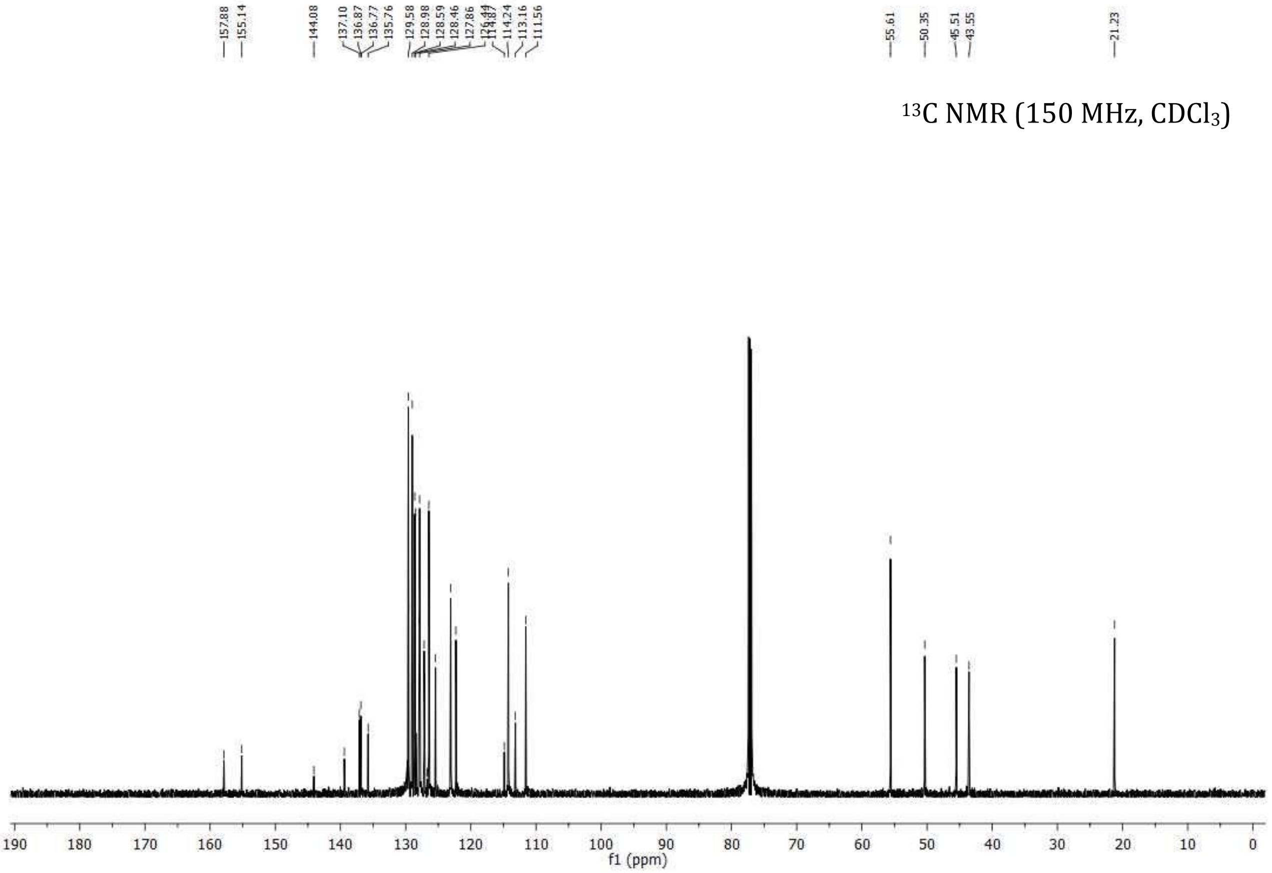

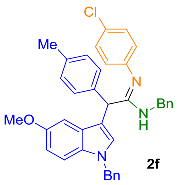

2f

^{1}H NMR (600 MHz, $CDCl_3$)

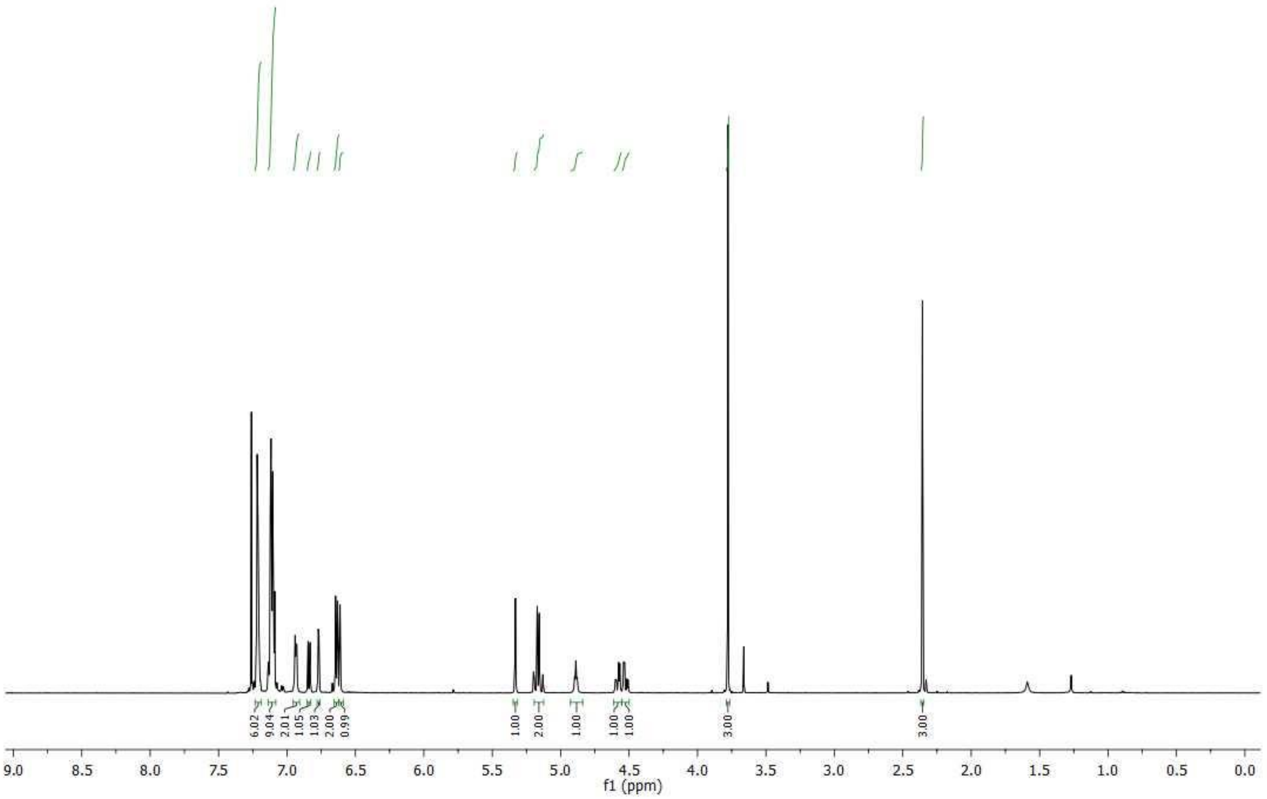

^{13}C NMR (150 MHz, $CDCl_3$)

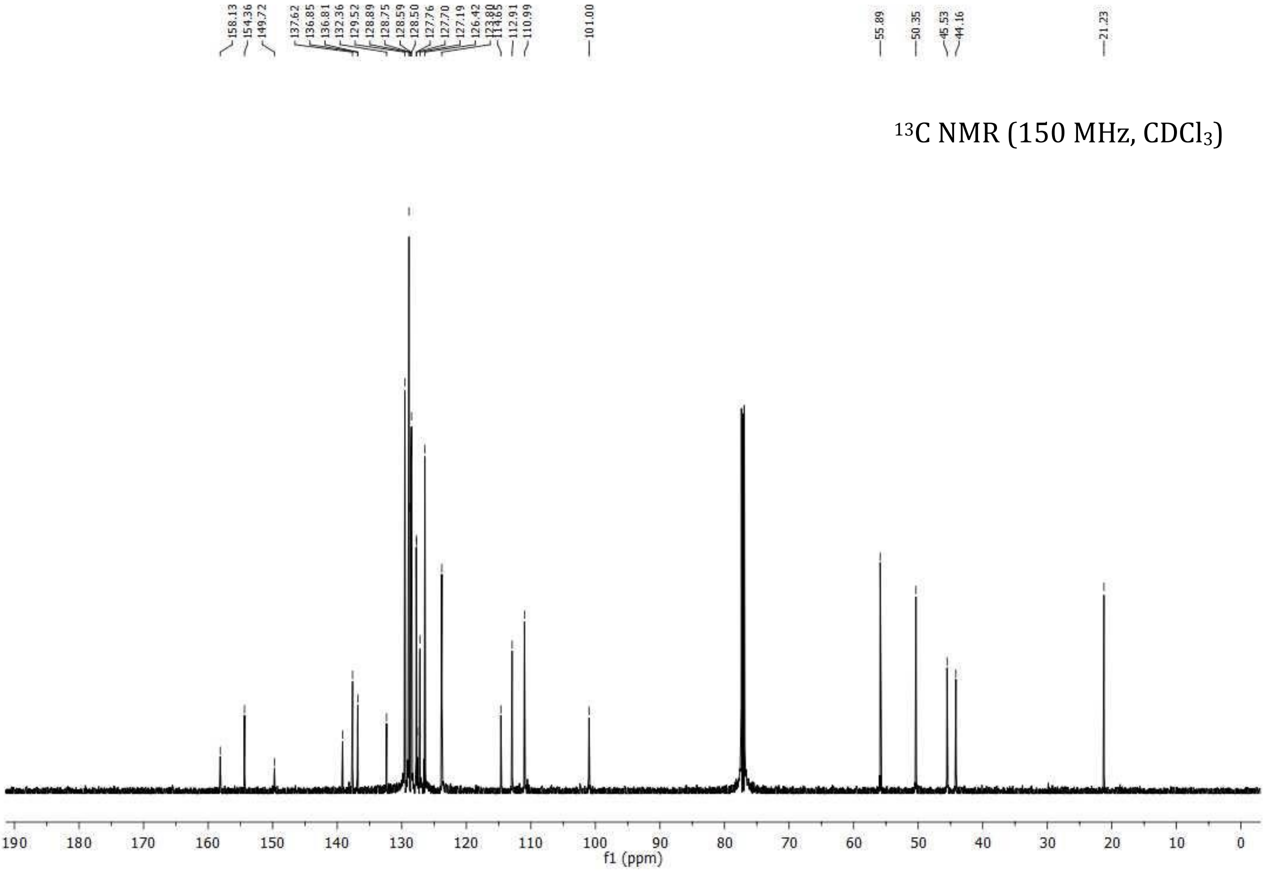

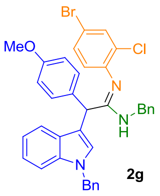

2g

^{1}H NMR (600 MHz, $CDCl_3$)

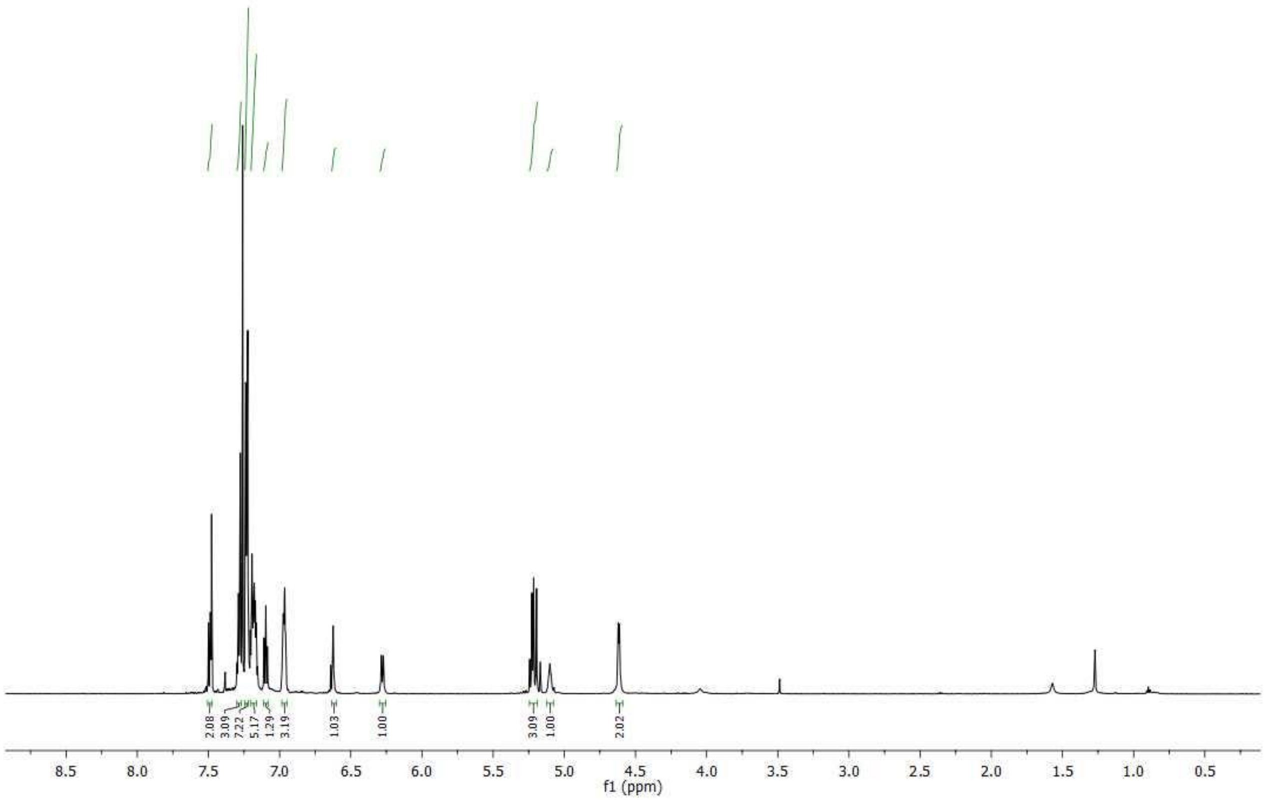

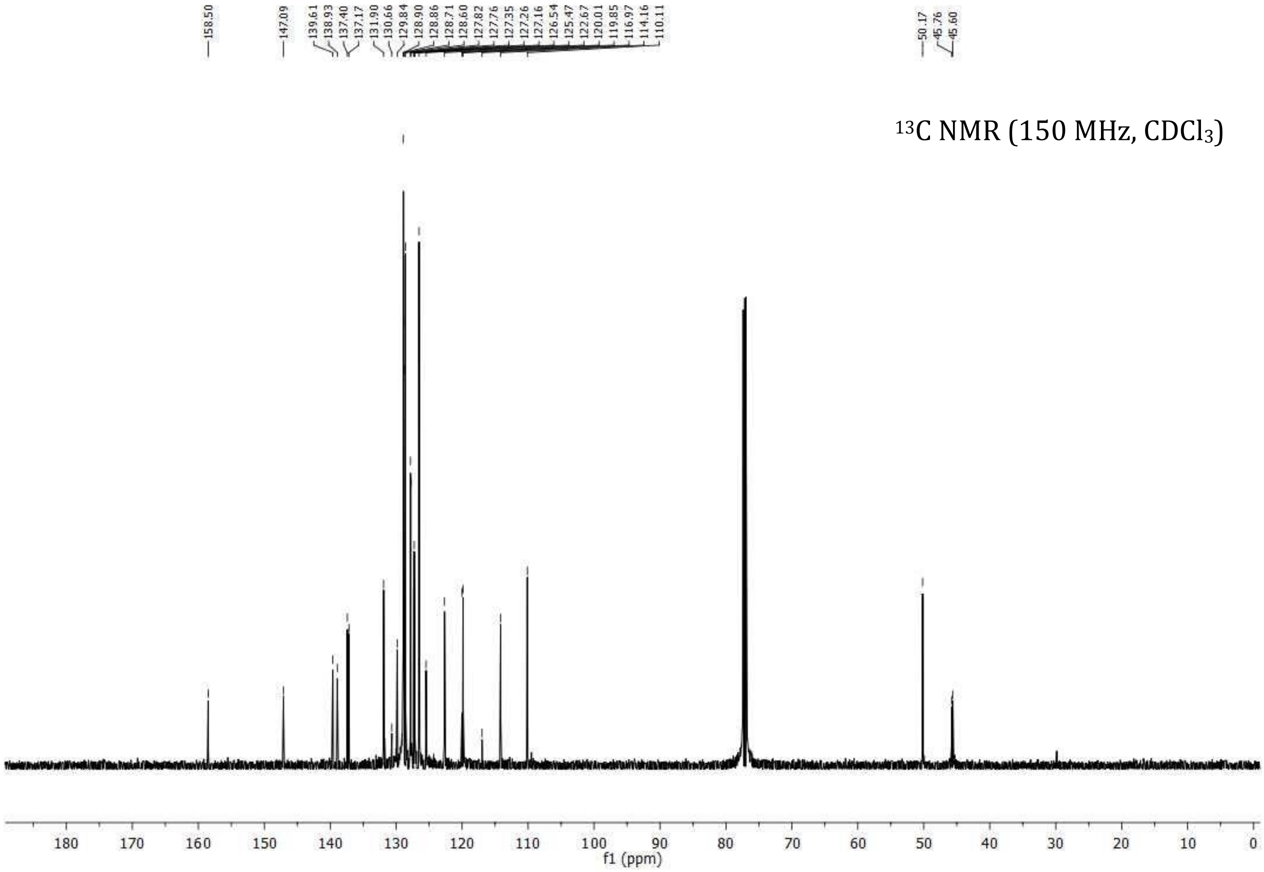

^{13}C NMR (150 MHz, $CDCl_3$)

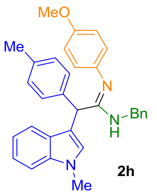

2h

^{1}H NMR (600 MHz, $CDCl_3$)

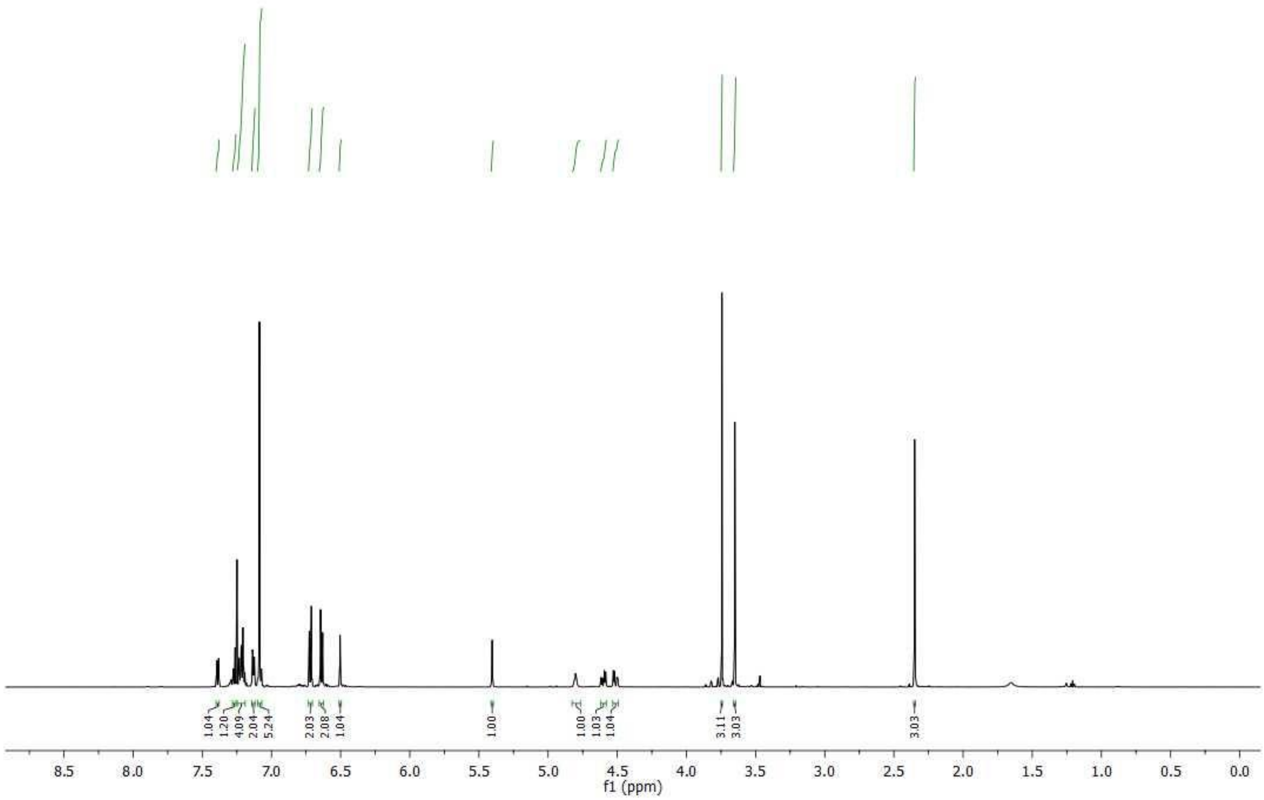

^{13}C NMR (150 MHz, $CDCl_3$)

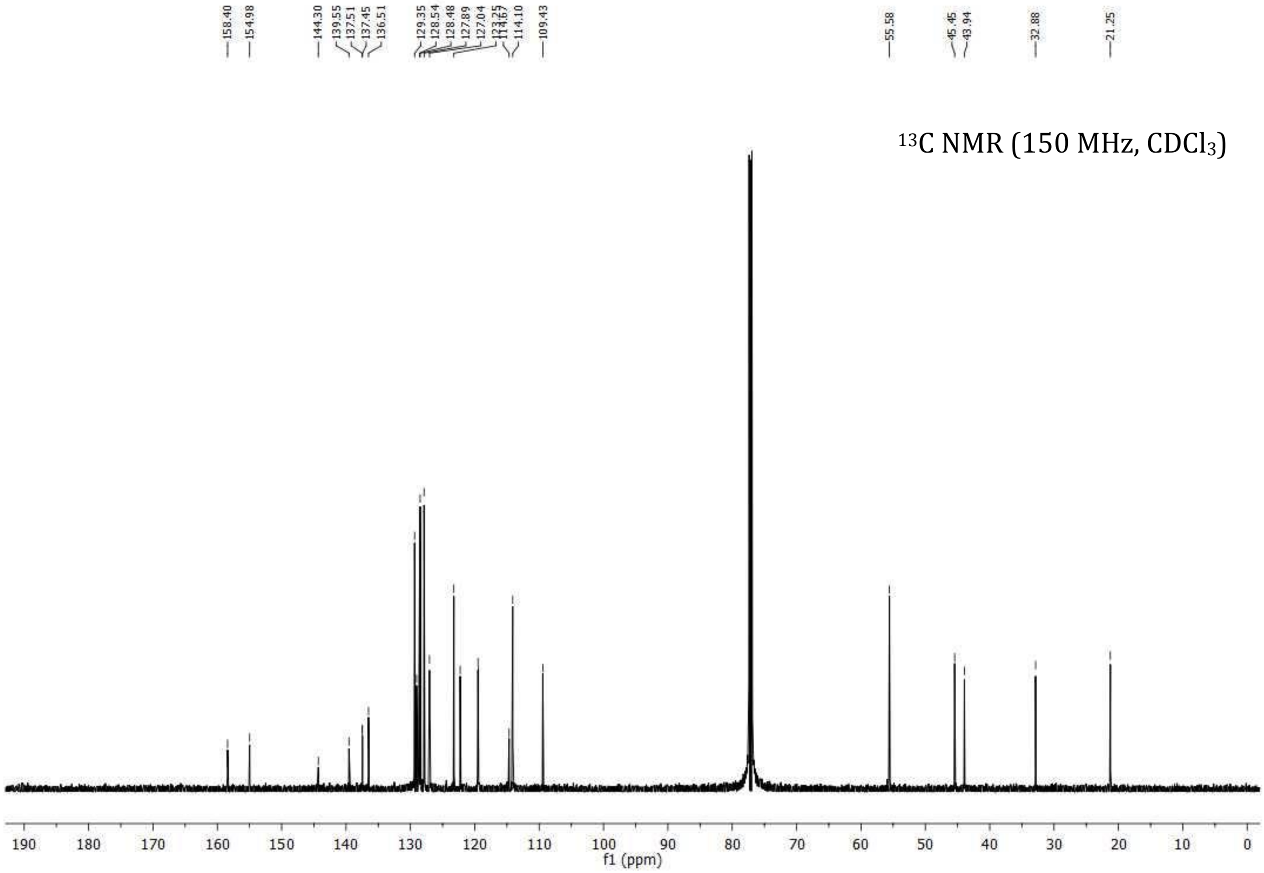

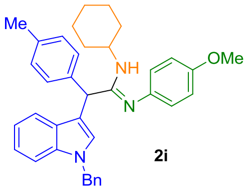

2i

^{1}H NMR (600 MHz, $CDCl_3$)

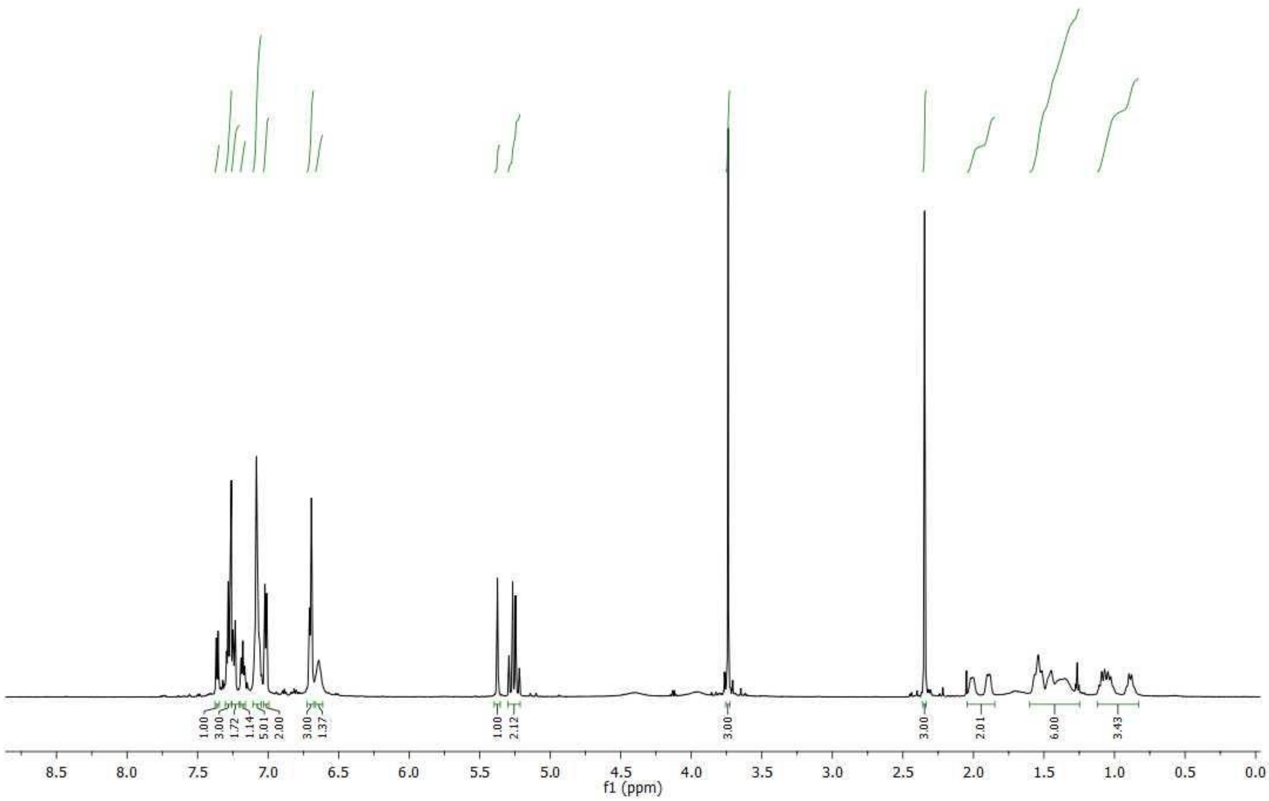

^{13}C NMR (150 MHz, $CDCl_3$)

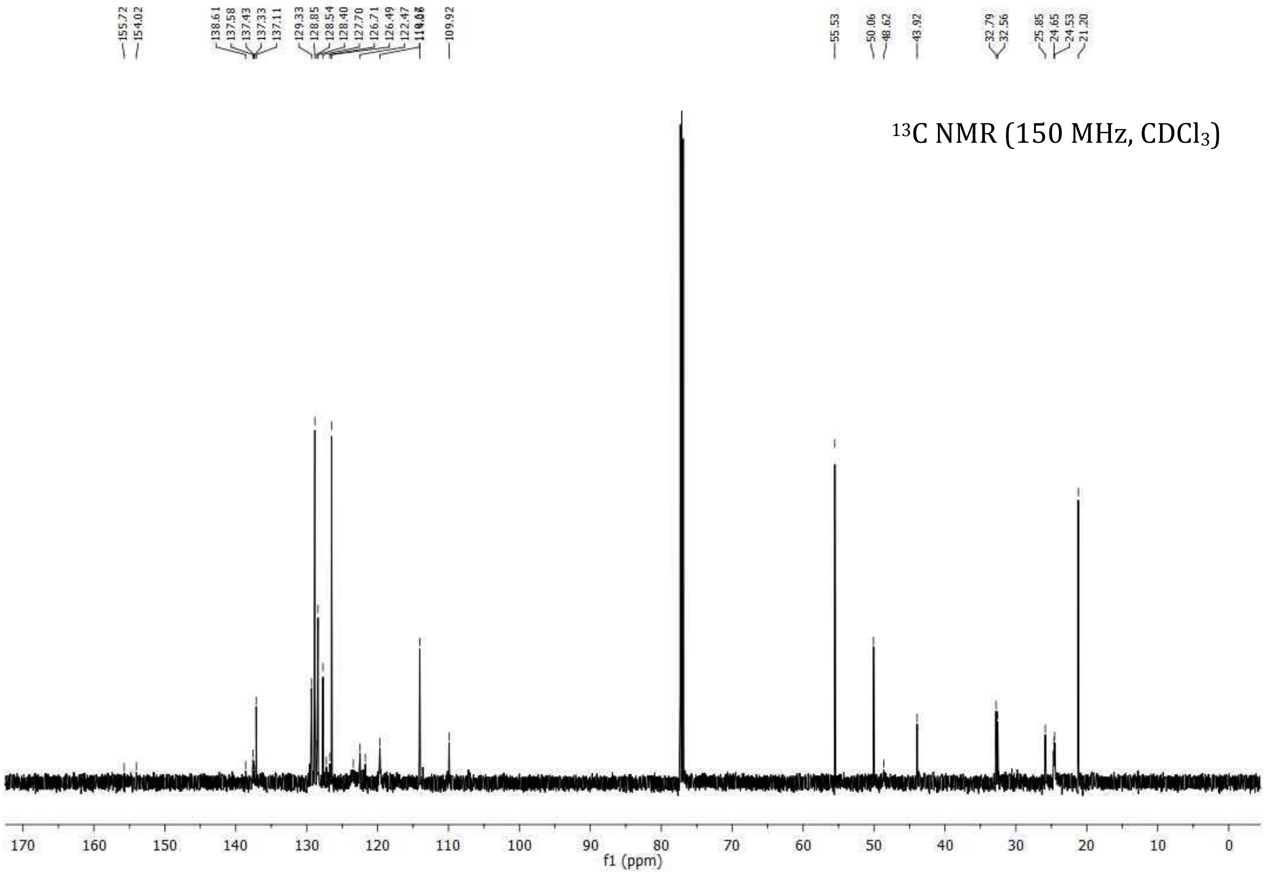

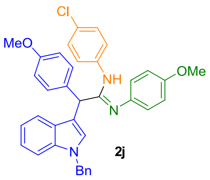

2j

^{1}H NMR (600 MHz, $CDCl_3$)

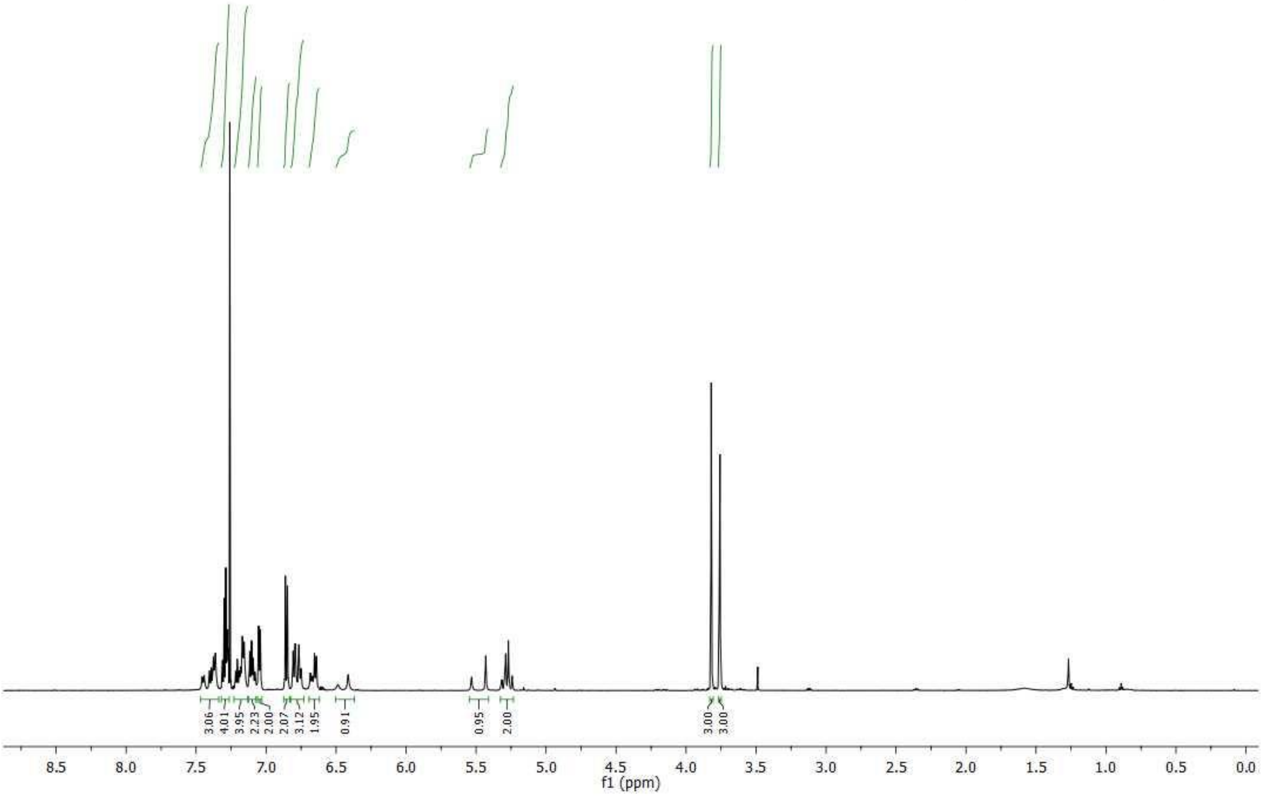

^{13}C NMR (150 MHz, $CDCl_3$)

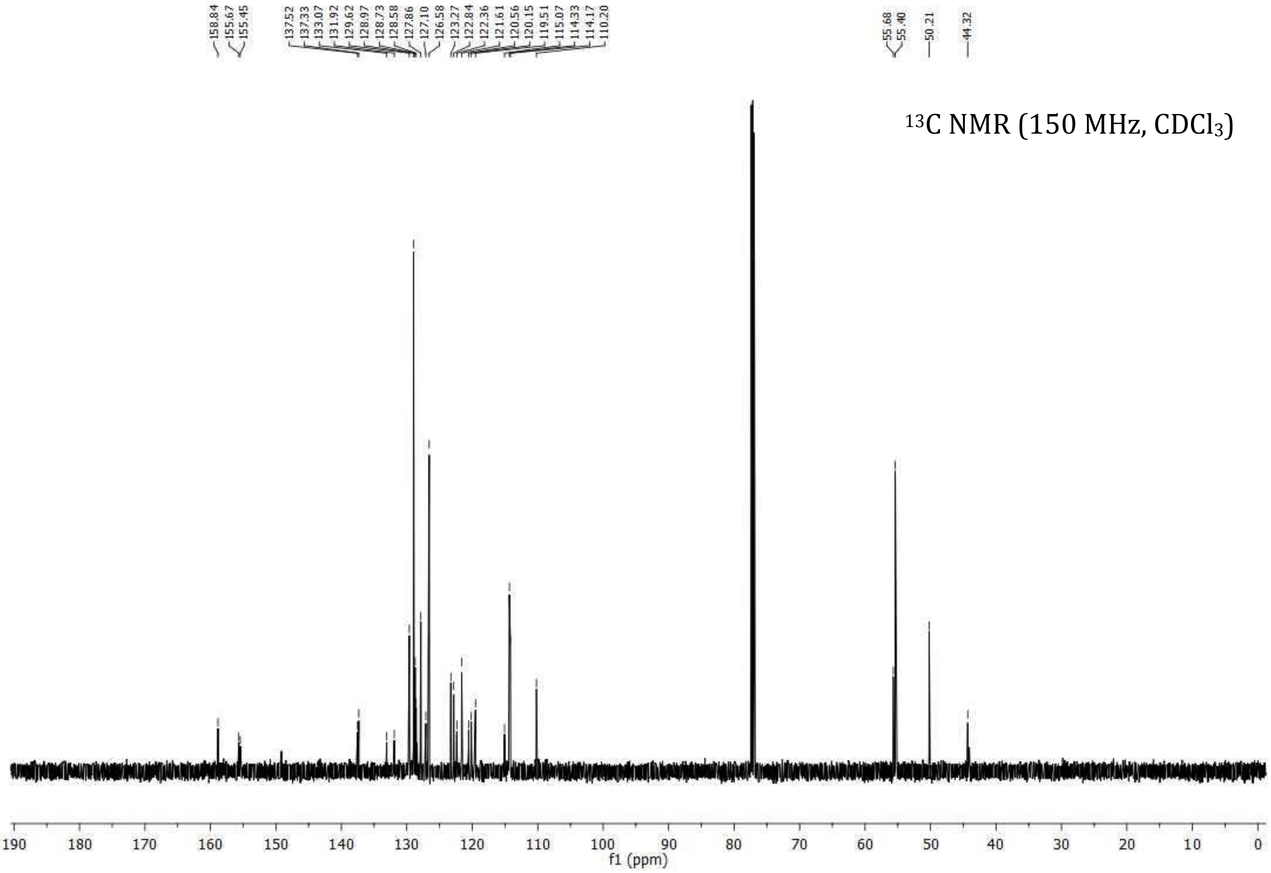

3. ^{1}H and ^{13}C NMR spectra of amine **9**

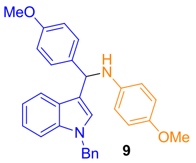

^{1}H NMR (600 MHz, $CDCl_3$)

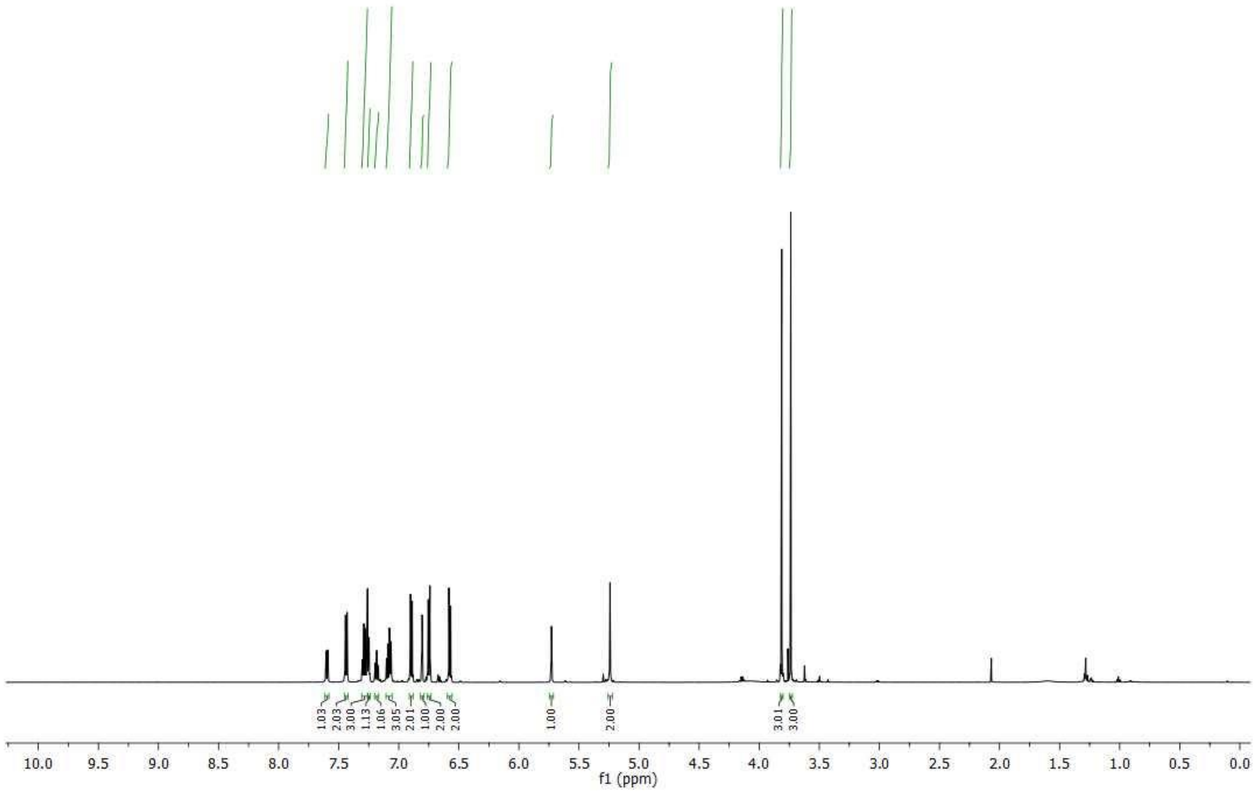

^{13}C NMR (150 MHz, $CDCl_3$)

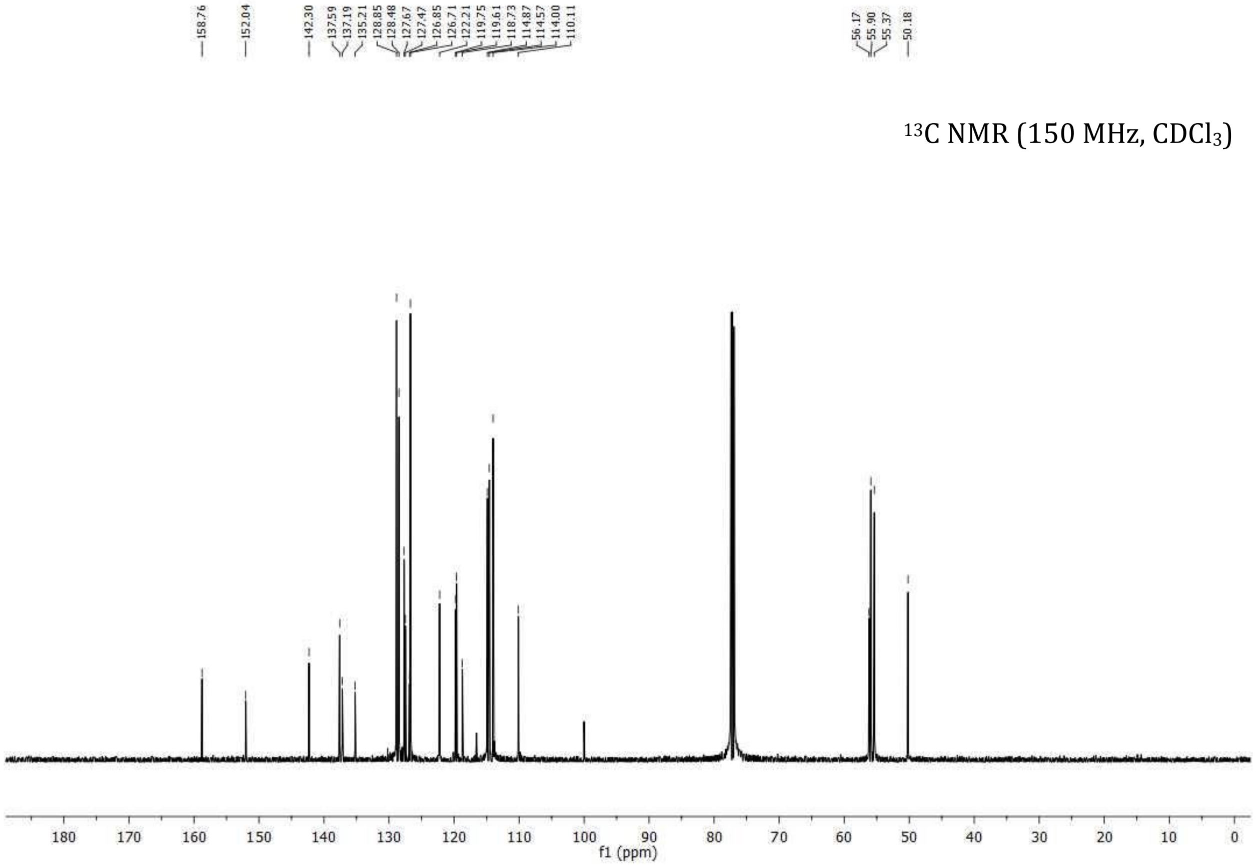

4. ^{1}H and ^{13}C NMR spectra of imidazolones **3a-s**

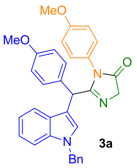

^{1}H NMR (600 MHz, $CDCl_3$)

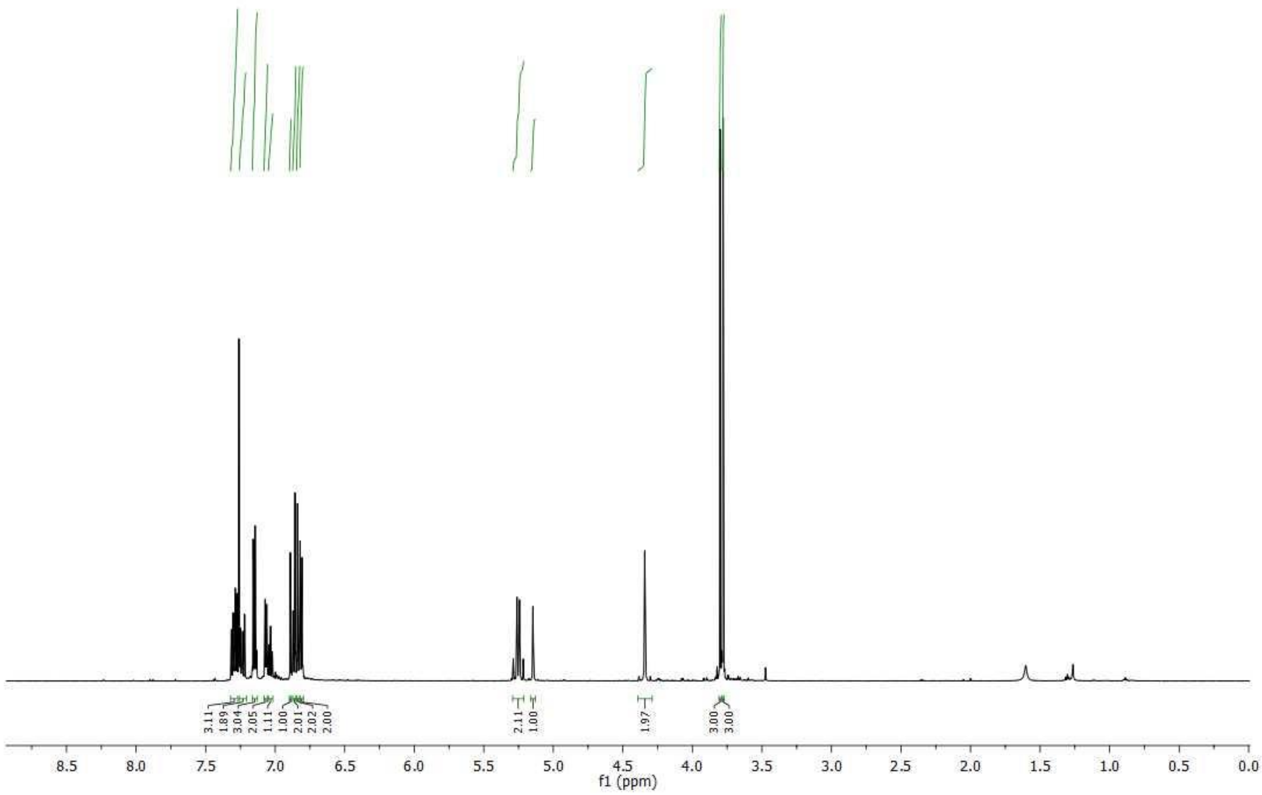

^{13}C NMR (150 MHz, $CDCl_3$)

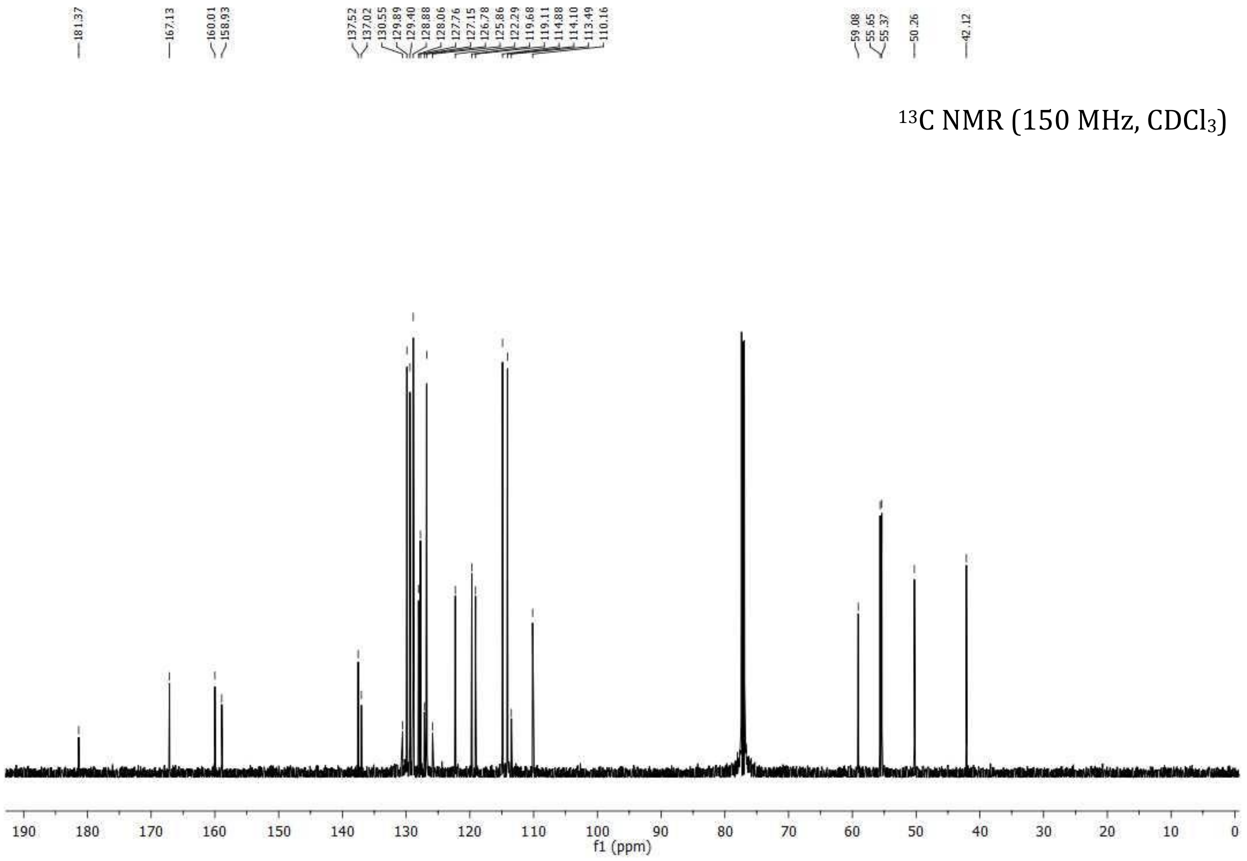

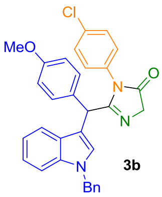

3b

^{1}H NMR (600 MHz, $CDCl_3$)

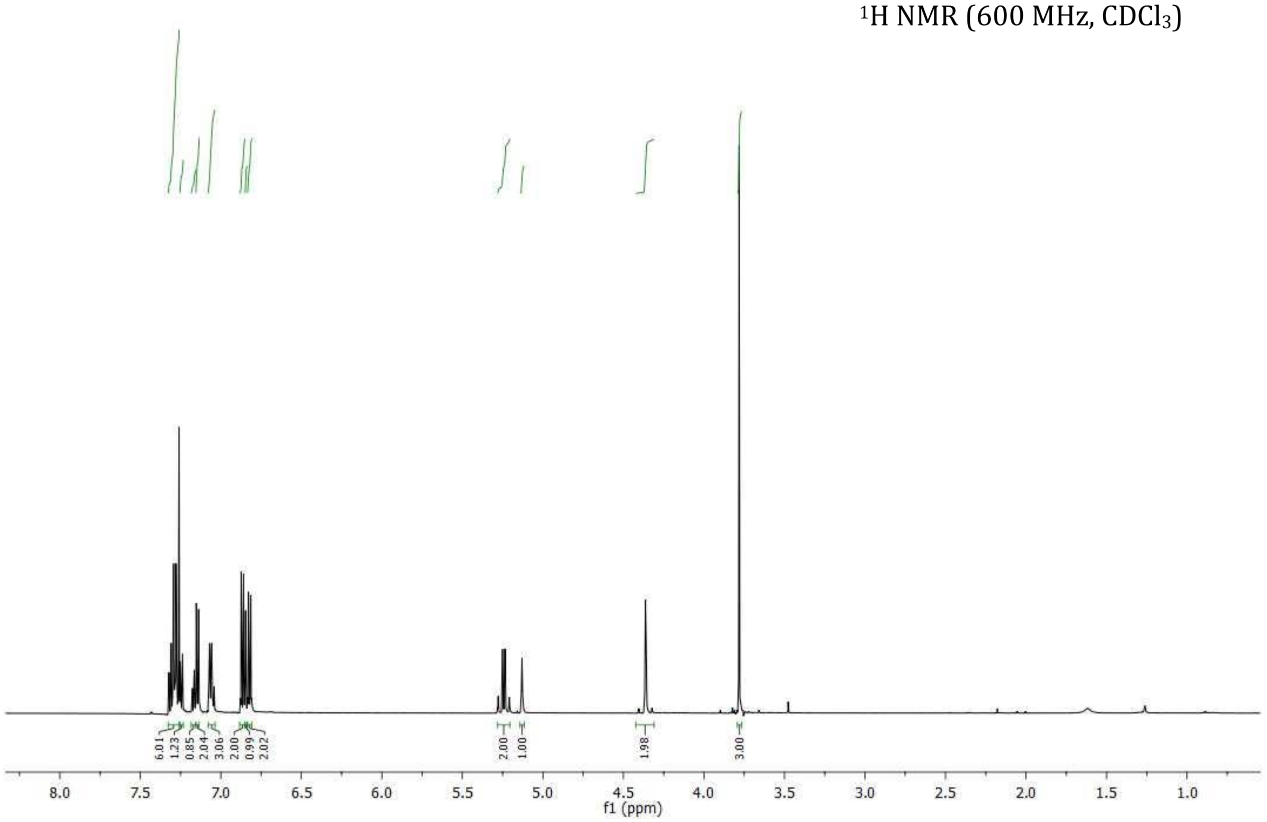

^{13}C NMR (150 MHz, $CDCl_3$)

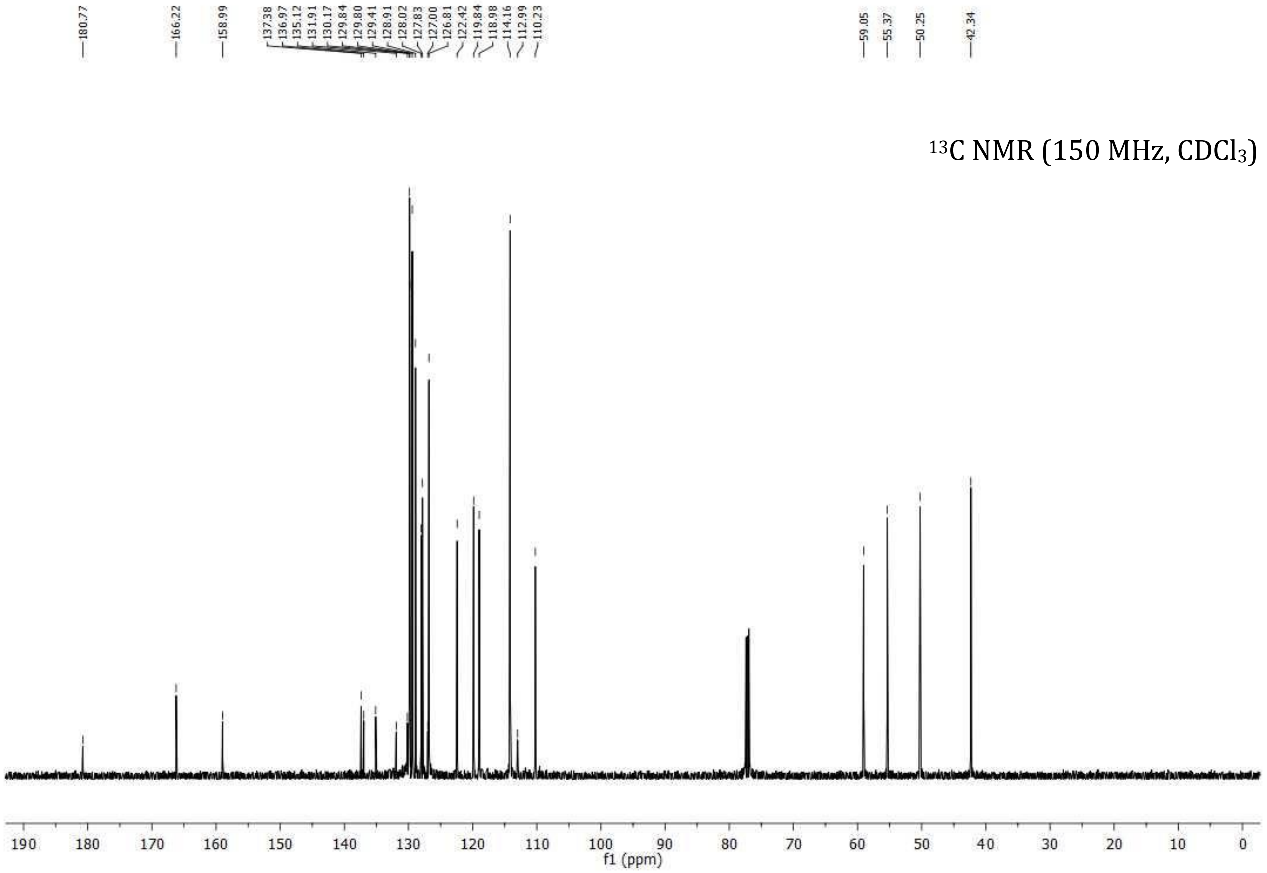

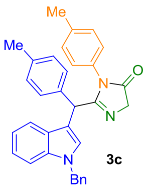

^{1}H NMR (600 MHz, $CDCl_3$)

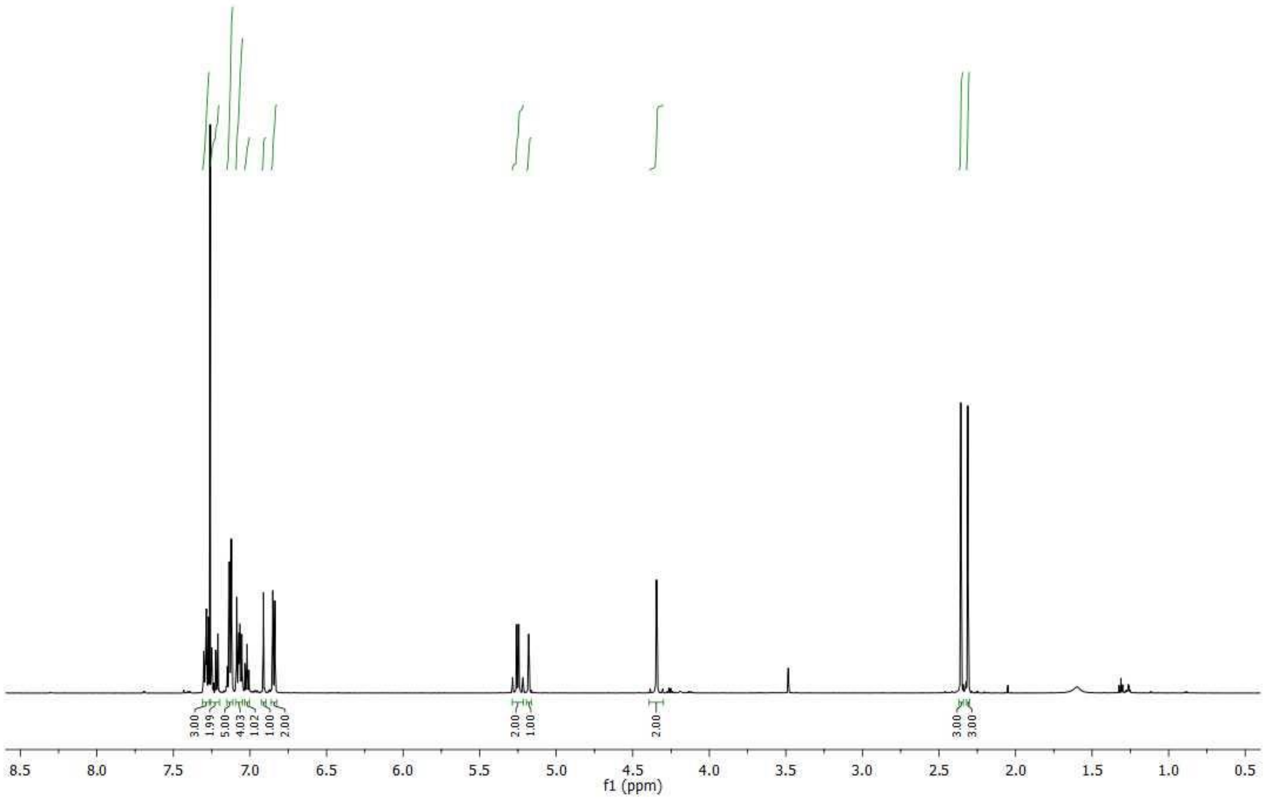

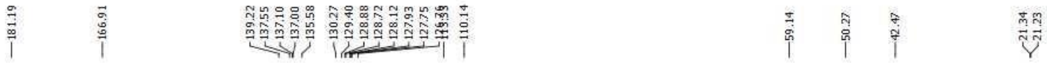

^{13}C NMR (150 MHz, $CDCl_3$)

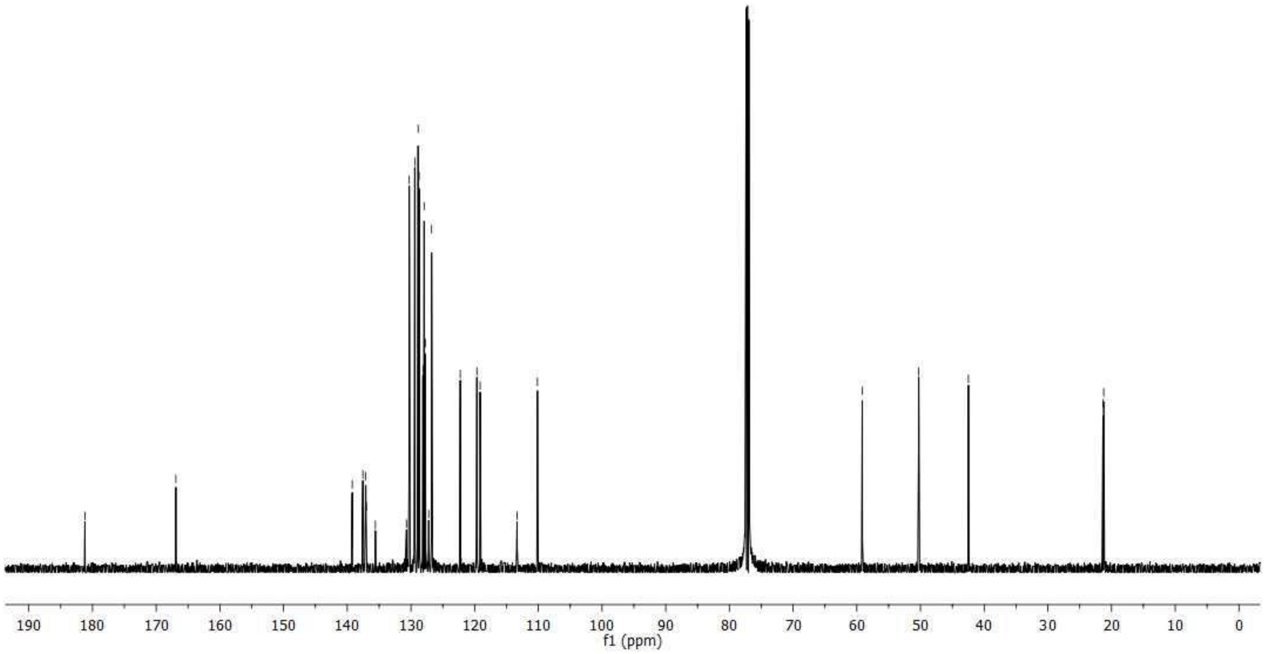

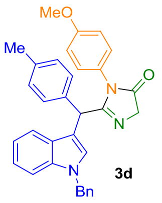

3d

^{1}H NMR (600 MHz, $CDCl_3$)

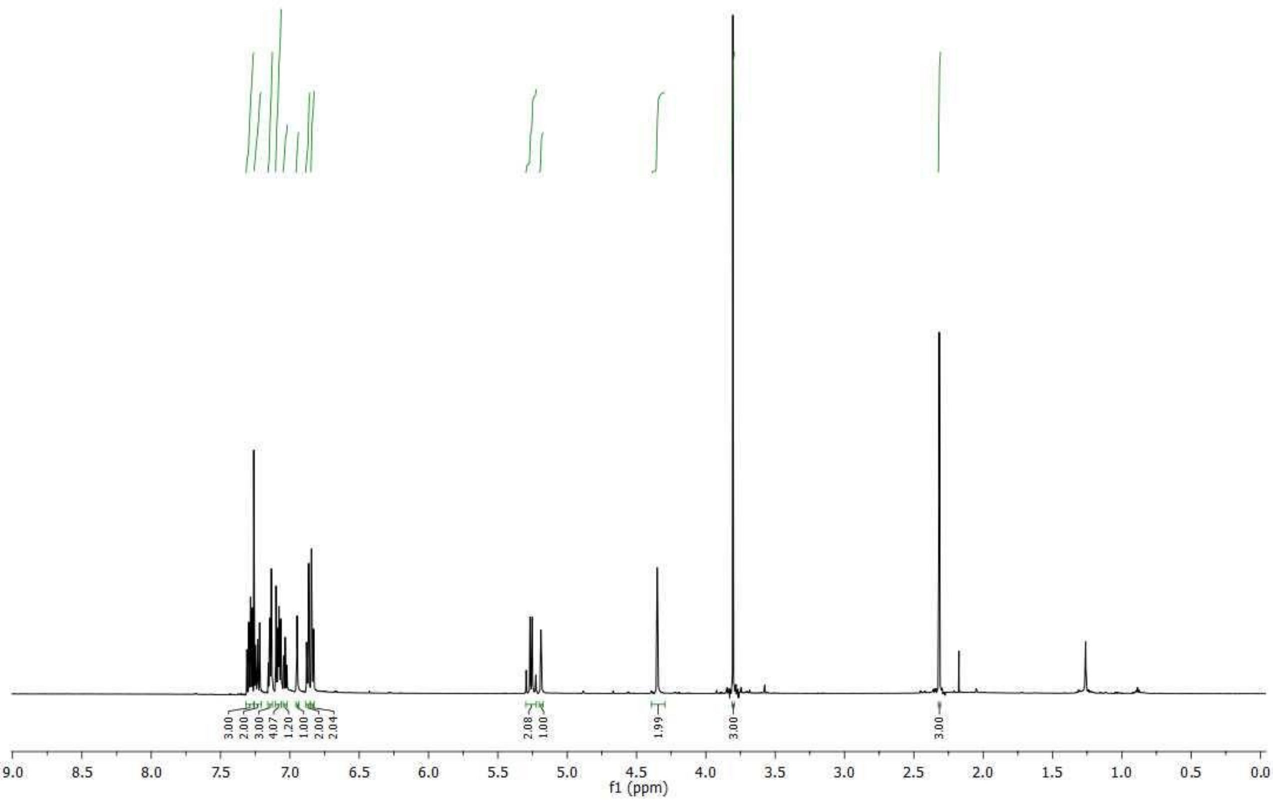

^{13}C NMR (150 MHz, $CDCl_3$)

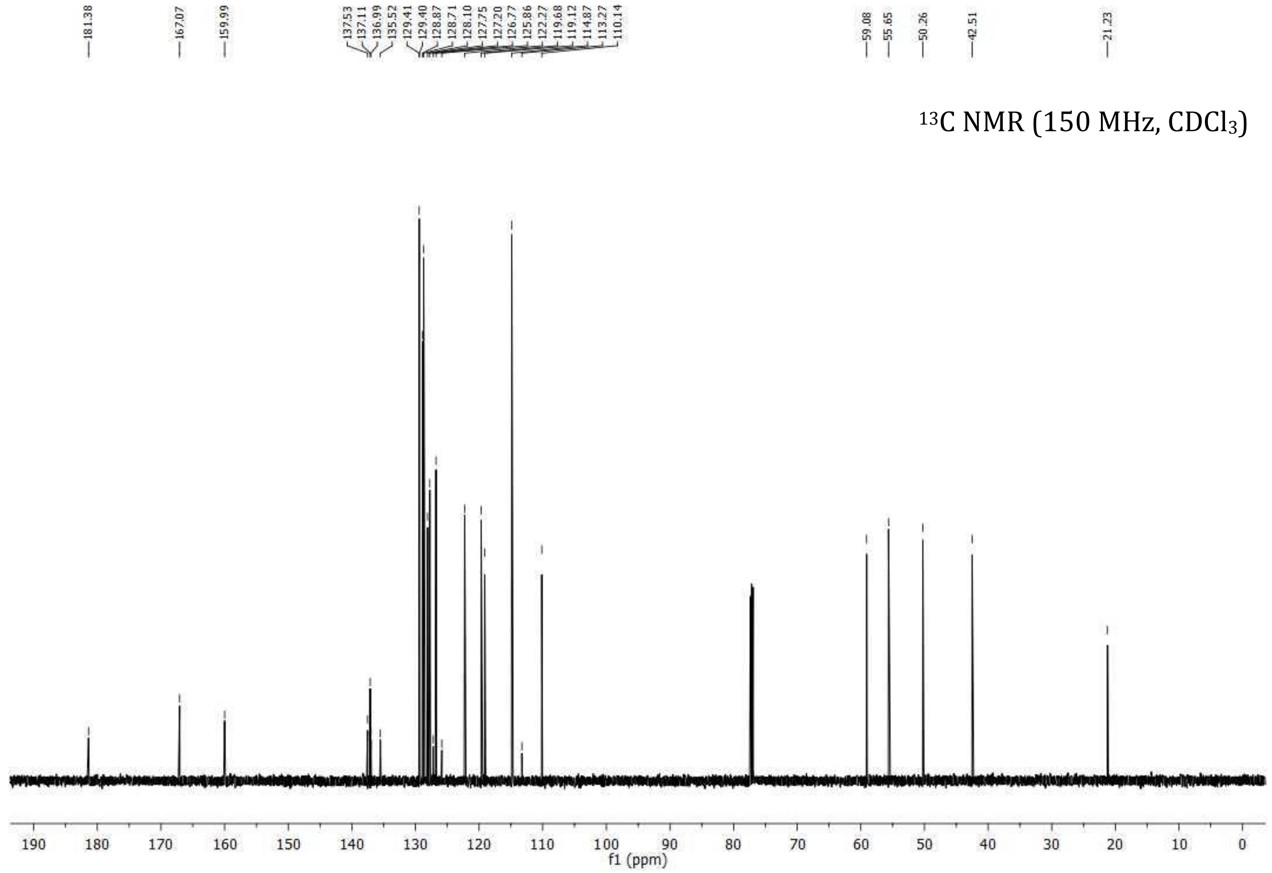

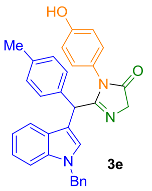

3e

^{1}H NMR (600 MHz, $CDCl_3$)

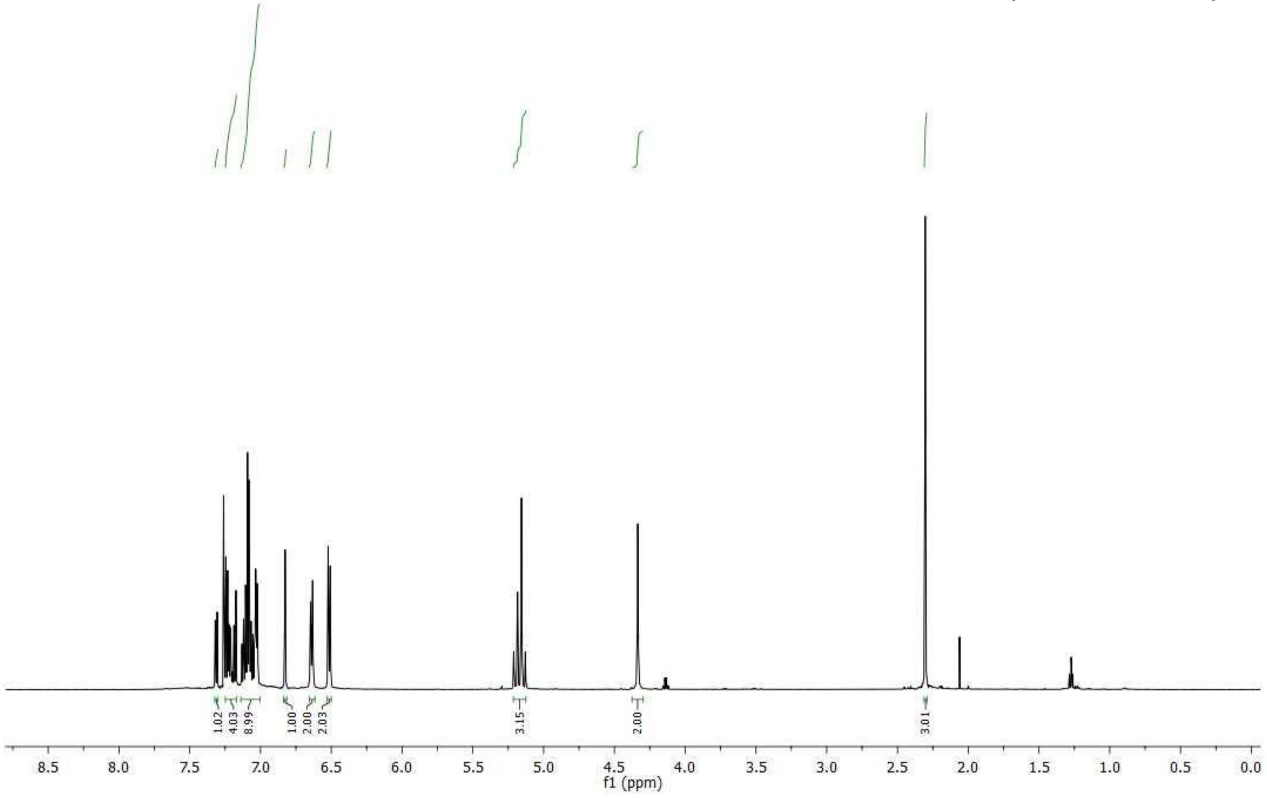

^{13}C NMR (150 MHz, $CDCl_3$)

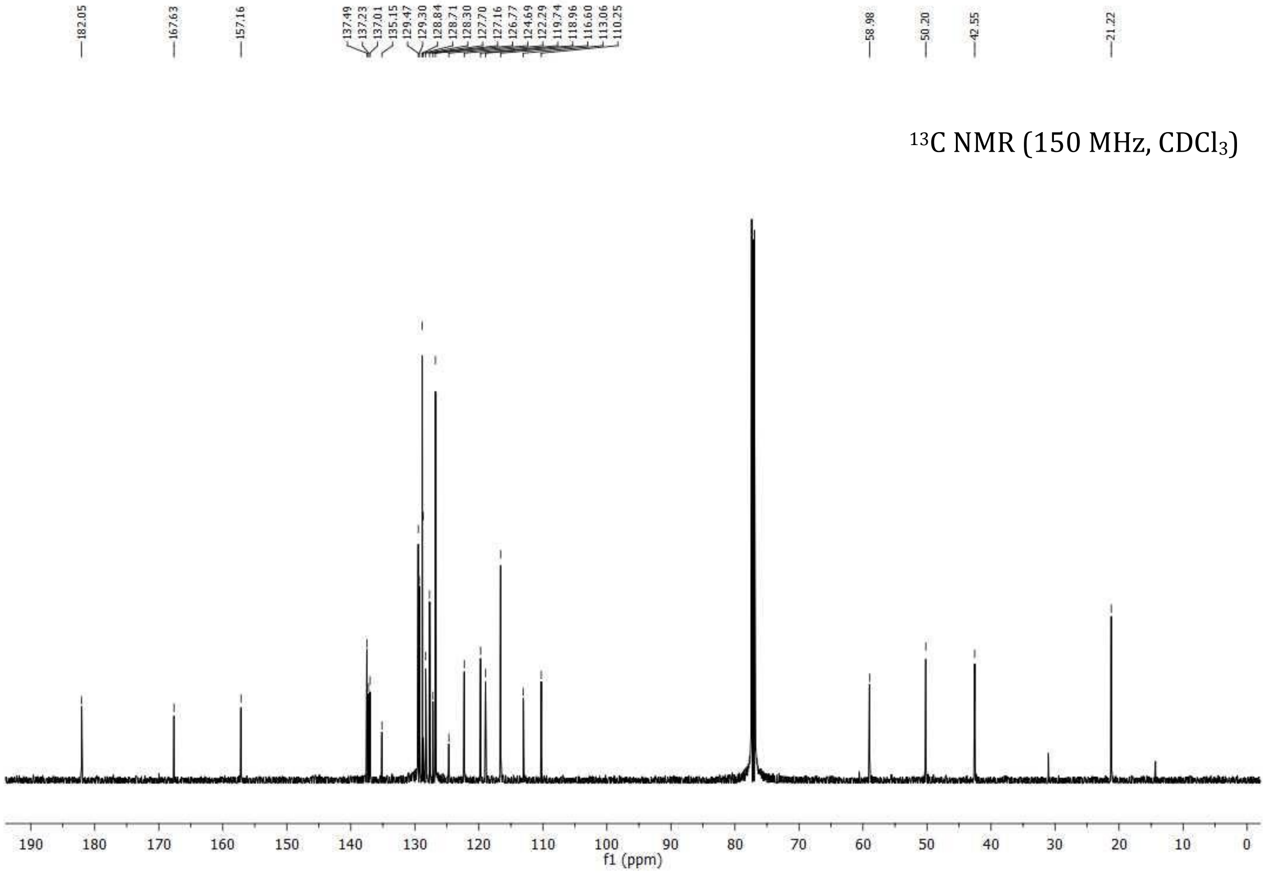

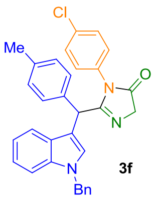

3f

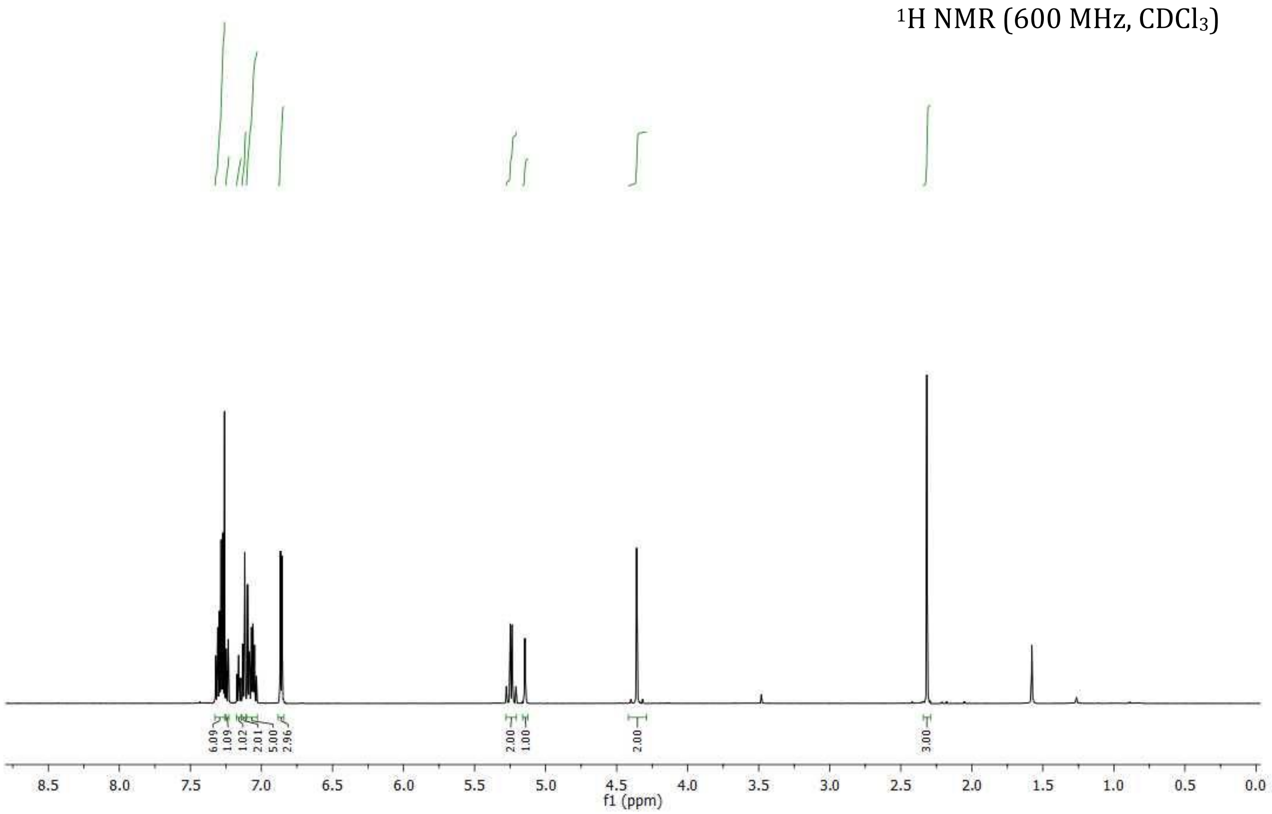

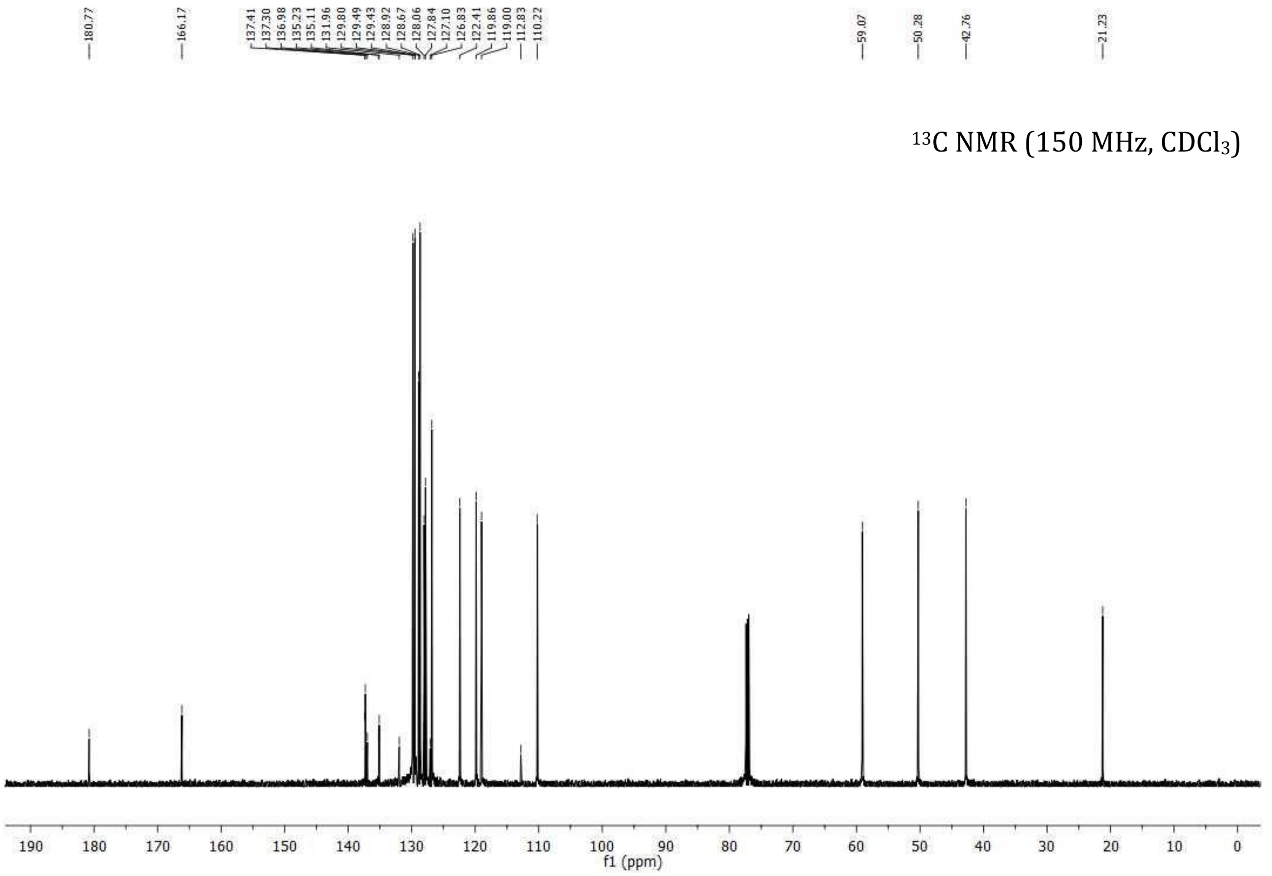

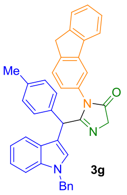

3g

^{1}H NMR (600 MHz, $CDCl_3$)

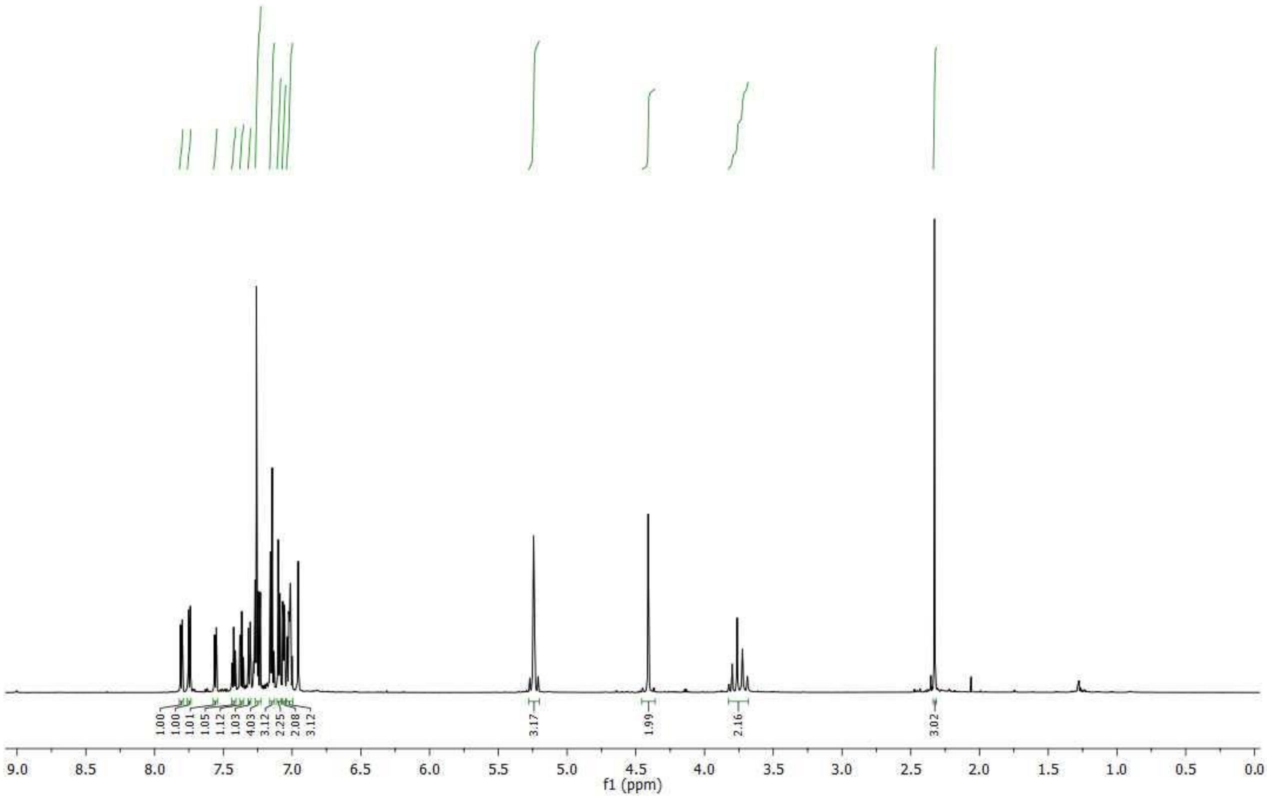

^{13}C NMR (150 MHz, $CDCl_3$)

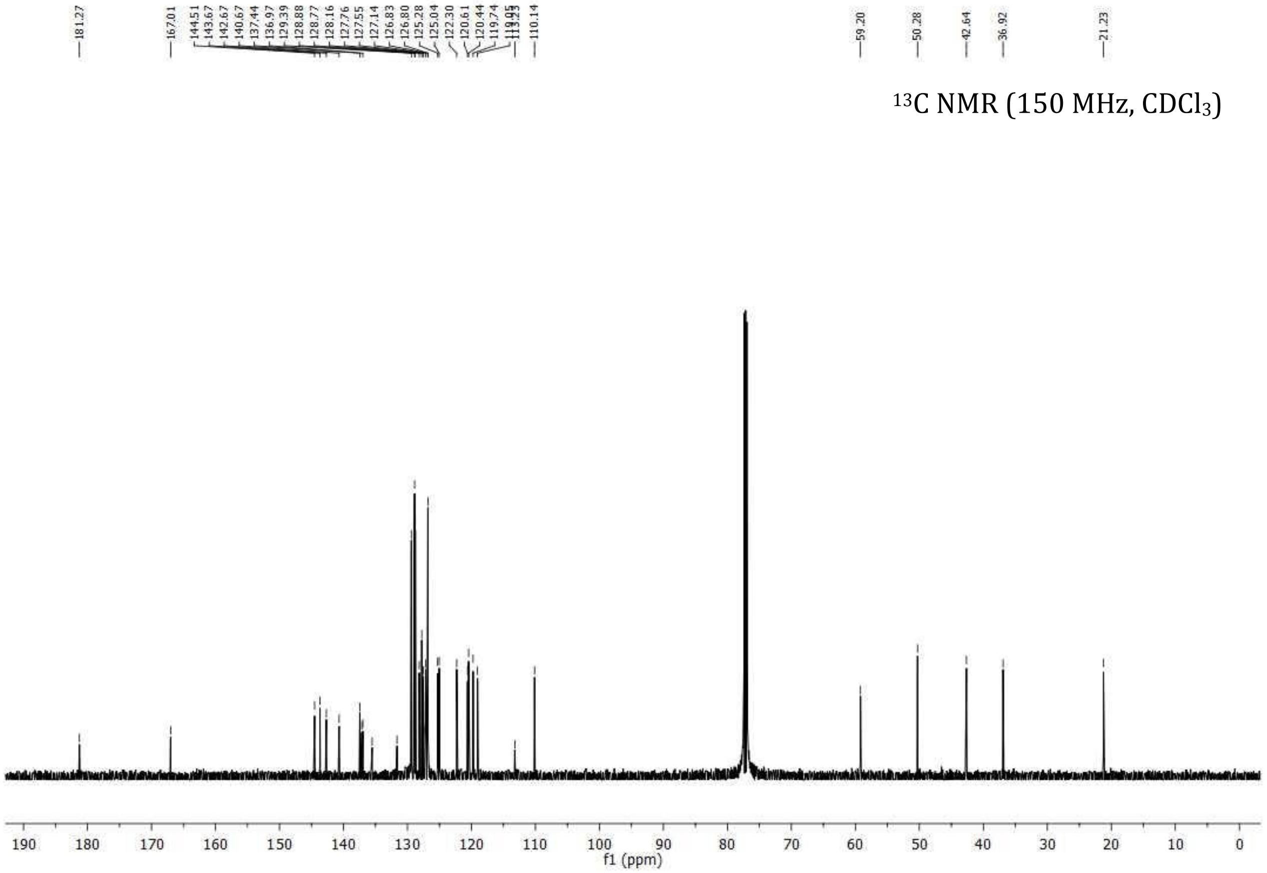

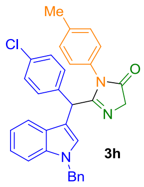

3h

^{1}H NMR (600 MHz, $CDCl_3$)

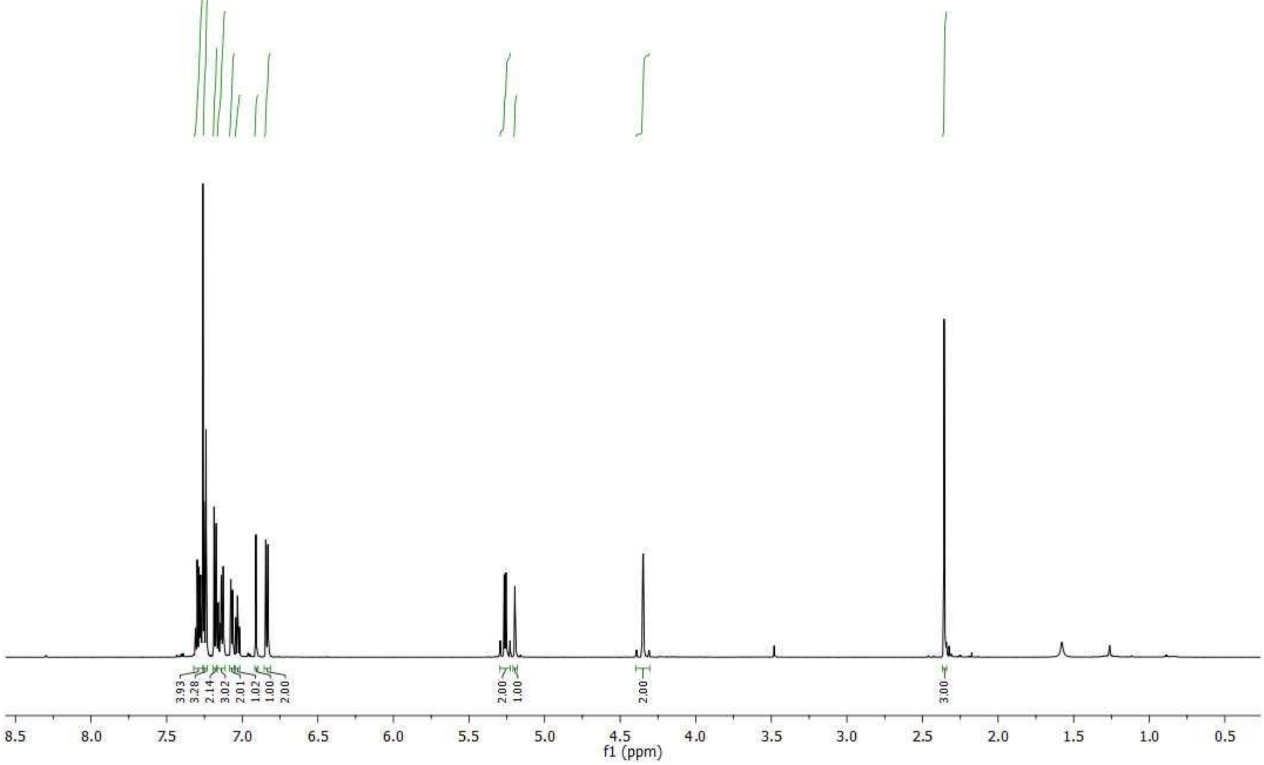

^{13}C NMR (150 MHz, $CDCl_3$)

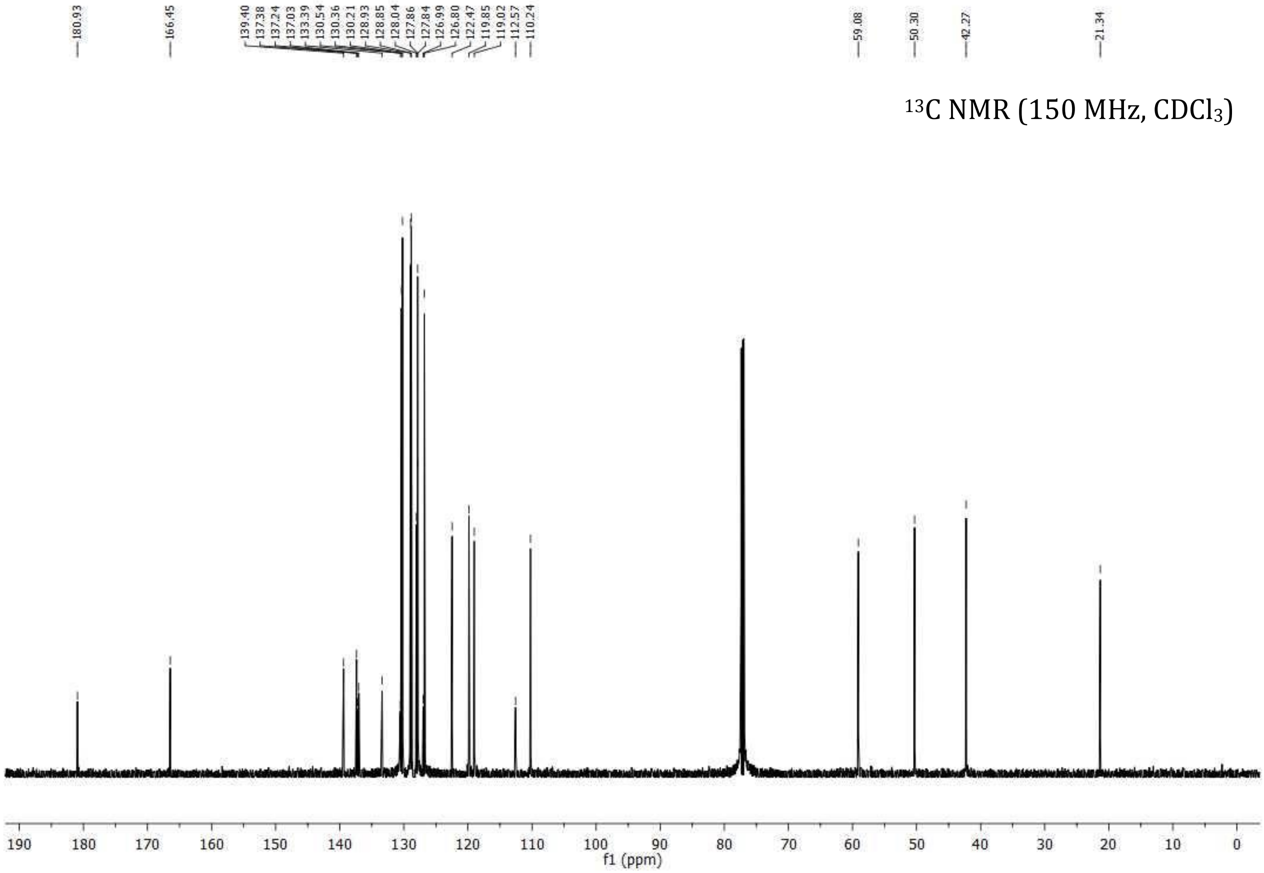

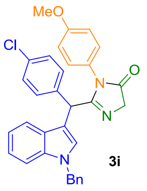

3i

^{1}H NMR (600 MHz, $CDCl_3$)

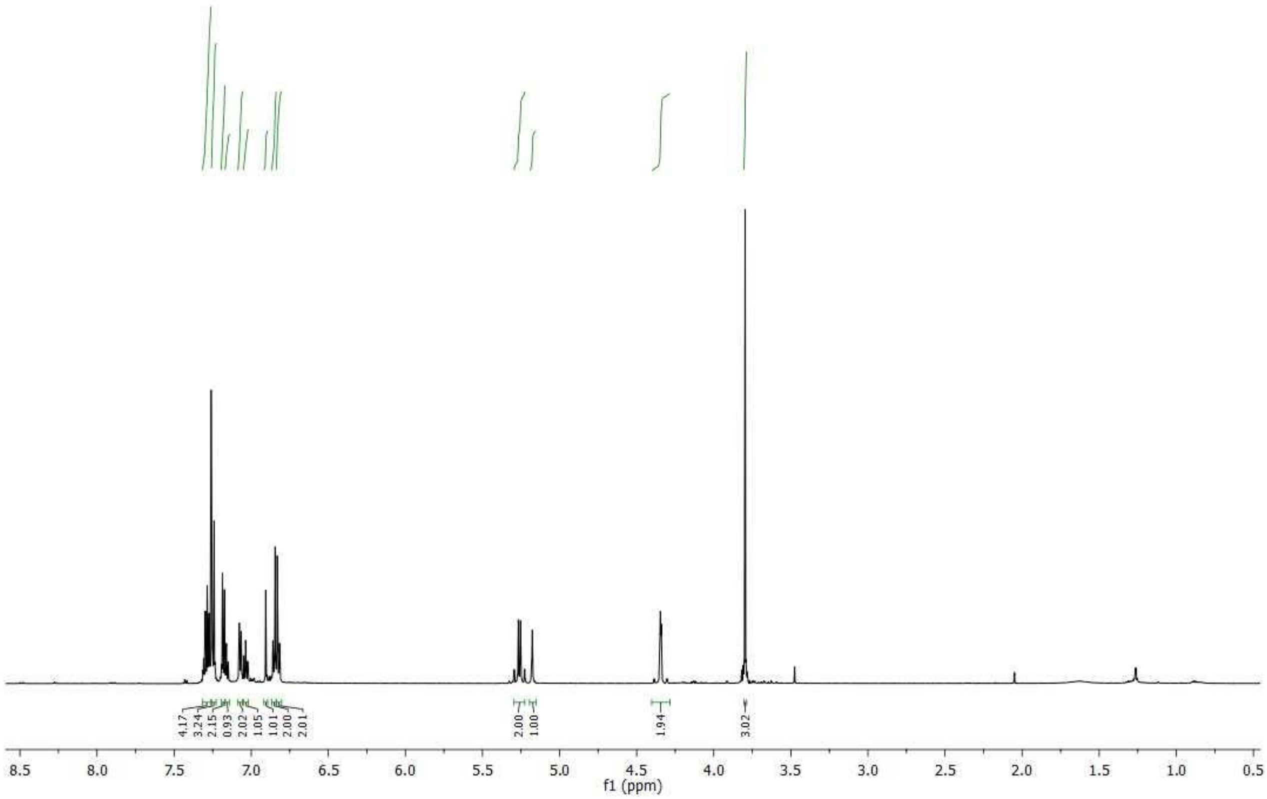

^{13}C NMR (150 MHz, $CDCl_3$)

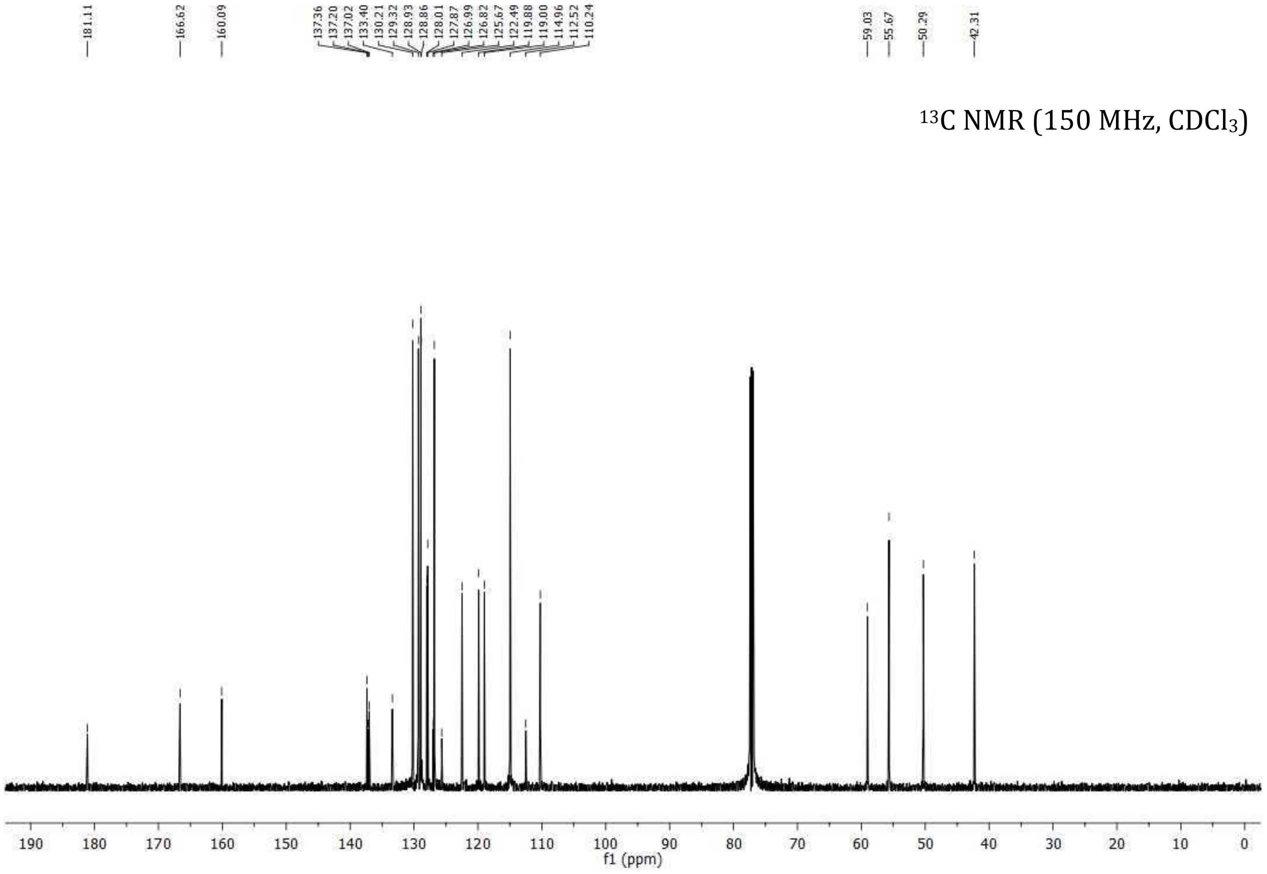

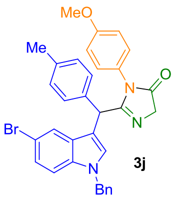

3j

^{1}H NMR (600 MHz, $CDCl_3$)

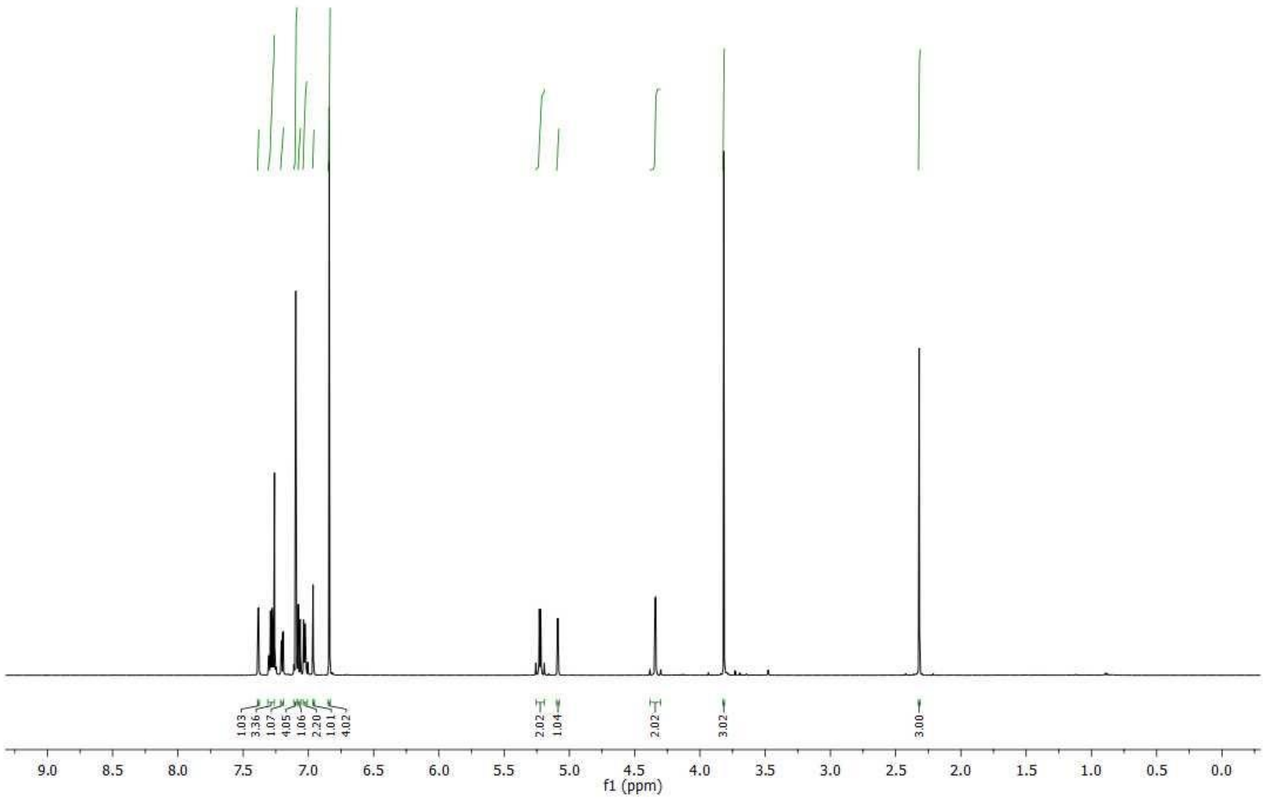

^{13}C NMR (150 MHz, $CDCl_3$)

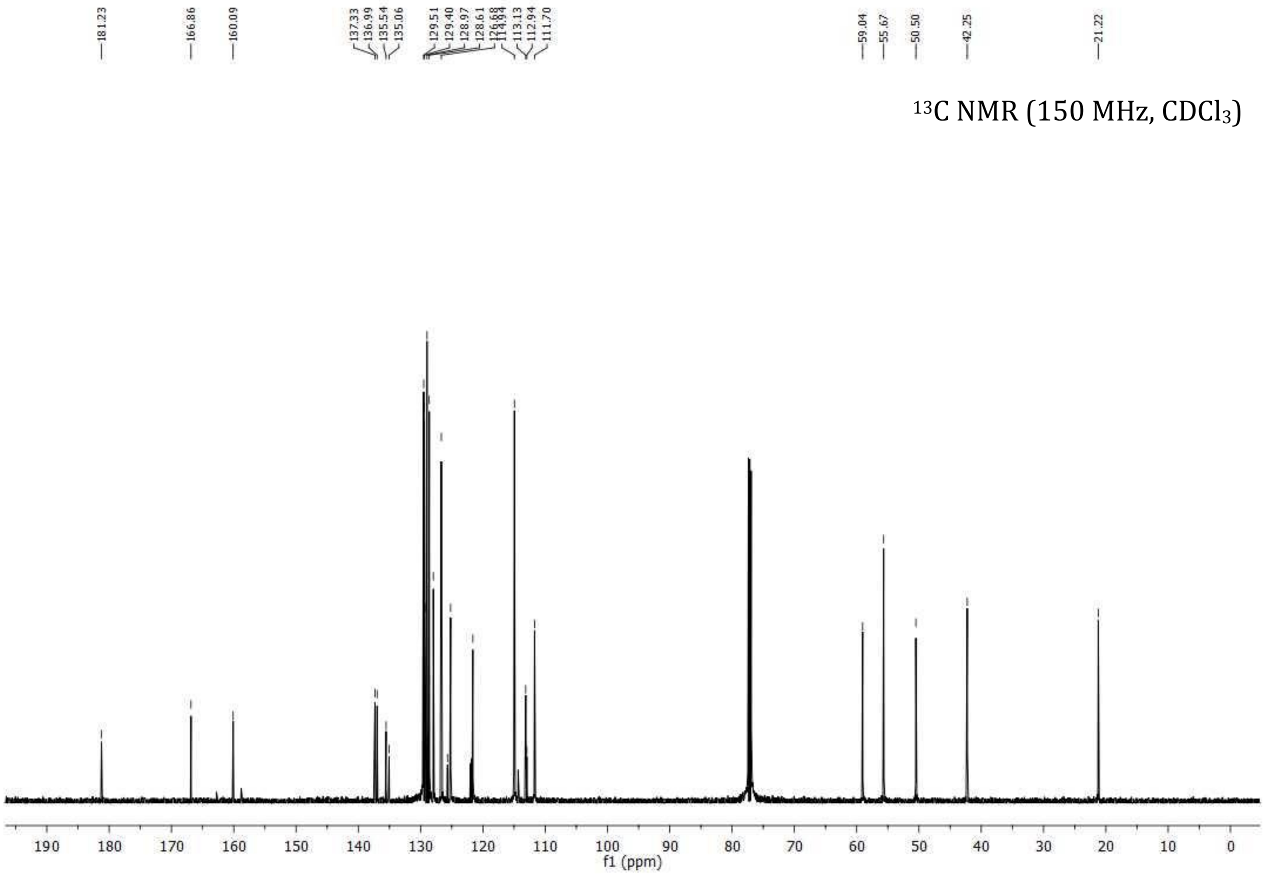

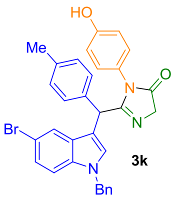

3k

^{1}H NMR (600 MHz, $CDCl_3$)

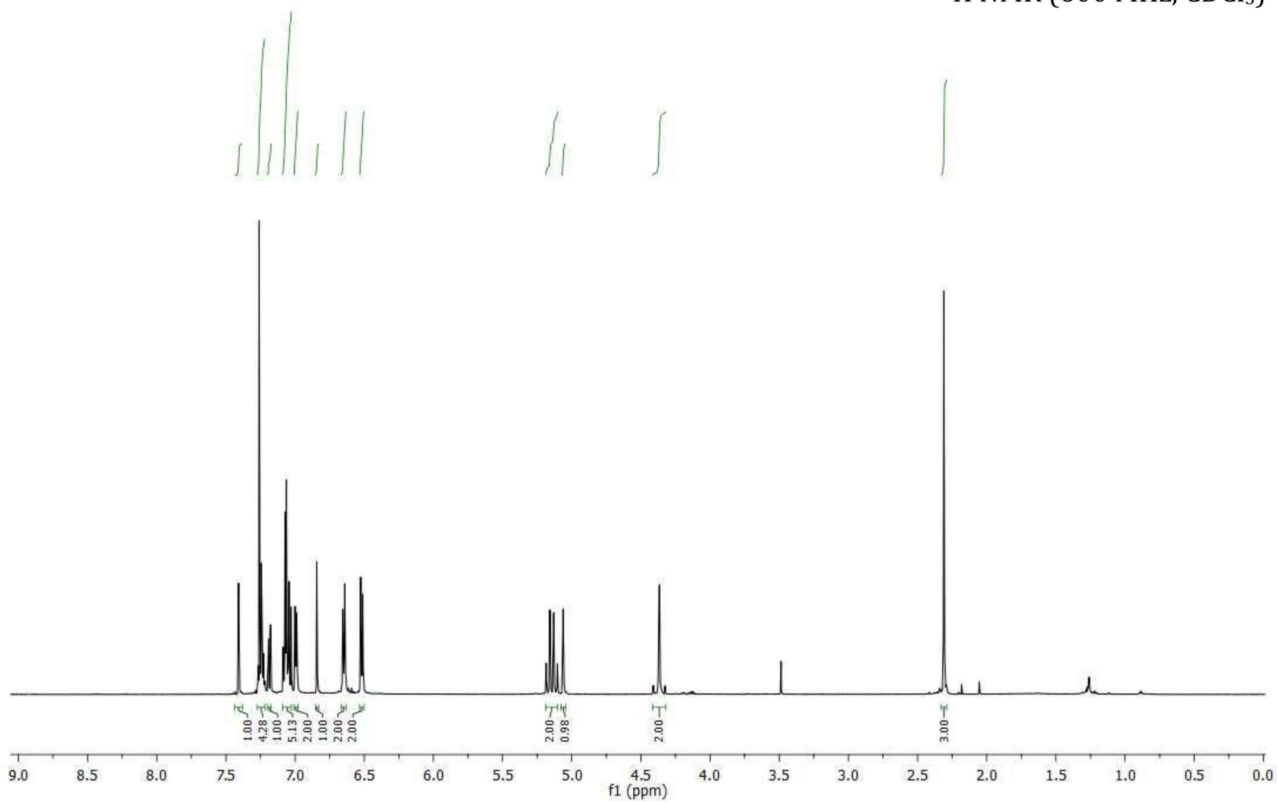

^{13}C NMR (150 MHz, $CDCl_3$)

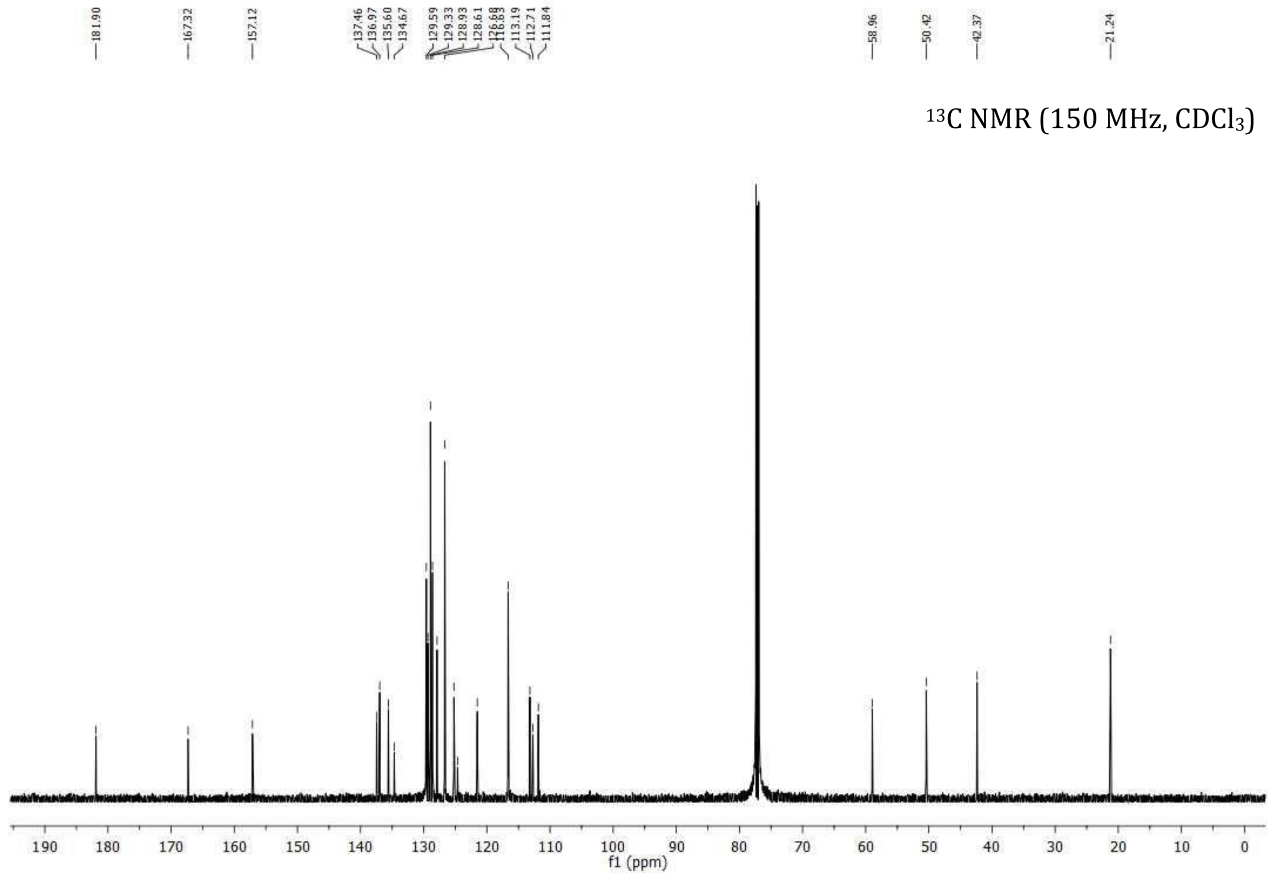

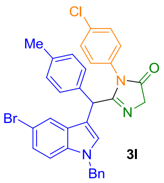

3l

¹H NMR (600 MHz, $CDCl_3$)

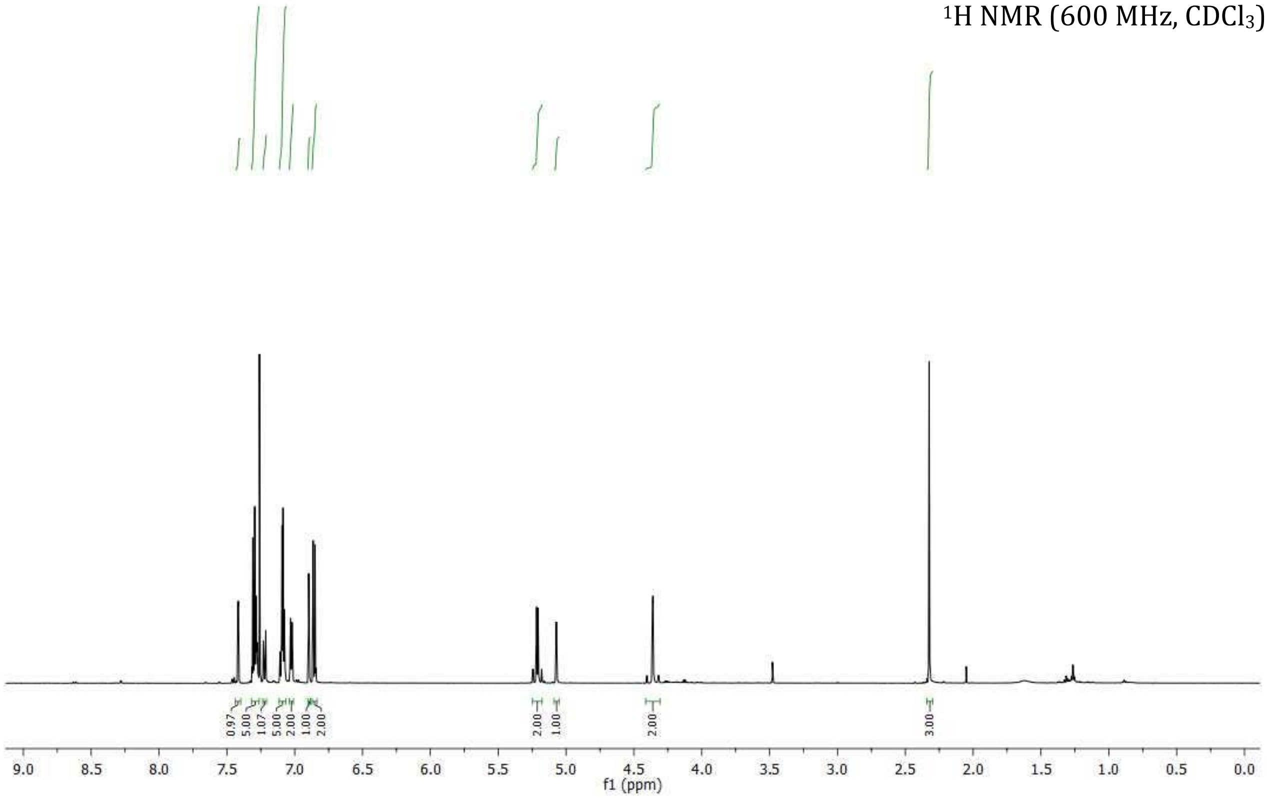

¹³C NMR (150 MHz, $CDCl_3$)

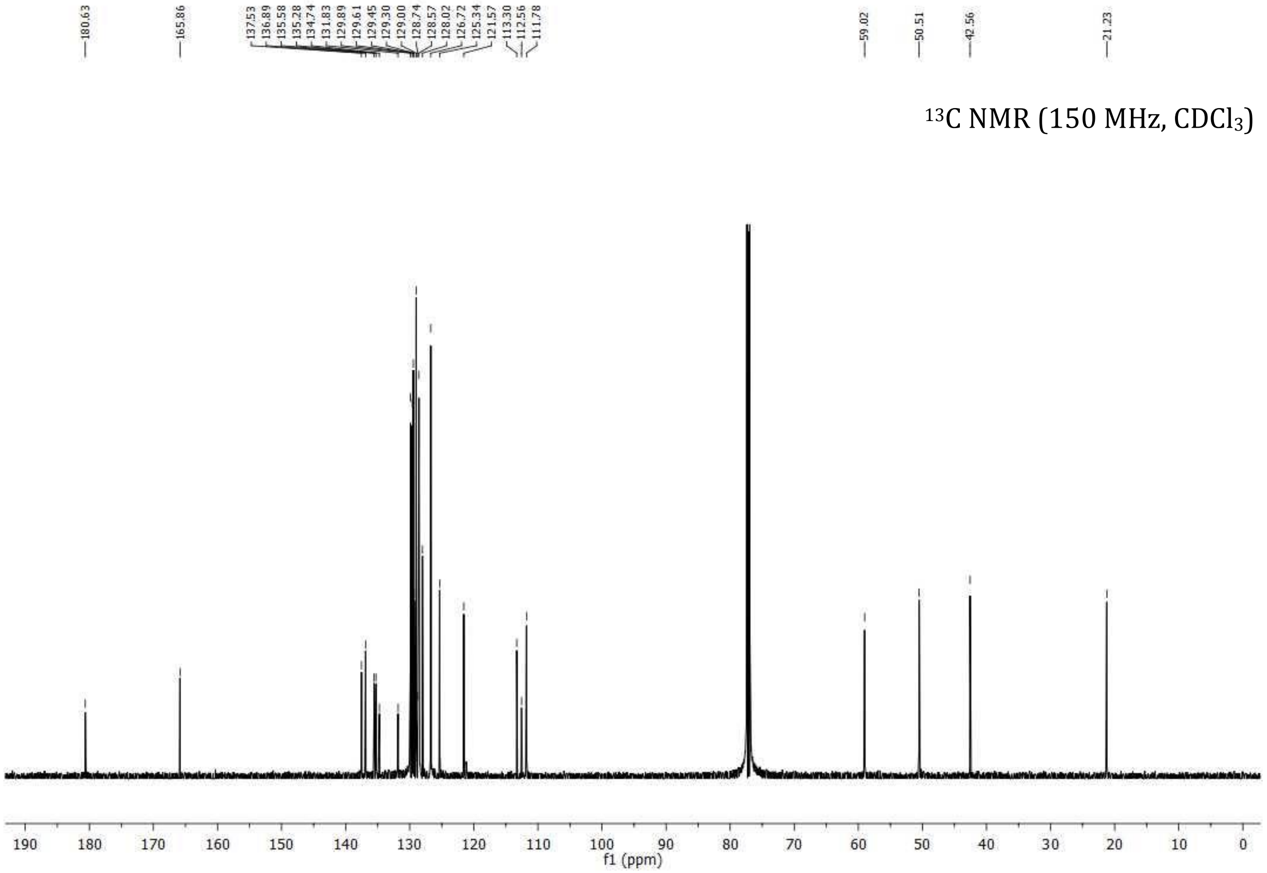

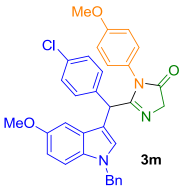

3m

^{1}H NMR (600 MHz, $CDCl_3$)

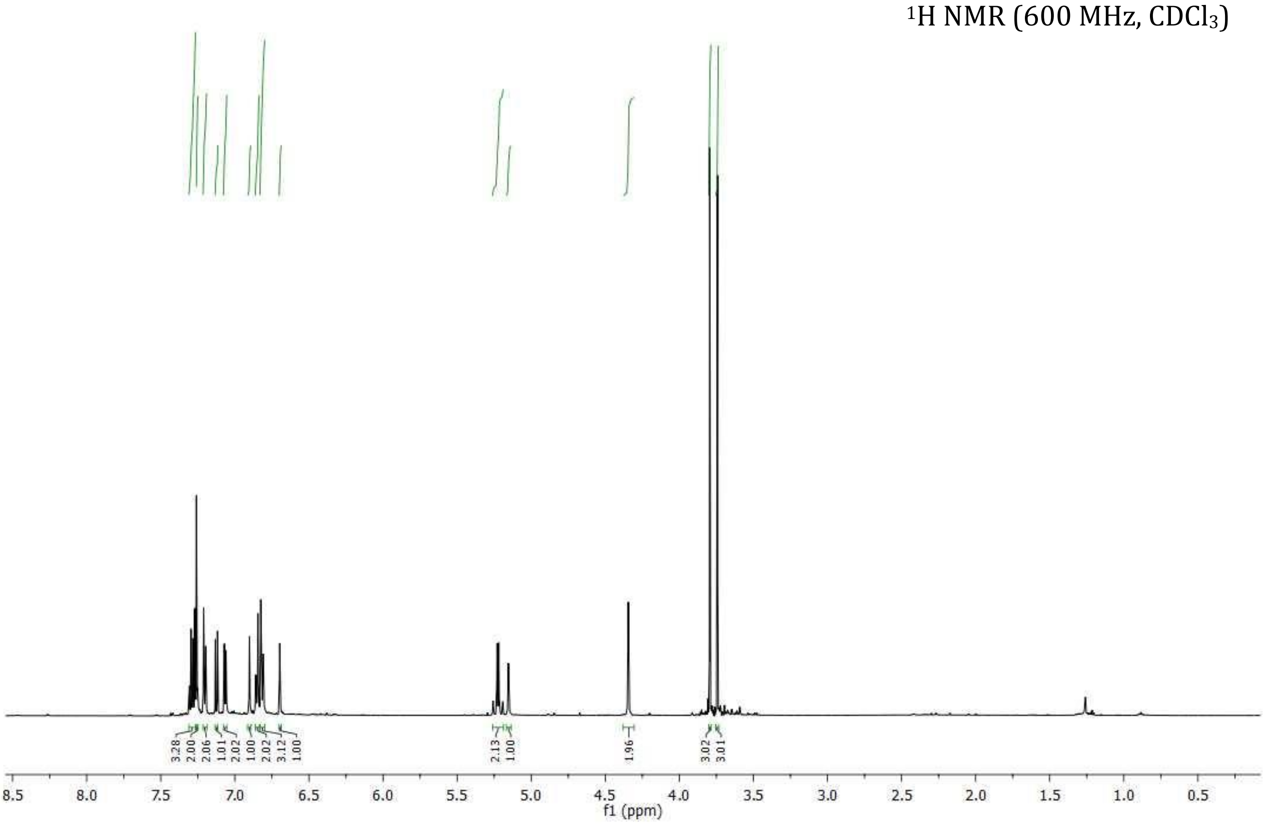

^{13}C NMR (150 MHz, $CDCl_3$)

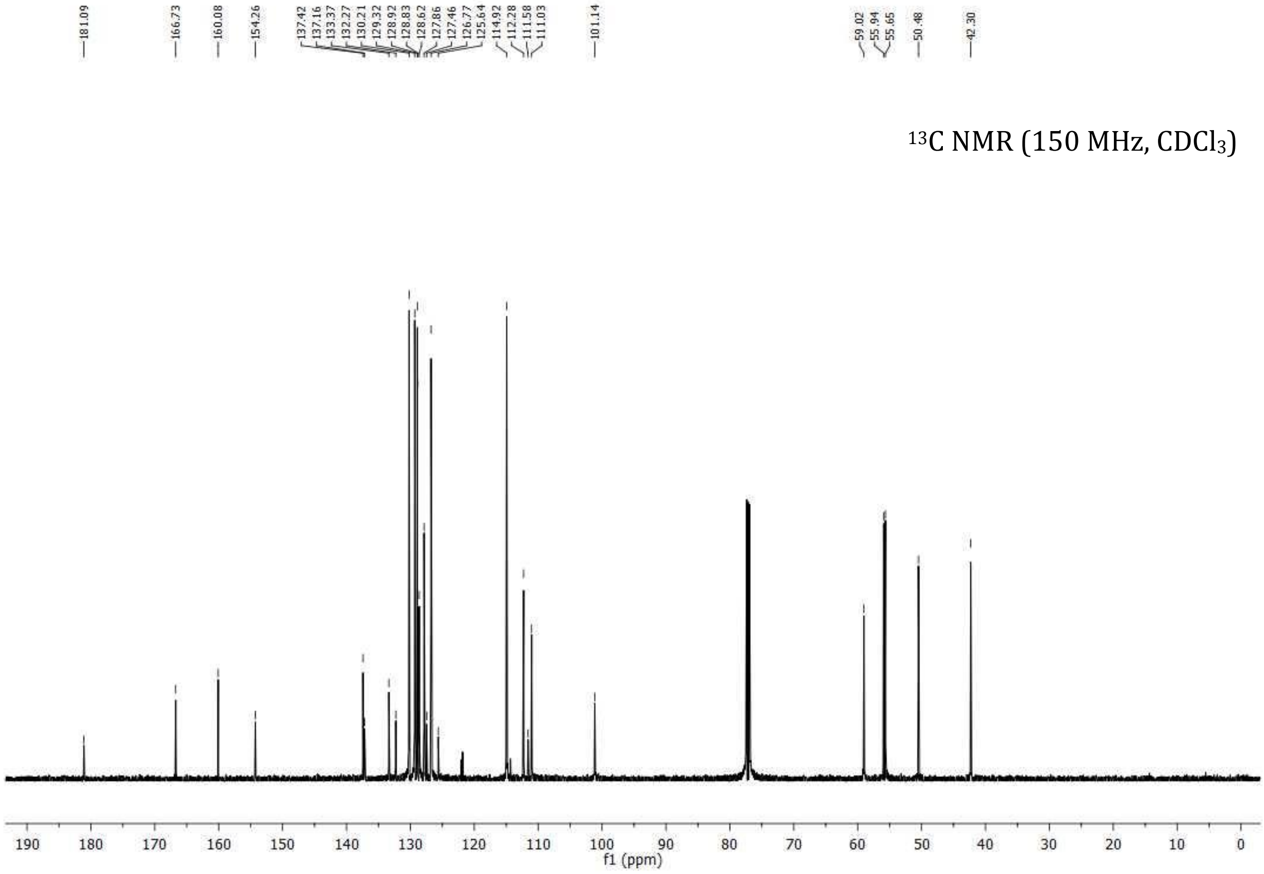

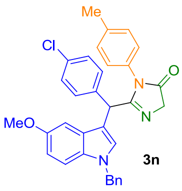
Me
Cl
N
O
N
MeO
N
Bn

3n

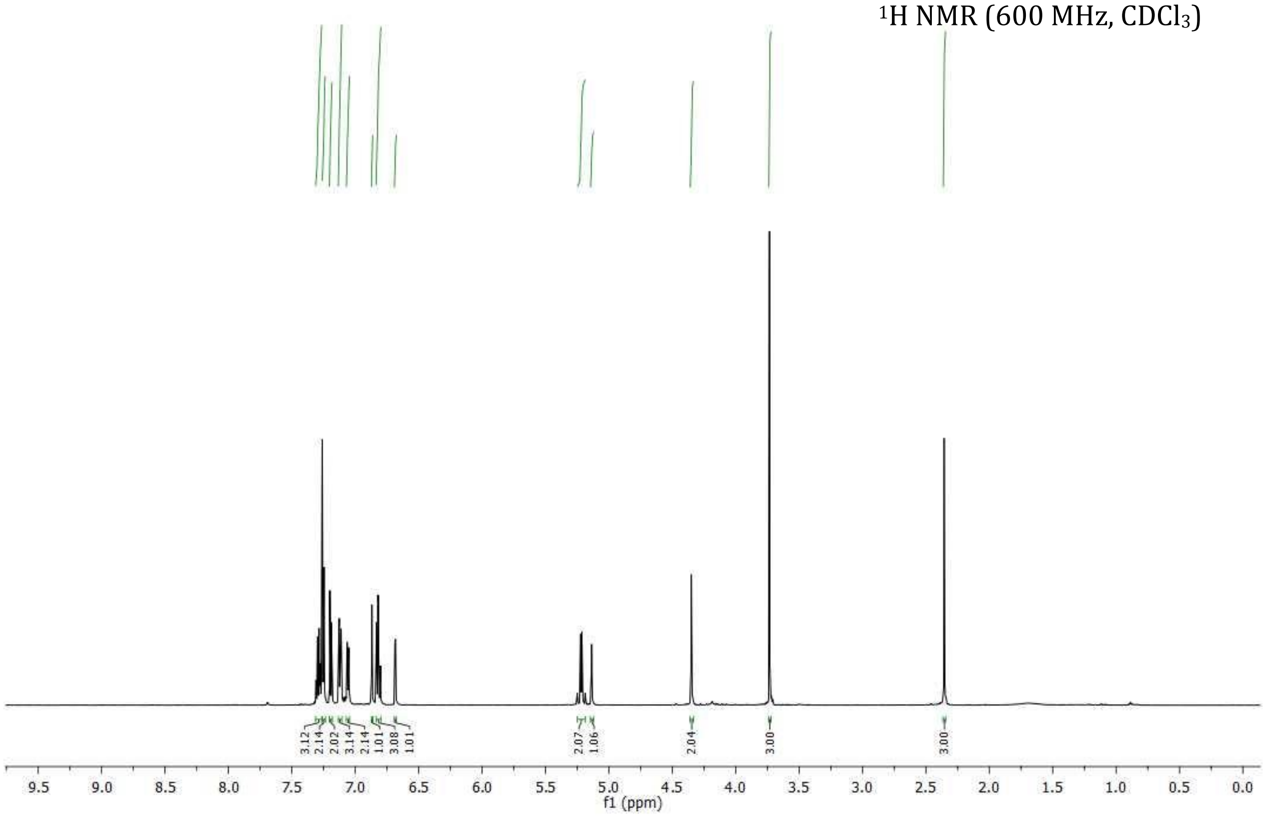
1H NMR (600 MHz, CDCl3)
f1 (ppm)

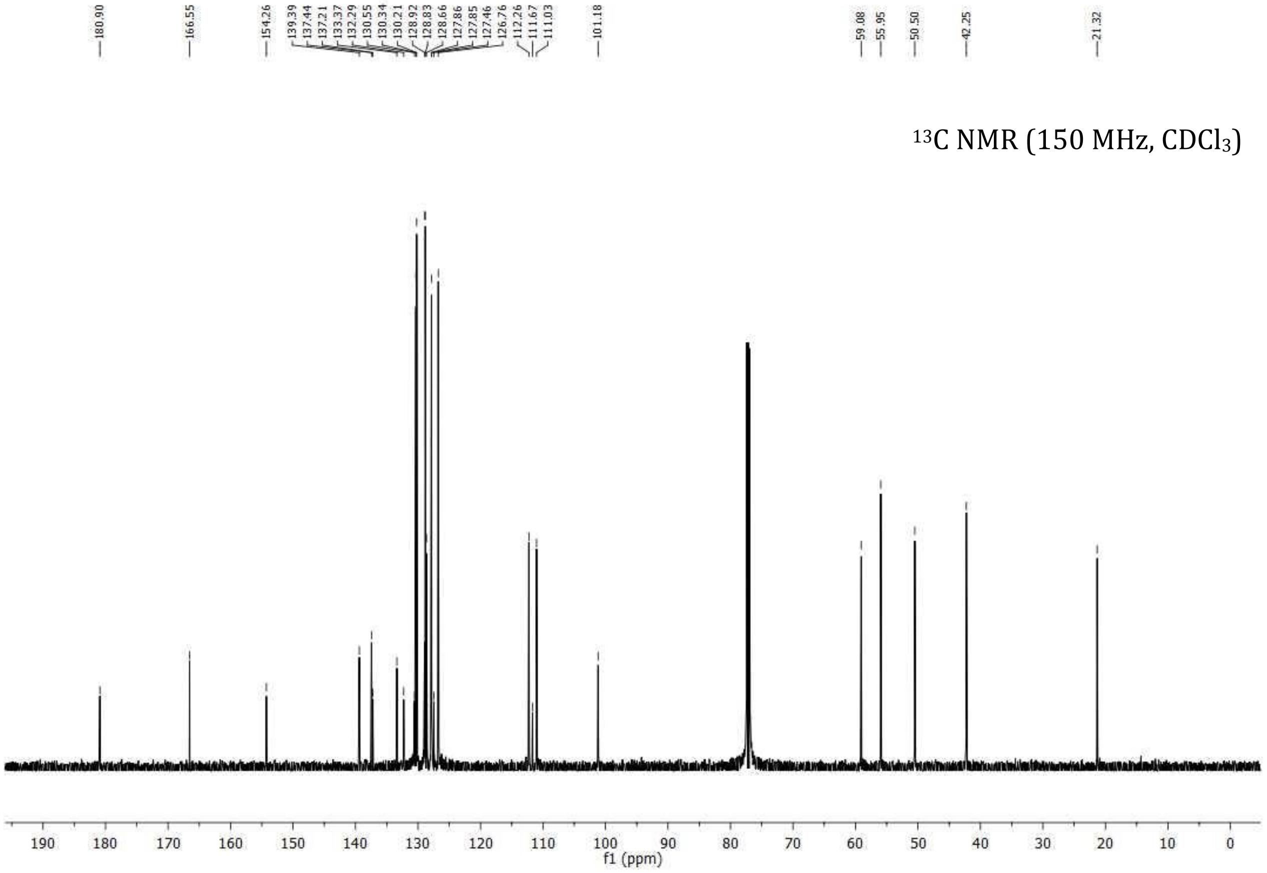
13C NMR (150 MHz, CDCl3)
180.90
166.55
154.26
139.39
137.44
137.21
133.37
132.29
130.55
130.34
130.21
128.92
128.83
128.66
127.86
127.85
127.46
126.76
112.26
111.67
111.03
101.18
59.08
55.95
50.50
42.25
21.32
f1 (ppm)

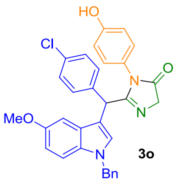

3o

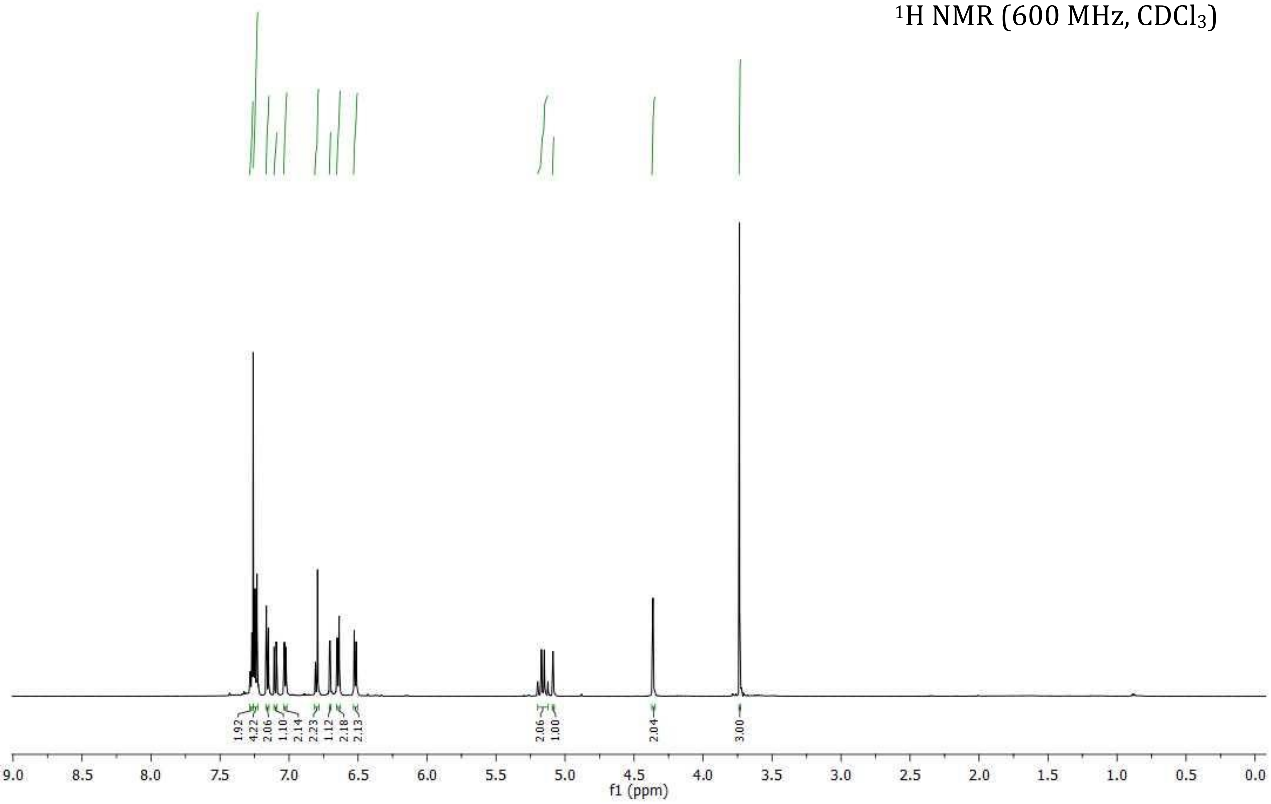

^{1}H NMR (600 MHz, $CDCl_3$)

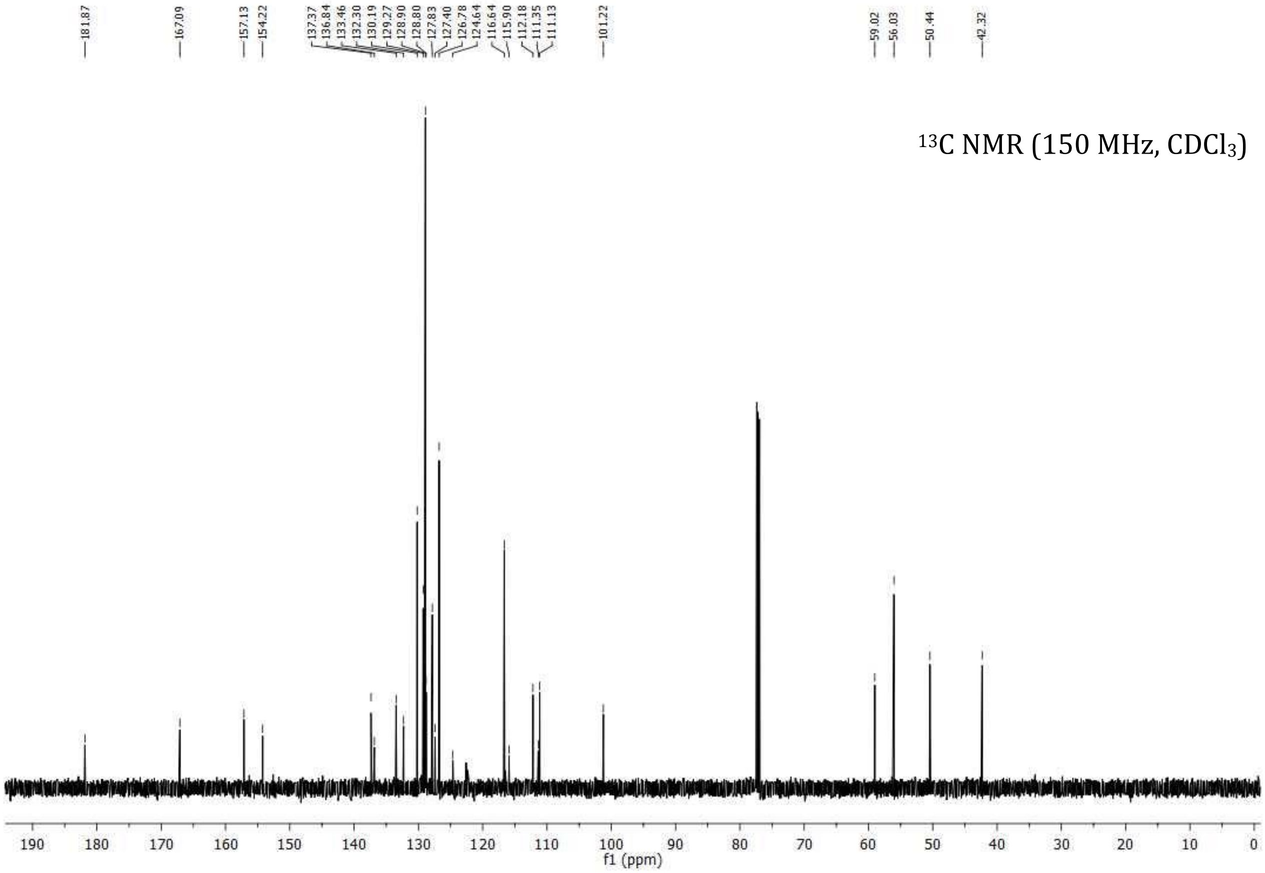

^{13}C NMR (150 MHz, $CDCl_3$)

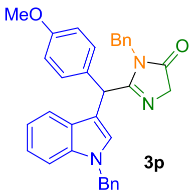

3p

^{1}H NMR (600 MHz, $CDCl_3$)

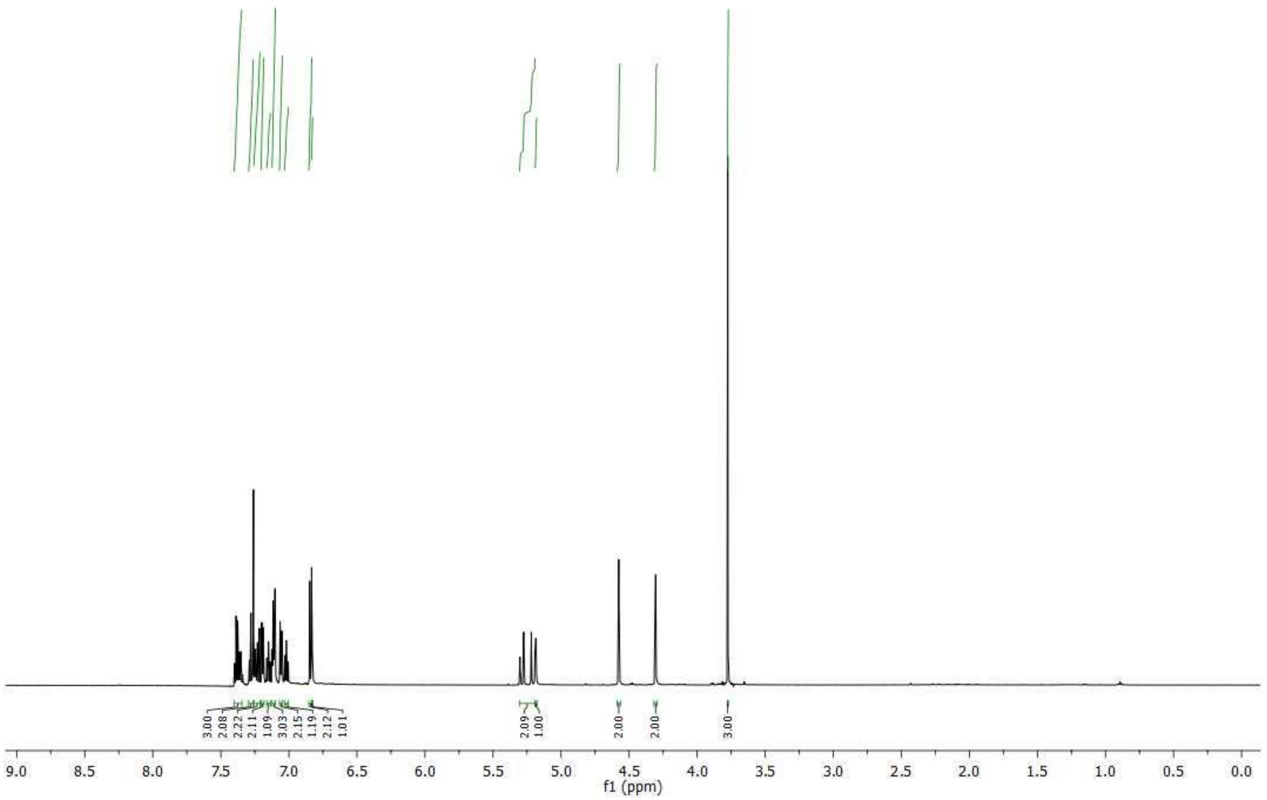

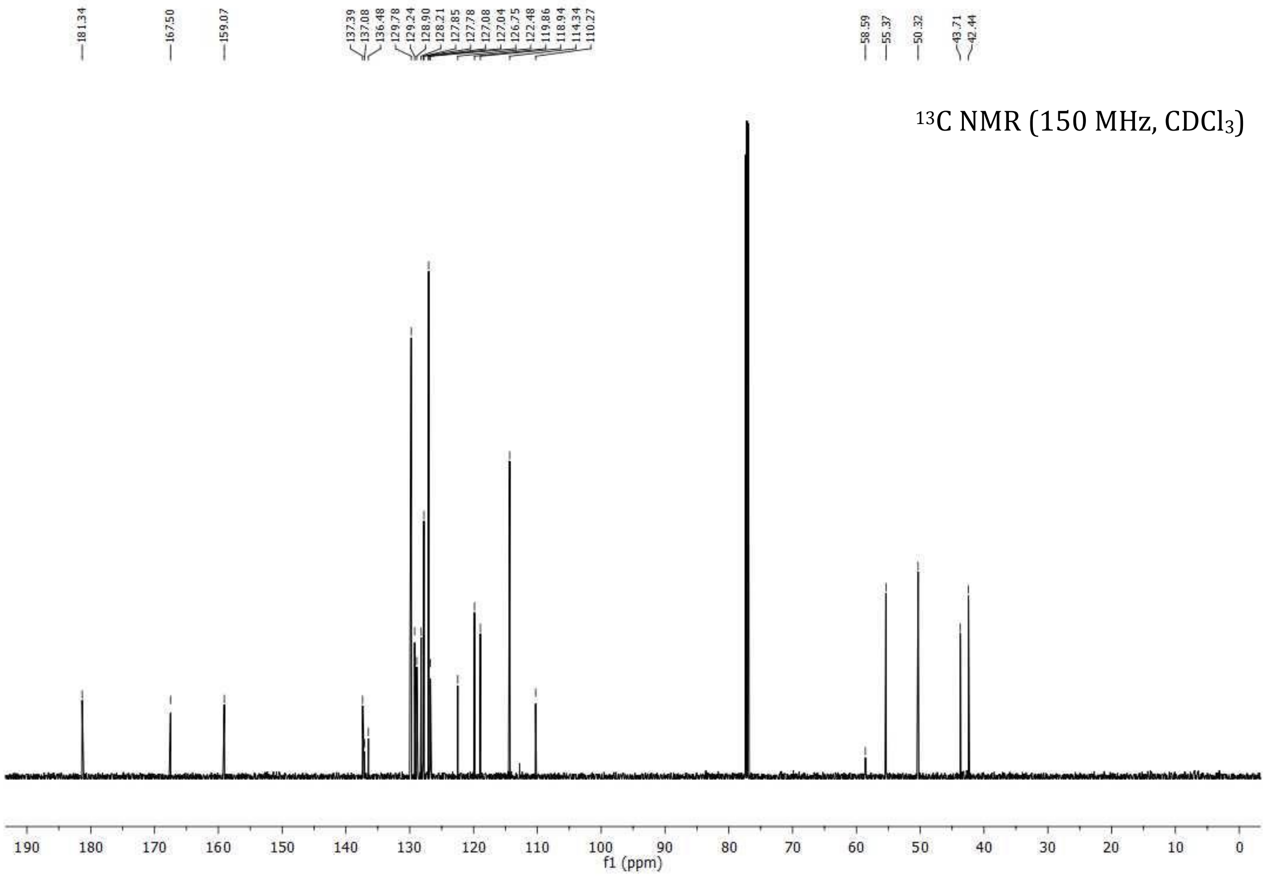

^{13}C NMR (150 MHz, $CDCl_3$)

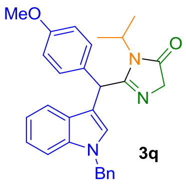

3q

^{1}H NMR (600 MHz, CDCl$_3$)

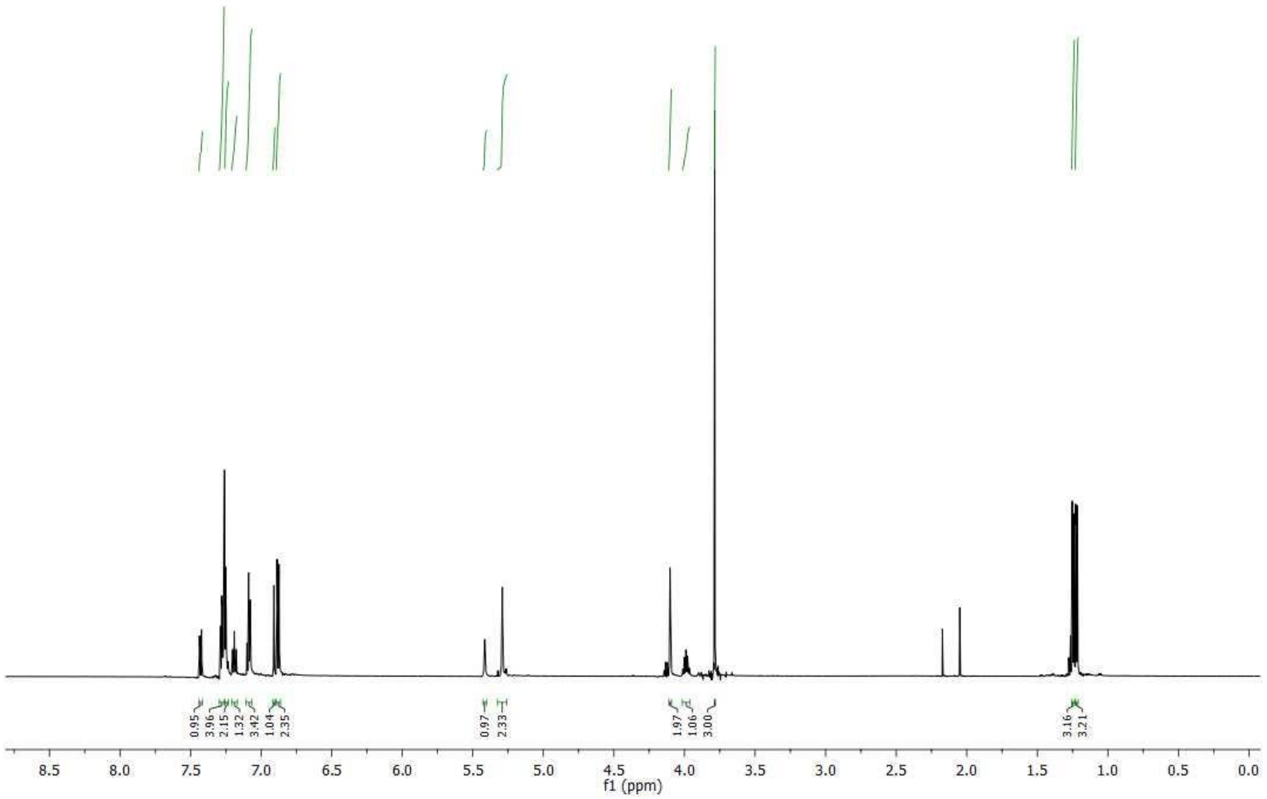

^{13}C NMR (150 MHz, CDCl$_3$)

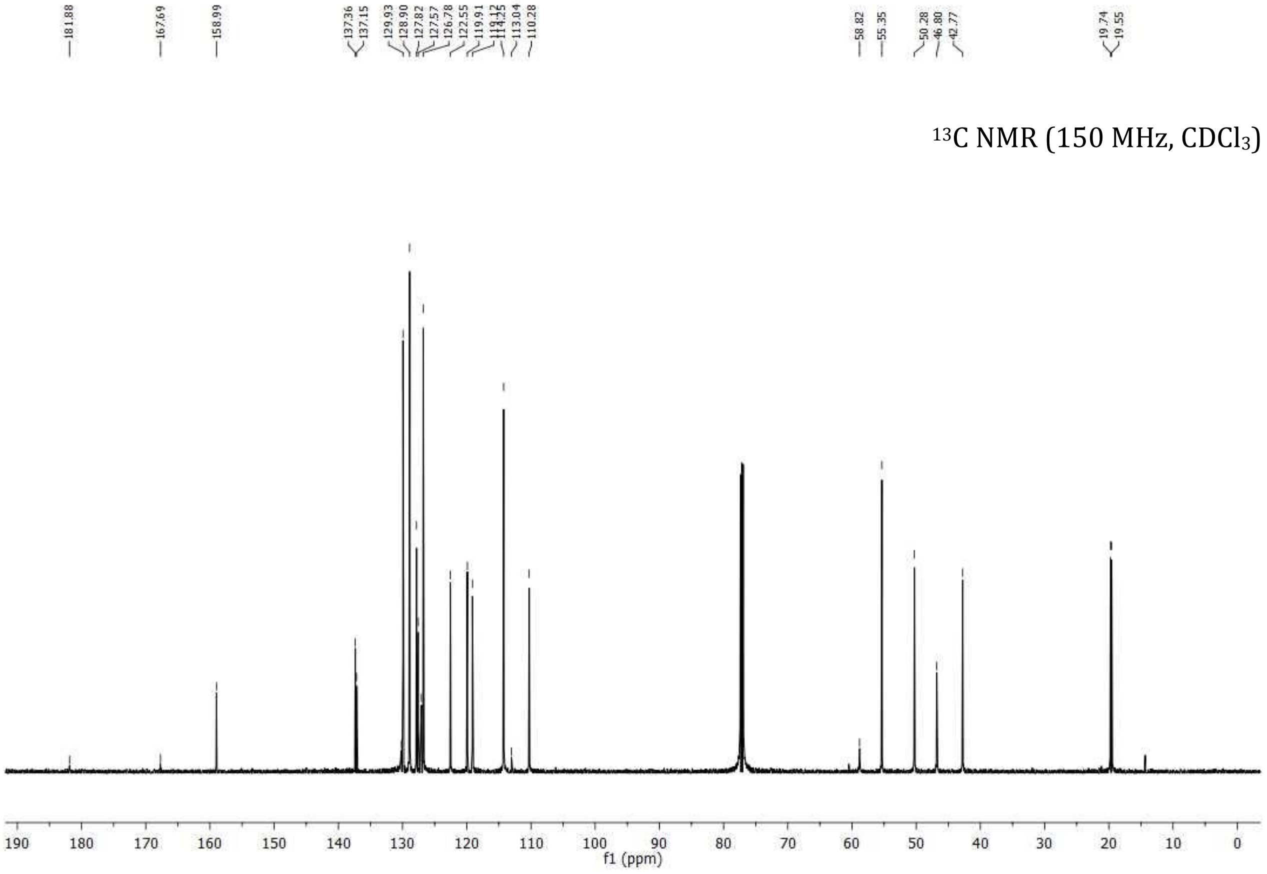

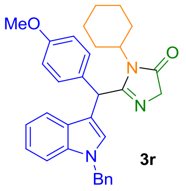
MeO
N
O
N
N
Bn

3r

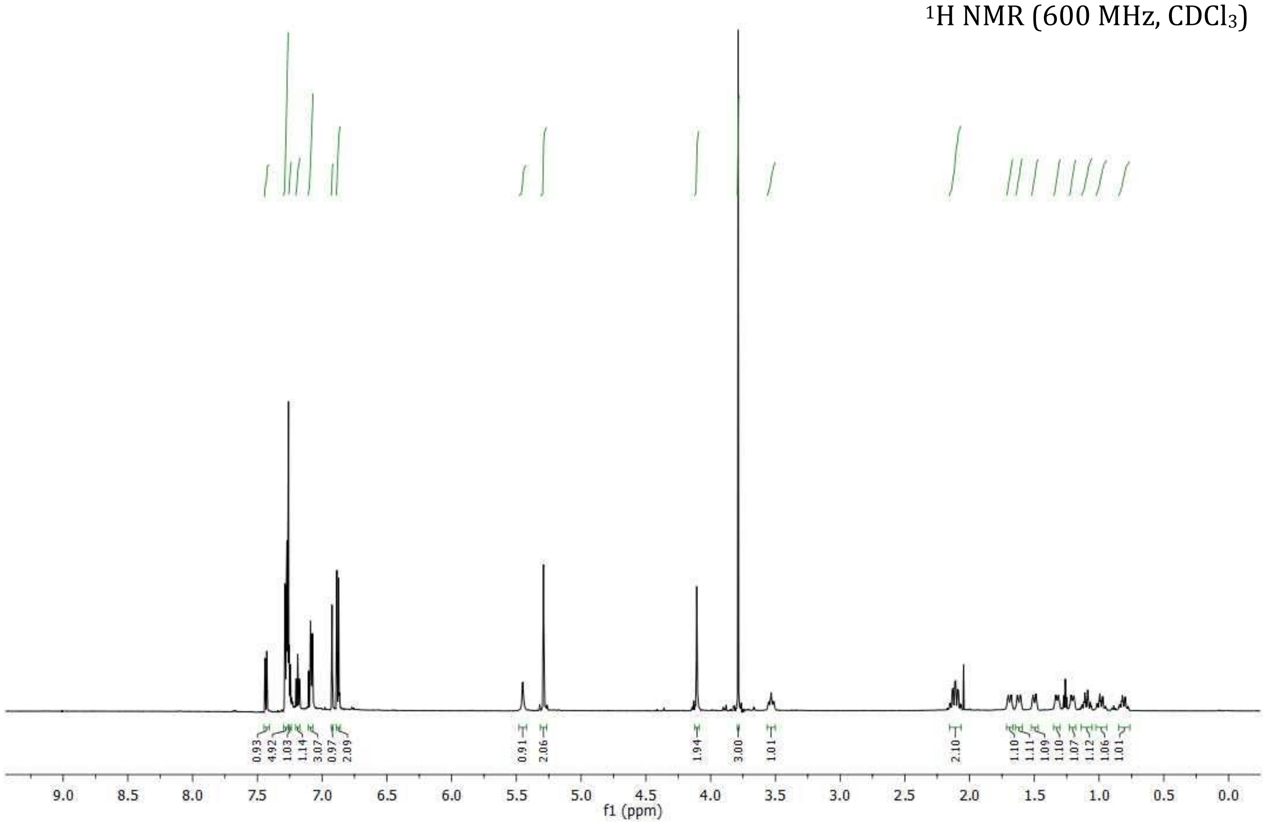
1H NMR (600 MHz, CDCl3)
f1 (ppm)

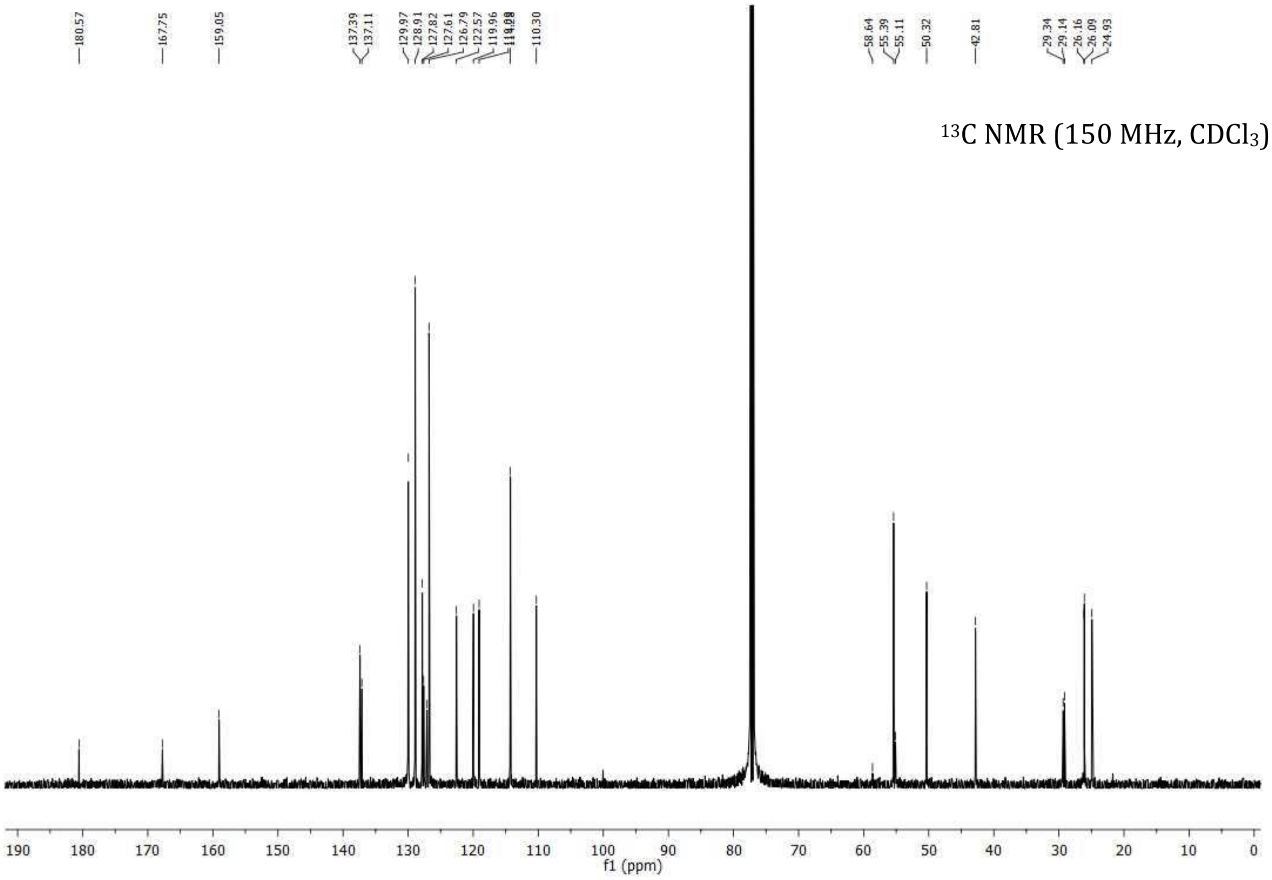
13C NMR (150 MHz, CDCl3)
f1 (ppm)

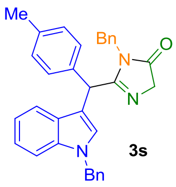

3s

^{1}H NMR (600 MHz, $CDCl_3$)

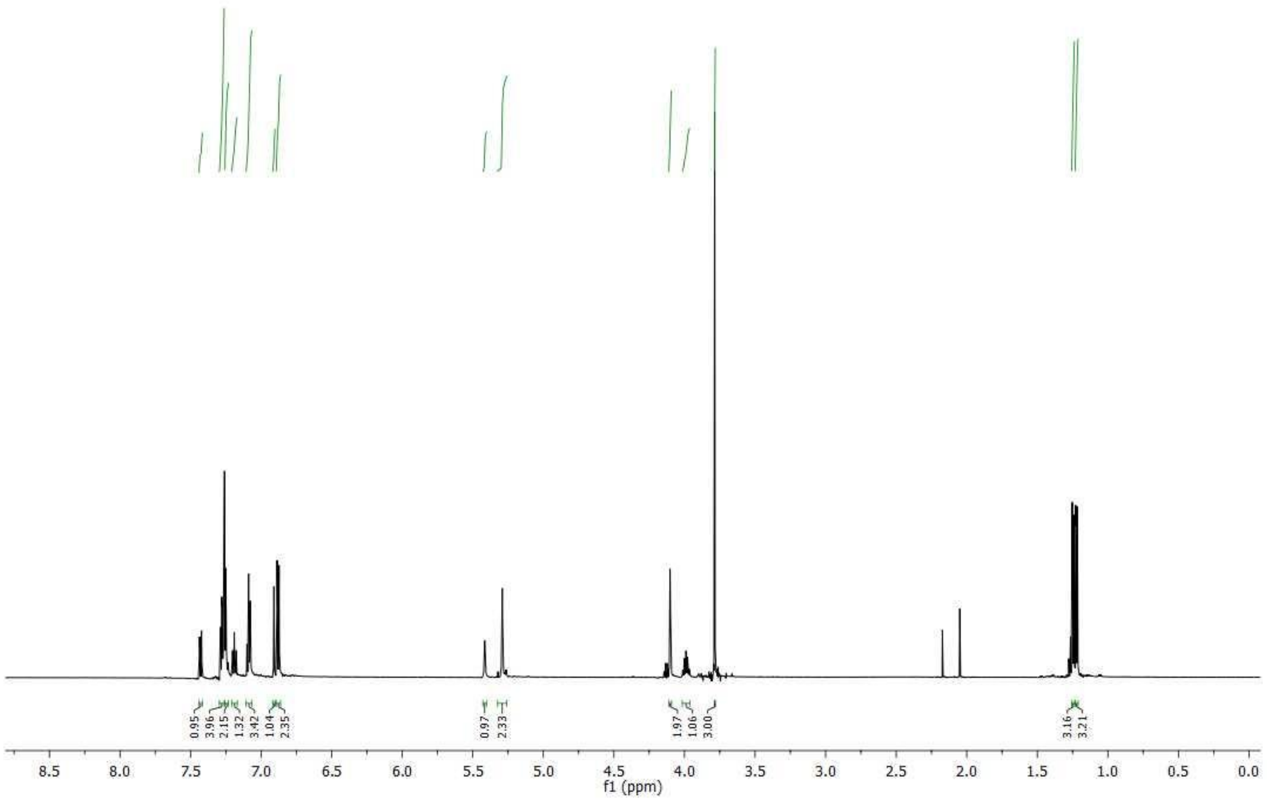

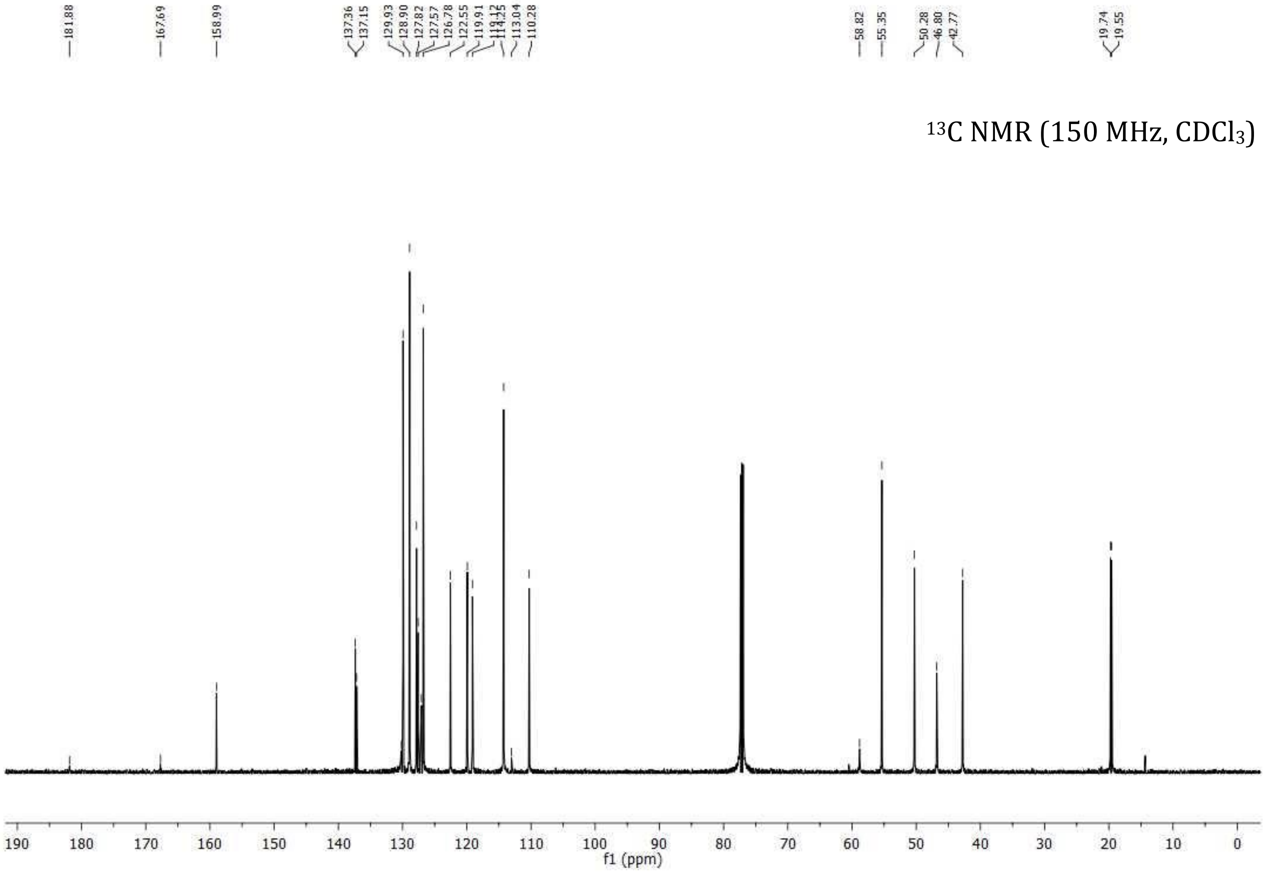

^{13}C NMR (150 MHz, $CDCl_3$)

5. ^{1}H and ^{13}C NMR spectra of imidamides **10a-f**

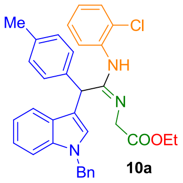

^{1}H NMR (600 MHz, $CDCl_3$)

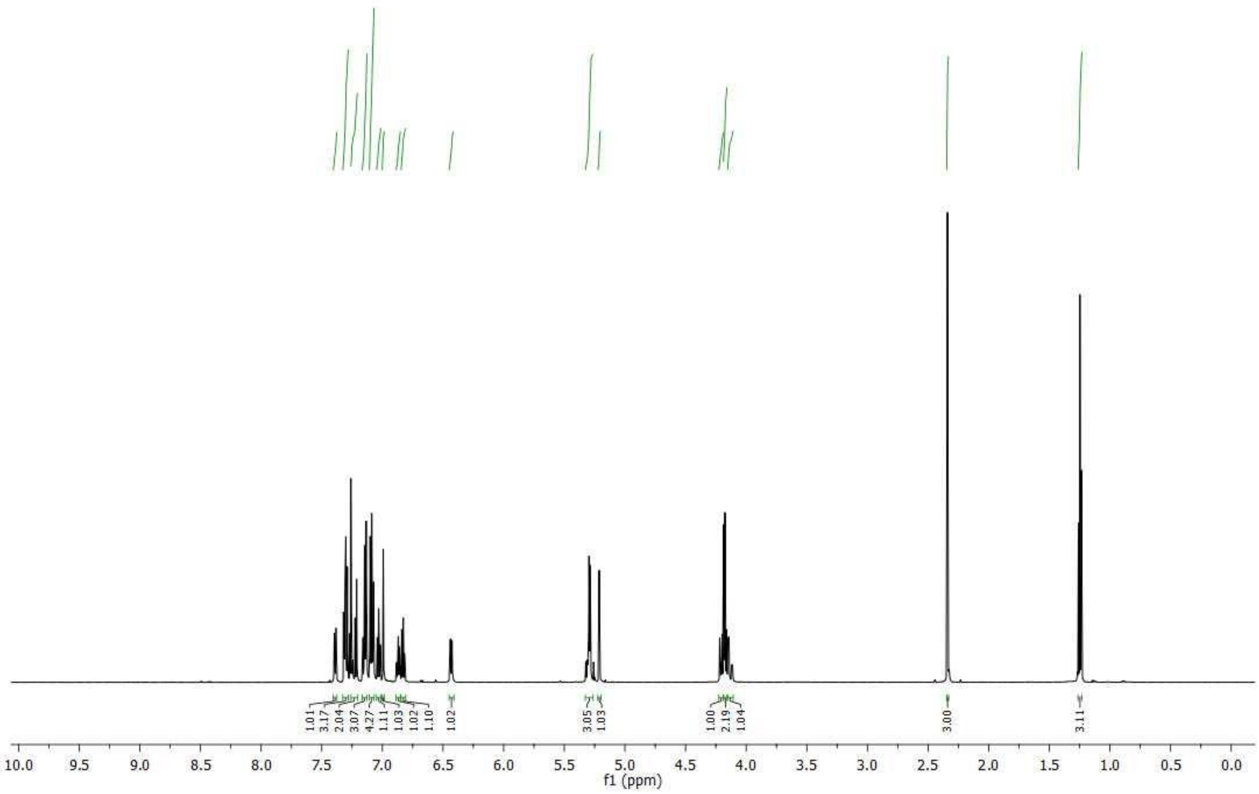

^{13}C NMR (150 MHz, $CDCl_3$)

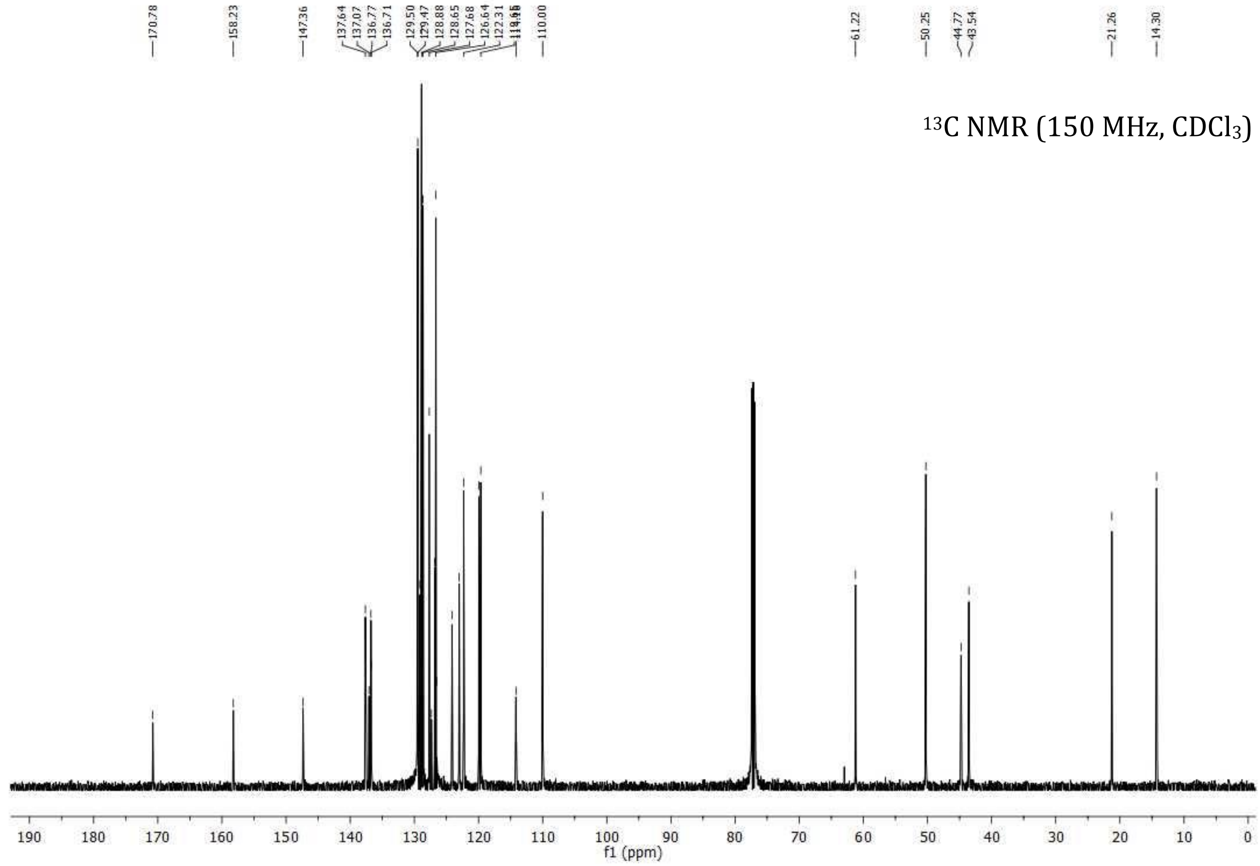

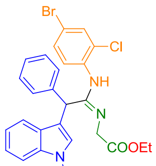

10b

^{1}H NMR (600 MHz, $CDCl_3$)

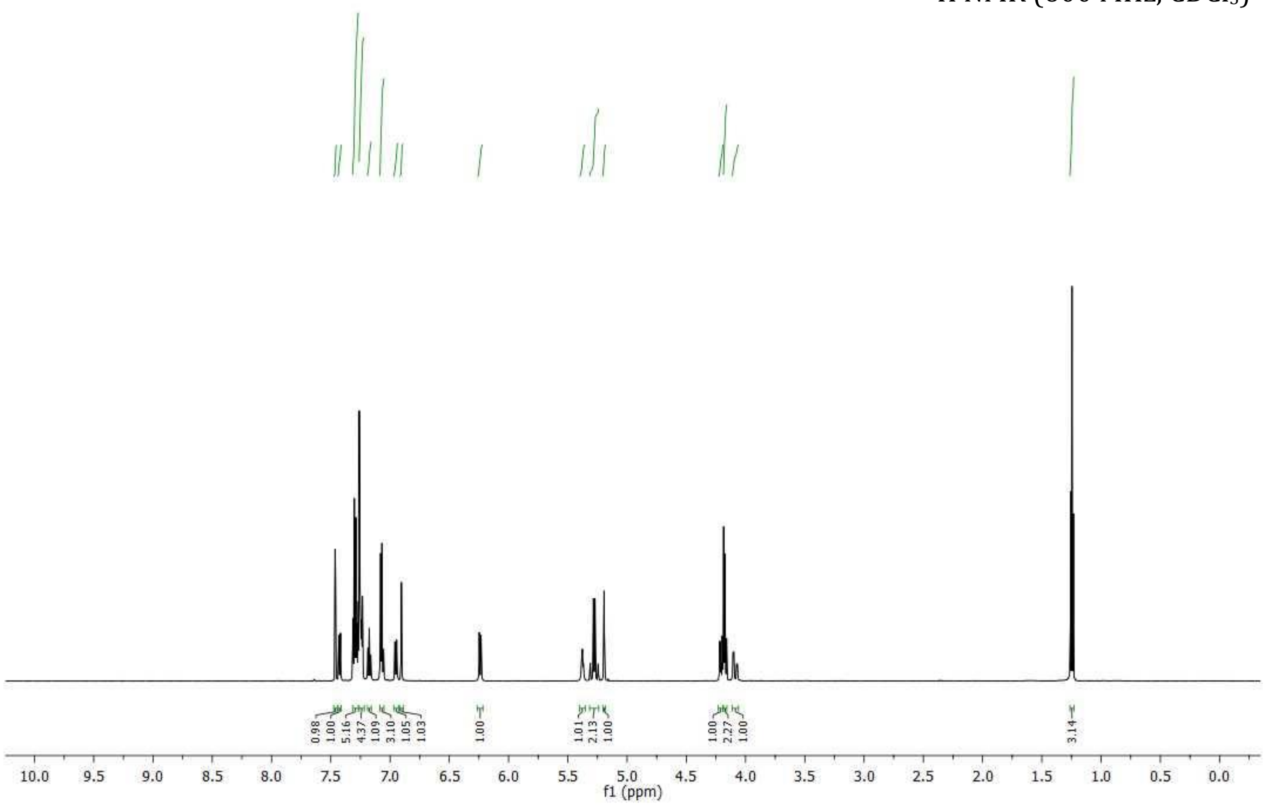

^{13}C NMR (150 MHz, $CDCl_3$)

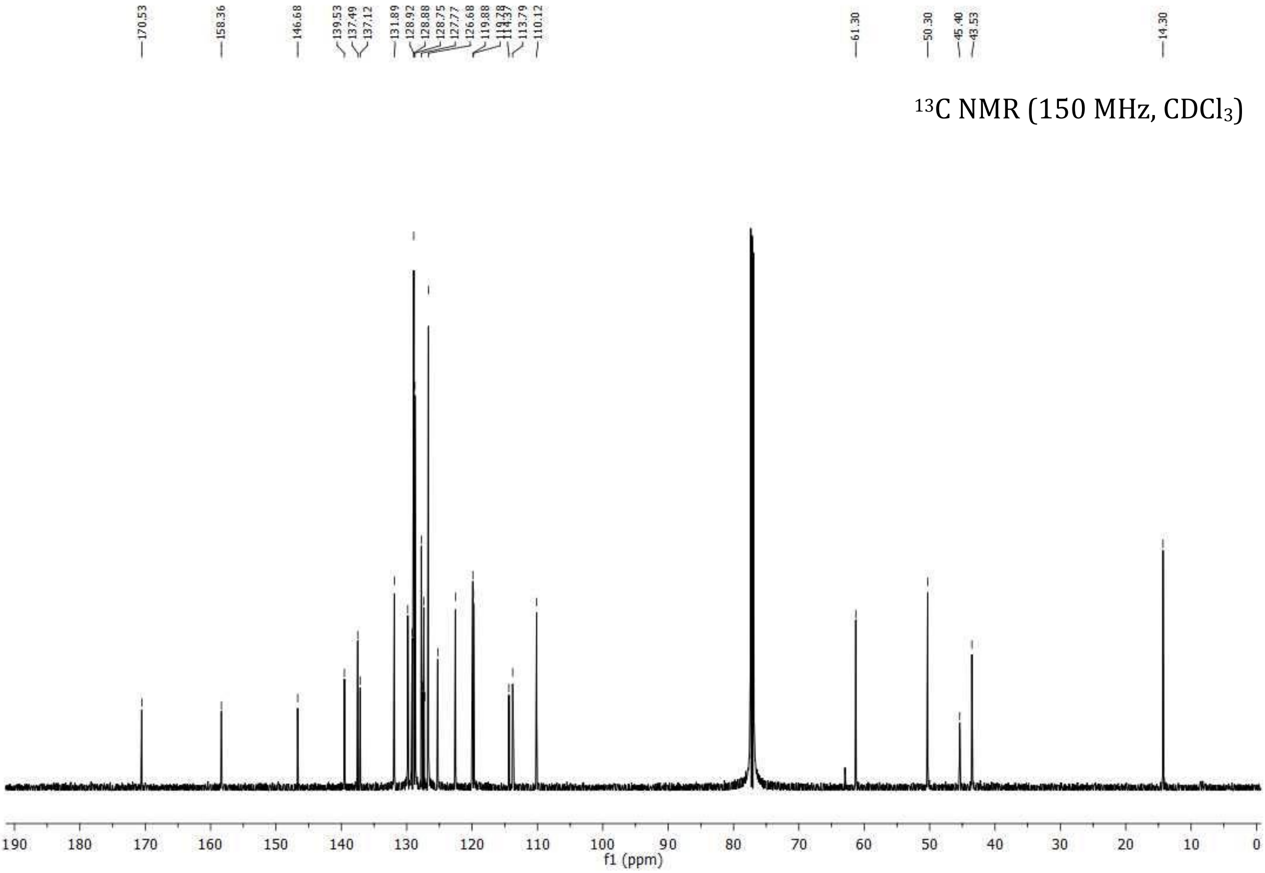

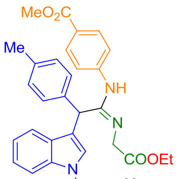

10c

^{1}H NMR (600 MHz, $CDCl_3$)

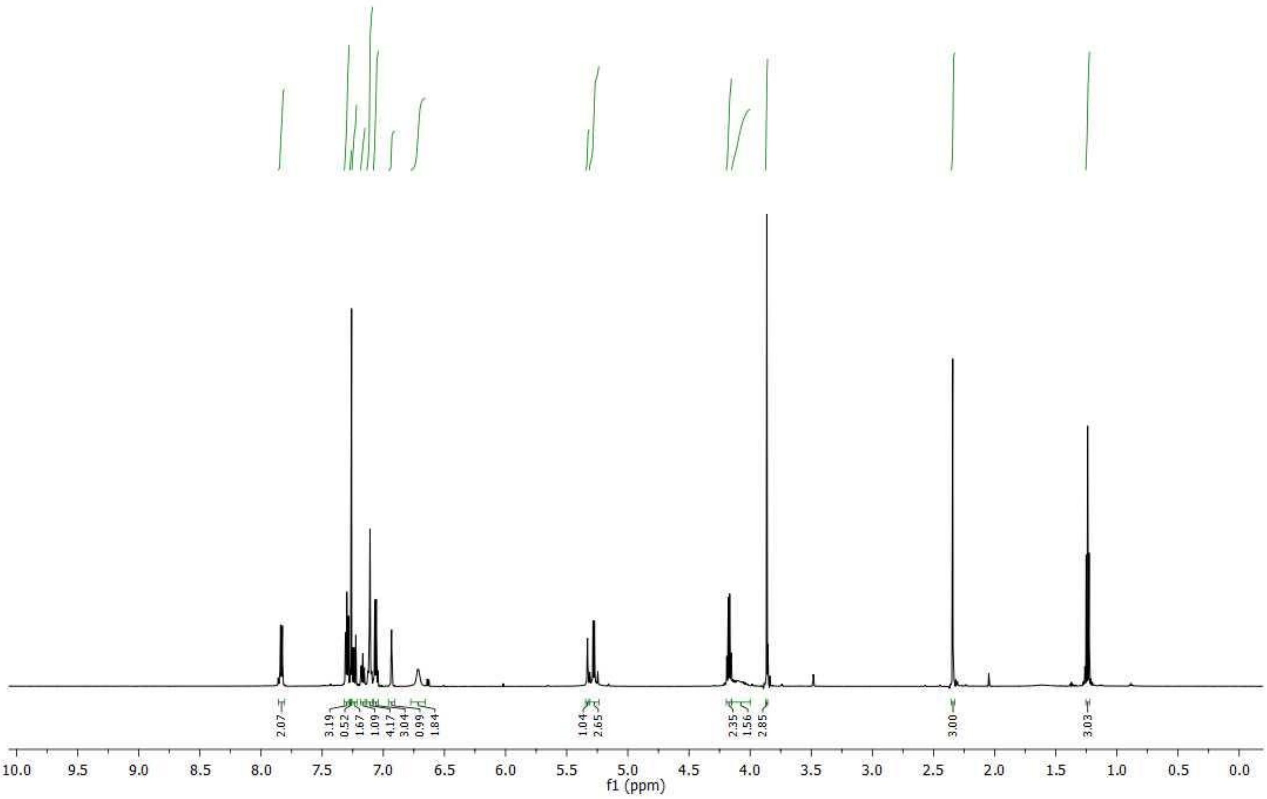

^{13}C NMR (150 MHz, $CDCl_3$)

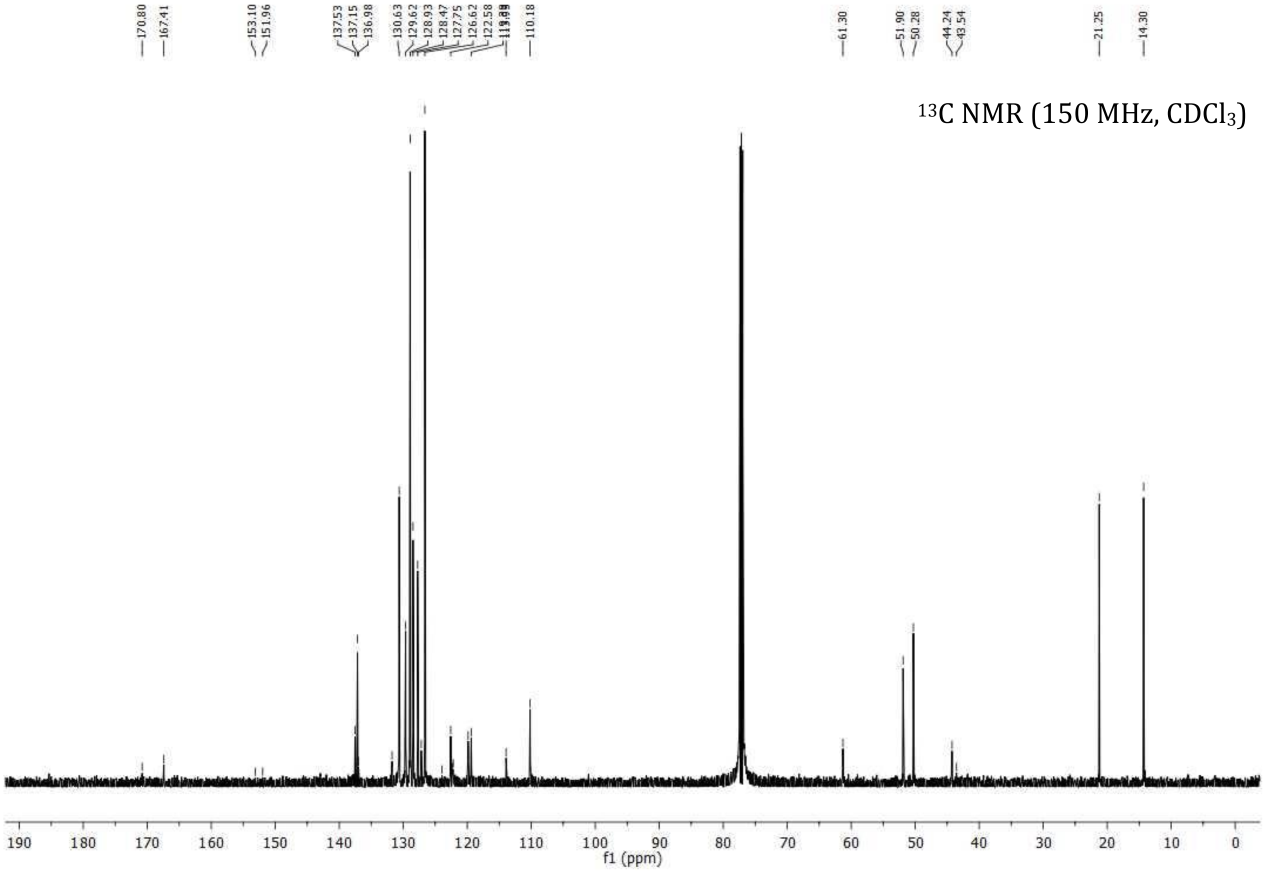

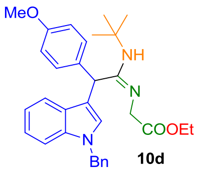

10d

^{1}H NMR (600 MHz, $CDCl_3$)

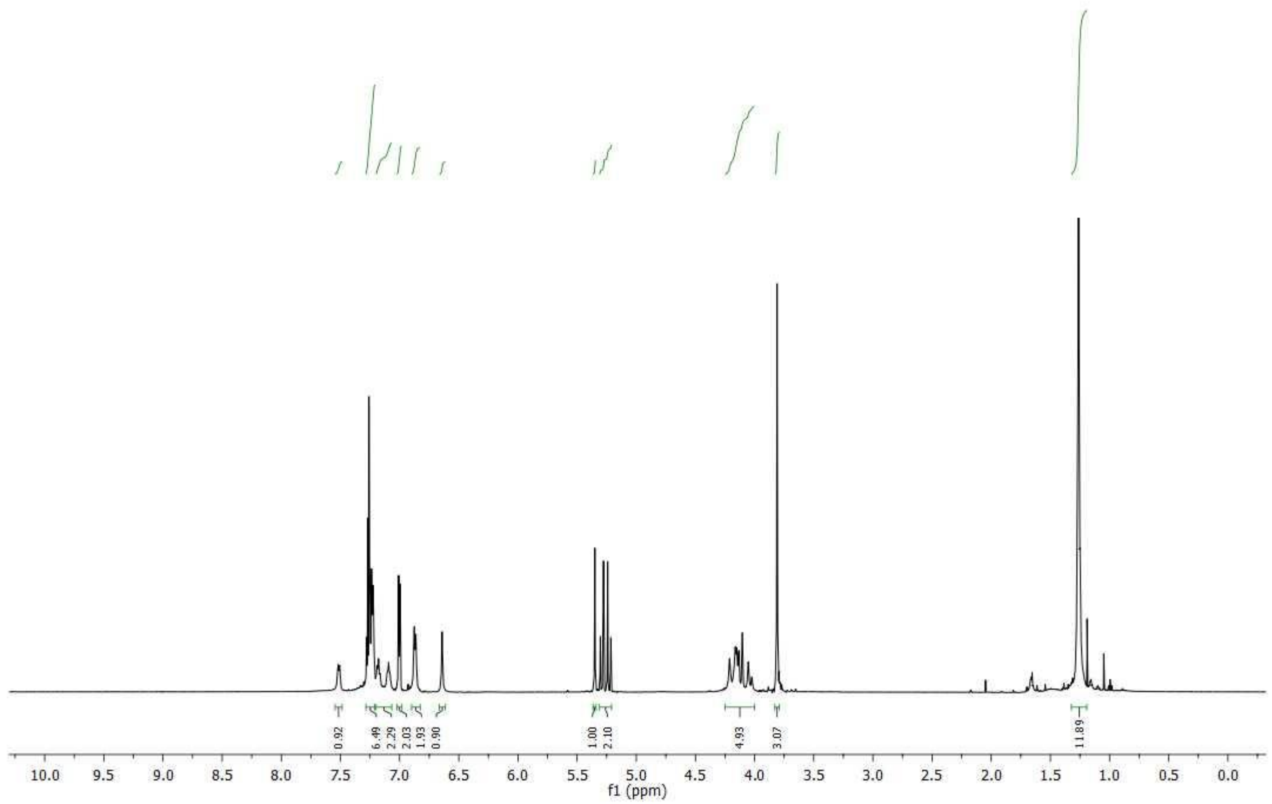

^{13}C NMR (150 MHz, $CDCl_3$)

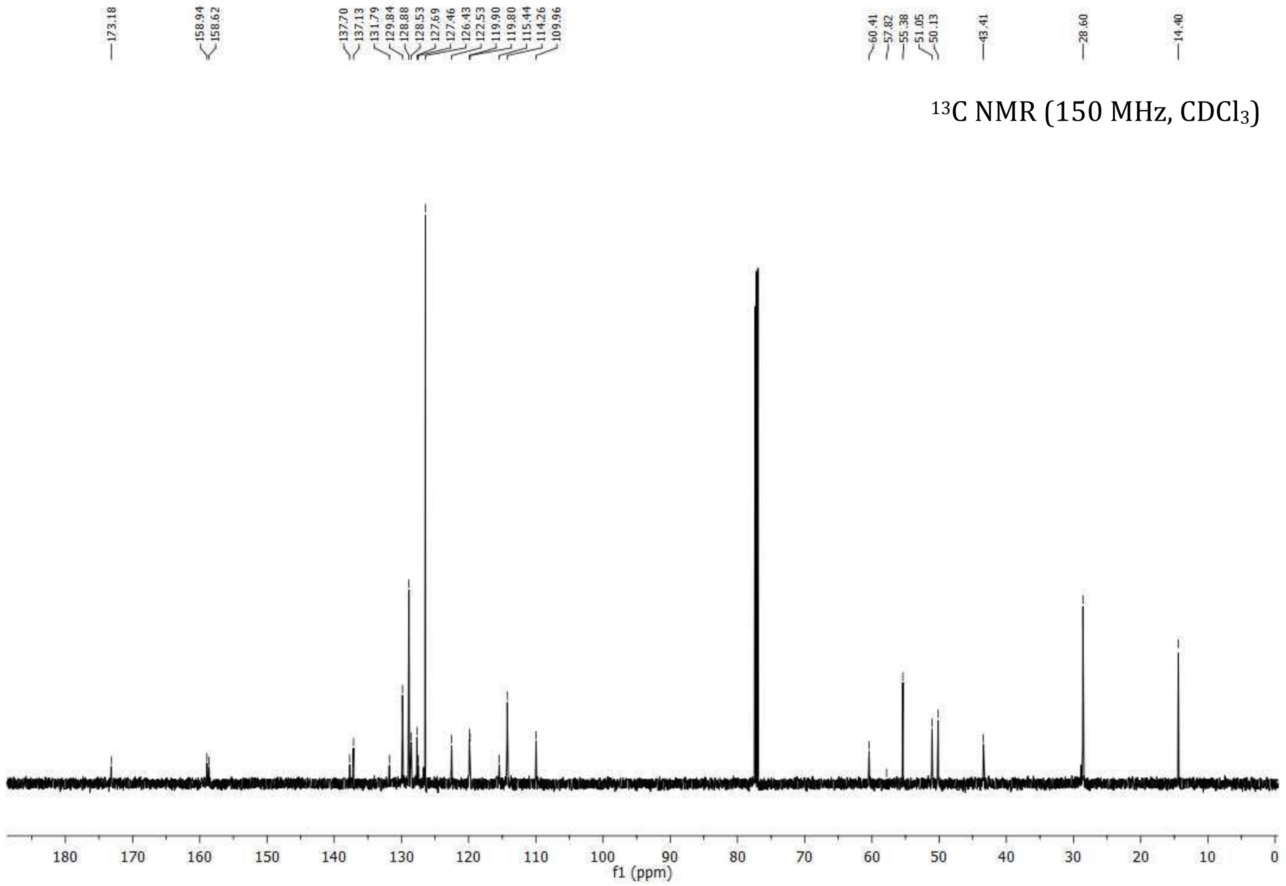

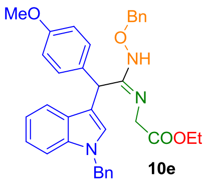

10e

^{1}H NMR (600 MHz, $CDCl_3$)

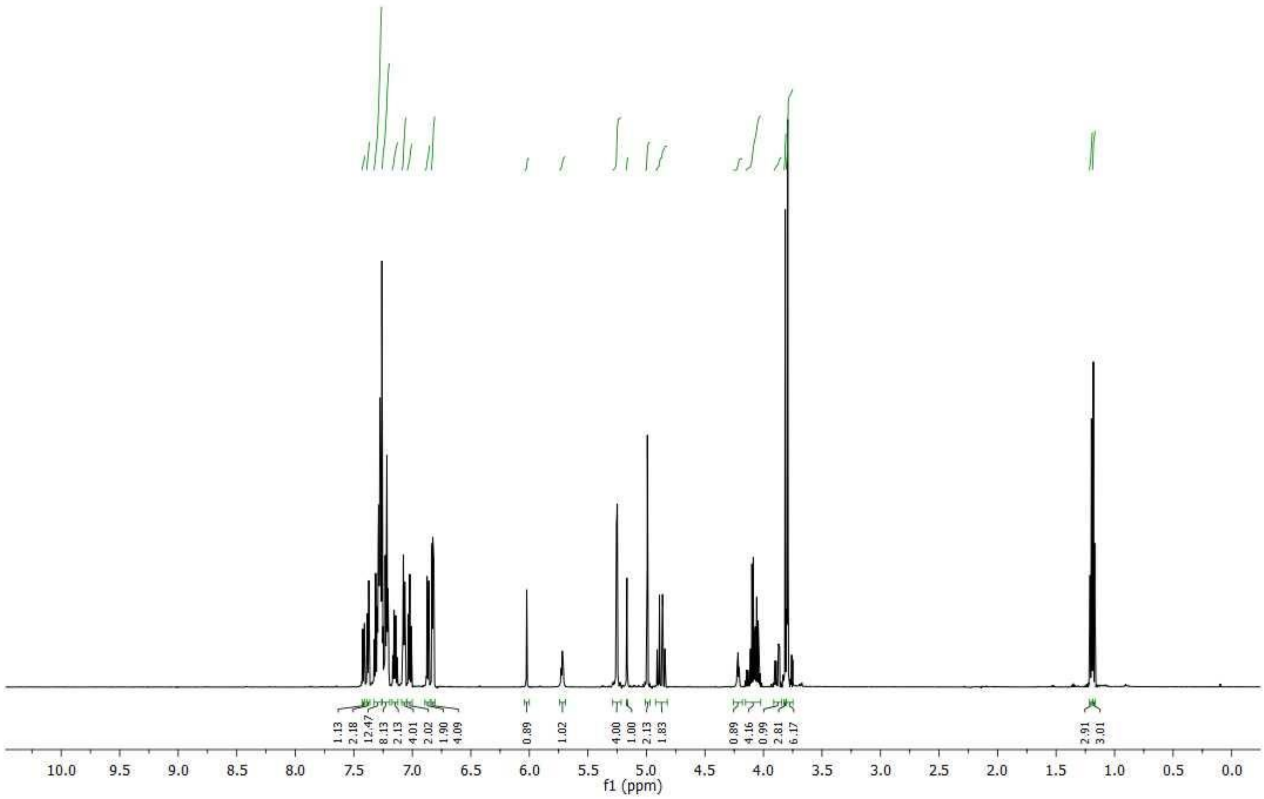

^{13}C NMR (150 MHz, $CDCl_3$)

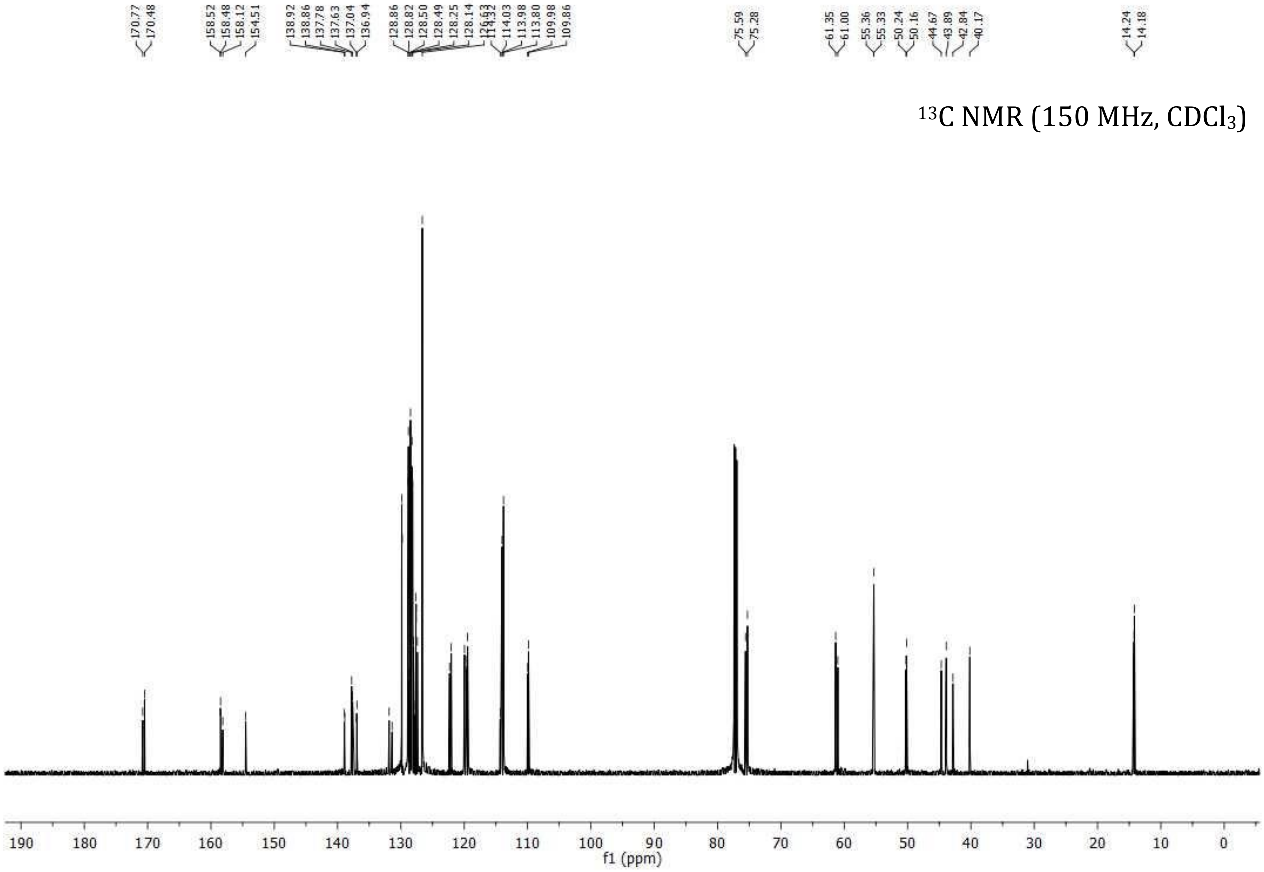

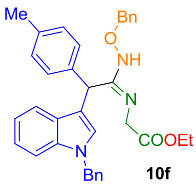

10f

^{1}H NMR (600 MHz, $CDCl_3$)

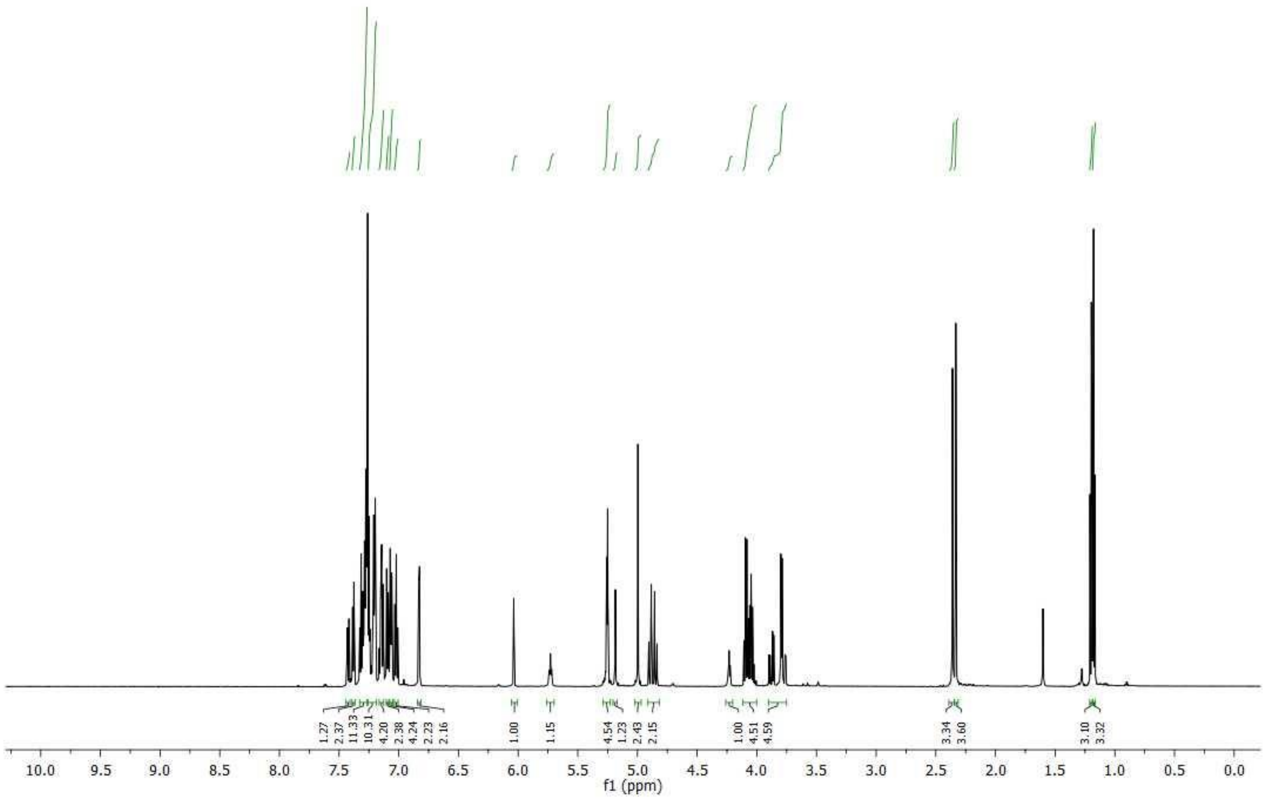

^{13}C NMR (150 MHz, $CDCl_3$)

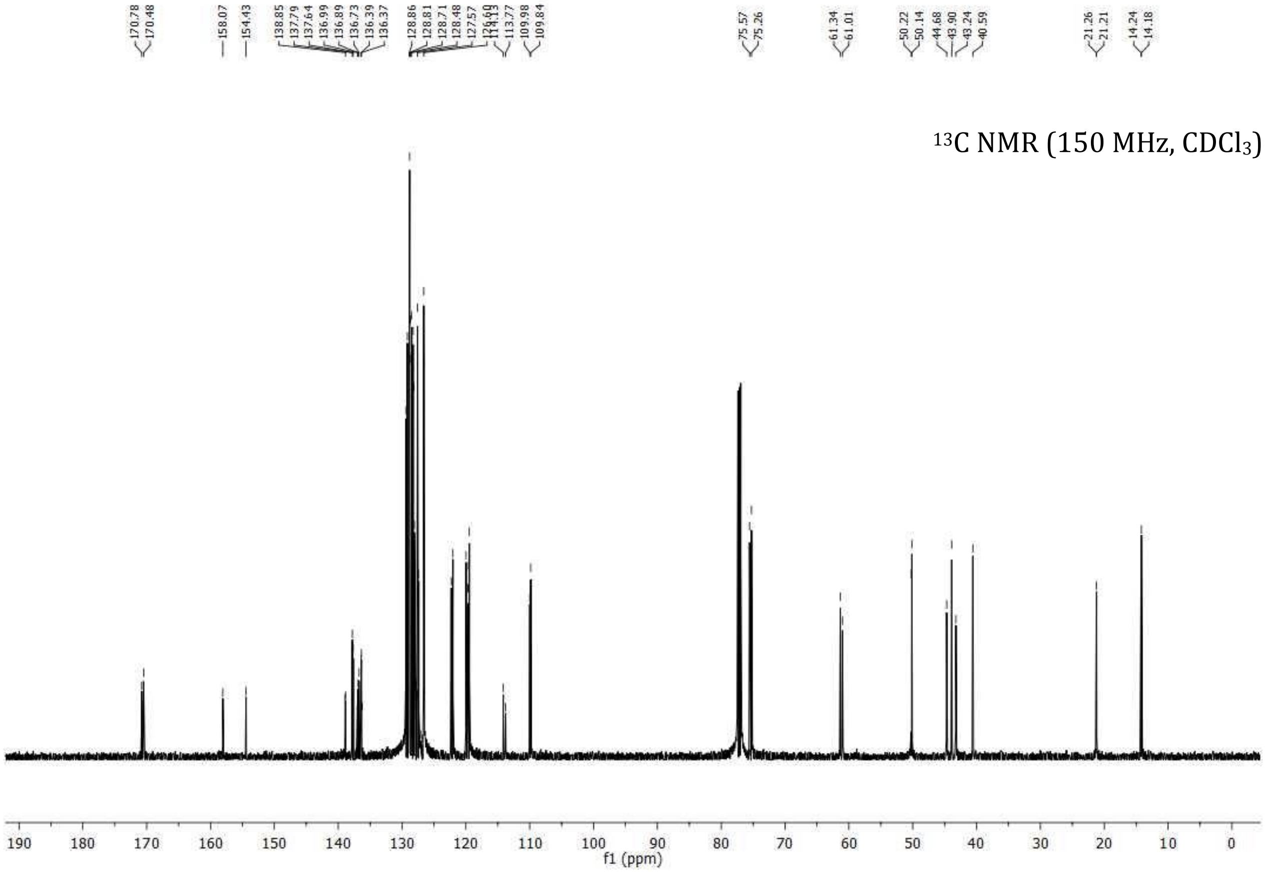

Supplement: Supplementary file 1 [file molecules-26-02402-s001.zip › molecules-1186865-supplementary.pdf]
